# Phase I trial of ribociclib with platinum chemotherapy in ovarian cancer

**DOI:** 10.1172/jci.insight.160573

**Published:** 2022-09-22

**Authors:** Lan G. Coffman, Taylor J. Orellana, Tianshi Liu, Leonard G. Frisbie, Daniel Normolle, Kent Griffith, Shitanshu Uppal, Karen McLean, Jessica Berger, Michelle Boisen, Madeleine Courtney-Brooks, Robert P. Edwards, Jamie Lesnock, Haider Mahdi, Alexander Olawaiye, Paniti Sukumvanich, Sarah E. Taylor, Ronald Buckanovich

**Affiliations:** 1 Magee-Womens Research Institute, UPMC Hillman Cancer Center, Pittsburgh, Pennsylvania, USA.; 2 Division of Gynecologic Oncology, Department of Obstetrics, Gynecology and Reproductive Sciences, Magee-Womens Hospital, Pittsburgh, Pennsylvania, USA.; 3 Division of Hematology/Oncology, Department of Medicine, Hillman Cancer Center, and; 4 Division of General Internal Medicine, Department of Medicine, University of Pittsburgh Medical Center, Pittsburgh, Pennsylvania, USA.; 5 Department of Integrative Systems Biology and; 6 Department of Biostatistics, University of Pittsburgh, Pittsburgh, Pennsylvania, USA.; 7 Center for Cancer Data Sciences, Rogel Cancer Center, and; 8 Division of Gynecologic Oncology, Department of Obstetrics and Gynecology, University of Michigan, Ann Arbor, Michigan, USA.

**Keywords:** Oncology, Cell cycle

## Abstract

**BACKGROUND:**

New therapeutic combinations to improve outcomes of patients with ovarian cancer are clearly needed. Preclinical studies with ribociclib (LEE-011), a CDK4/6 cell cycle checkpoint inhibitor, demonstrate a synergistic effect with platinum chemotherapy and efficacy as a maintenance therapy after chemotherapy. We tested the safety and initial efficacy of ribociclib in combination with platinum-based chemotherapy in recurrent ovarian cancer.

**METHODS:**

This phase I trial combined weekly carboplatin and paclitaxel chemotherapy with ribociclib, followed by ribociclib maintenance in patients with recurrent platinum-sensitive ovarian cancer. Primary objectives were safety and maximum tolerated dose (MTD) of ribociclib when given with platinum and taxane chemotherapy. Secondary endpoints were response rate (RR) and progression-free survival (PFS).

**RESULTS:**

Thirty-five patients were enrolled. Patients had a mean of 2.5 prior lines of chemotherapy, and 51% received prior maintenance therapy with poly(ADP-ribose) polymerase inhibitors and/or bevacizumab. The MTD was 400 mg. The most common adverse events included anemia (82.9%), neutropenia (82.9%), fatigue (82.9%), and nausea (77.1%). The overall RR was 79.3%, with a stable disease rate of 18%, resulting in a clinical benefit rate of 96.6%. Median PFS was 11.4 months. RR and PFS did not differ based on the number of lines of prior chemotherapy or prior maintenance therapy.

**CONCLUSION:**

This work demonstrates that the combination of ribociclib with chemotherapy in ovarian cancer is feasible and safe. With a clinical benefit rate of 97%, this work provides encouraging evidence of clinical efficacy in patients with recurrent platinum-sensitive disease.

**TRIAL REGISTRATION:**

ClinicalTrials.gov NCT03056833.

**FUNDING:**

This investigator-initiated trial was supported by Novartis, which provided drugs and funds for trial execution.

## Introduction

Ovarian cancer is the second-most common gynecologic malignancy in the United States and the eighth-most common malignancy among women worldwide (1, 2). In the United States, ovarian cancer is the deadliest of the gynecologic cancers, and it is thought to account for nearly 14,000 deaths in the year 2020 alone (1). While improvements have been made in ovarian cancer mortality rates, the 5-year relative survival rate in the United States continues to remain below 50% (1, 3, 4). Despite recent successes with new therapeutic agents, the majority of patients will develop progressive disease and require new lines of therapy.

Platinum-based chemotherapy regimens are the standard of care for the management of ovarian cancer, both as an initial treatment and as treatment of recurrent platinum-sensitive disease (defined as recurrence at or beyond 6 months after receiving platinum therapy) (5, 6). Recently, the use of maintenance therapy after treatment with platinum-based chemotherapy has demonstrated promising results. Maintenance therapy with poly-adenosine diphosphate–ribose (ADP-ribose) polymerase (PARP) inhibitors has significantly improved progression-free survival (PFS) rates after completion of platinum-based chemotherapy and has led to improved overall survival in women with deleterious *BRCA1* or *BRCA2* mutations (7). The vascular endothelial growth factor–targeting antibody, bevacizumab, has also been shown to improve PFS when used in the maintenance setting (7–9). However, critical considerations, including cost, tolerability, and availability of genomic testing, remain limiting factors in the use of these agents in the maintenance setting (9–15). Additionally, optimal treatment of women who progress after maintenance therapy is a critical, unanswered question. Thus, identification of new agents that are effective for the management of ovarian cancer remains essential.

CDK4/6 inhibitors are a class of novel therapeutics that have shown potential for use in multiple areas of oncology, including breast cancer, liposarcoma, mantle cell lymphoma, germ cell tumors, and non–small cell lung cancer (16–21). The FDA approved the CDK4/6 inhibitors ribociclib and palbociclib in combination with an aromatase inhibitor for first-line therapy in hormone receptor–positive, HER2-negative breast cancer (19, 21, 22). The pharmacologic reasoning for use of CDK4/6 inhibitors as an oncologic therapy is based on their ability to halt G_1_/S progression in the cell cycle (23). During G_1_, activation of CDK4 and CDK6 proteins triggers phosphorylation of the retinoblastoma (RB) protein, leading to release of RB-mediated E2F suppression and entry into the S phase (24). Negative regulation of CDK4 and CDK6 occurs via a combination of proteins, including p16 (23, 25). Genomic analyses of the CDK4/6 pathway in ovarian cancer have identified a high percentage of p16 deletions or downregulation, as well as increased mRNA expression of *CDK4* and *CDK6* (26). These genomic abnormalities can lead to abnormal cellular proliferation, as characterized by cancer cell growth; thus, the CDK4/6 pathway represents an appealing therapeutic target in this patient population. Interestingly, preclinical studies of the CDK4/6 inhibitor ribociclib in ovarian cancer demonstrated synergistic effects when combined with cisplatin, increasing ovarian cancer cell death (26). This initially appears paradoxical, as CDK4/6 inhibition blocks the G_1_/S phase transition and cisplatin works by inducing DNA damage in the S phase. However, the timing of ribociclib administration is critical. Dosing ribociclib concurrently or immediately after cisplatin enhances cisplatin-mediated cell death by prolonging cell cycle arrest in the S/G_2_/M phase and enhances DNA damage through p-CHK1 and pATR. Additionally, adding ribociclib maintenance after completion of platinum-based therapy delayed cancer cell recovery and growth in vitro and in vivo (26). These findings suggested a novel therapeutic strategy that sequences chemotherapy with CDK4/6 inhibition to enhance the efficacy of chemotherapy followed by CDK4/6 inhibitor maintenance to delay recurrence of ovarian cancer (26).

We therefore conducted a phase I trial to test the safety and efficacy of ribociclib (LEE-011) in patients with platinum-sensitive recurrent ovarian cancer. Patients had an average of 2.5 prior lines of therapy, and over half of the patients received prior maintenance therapy with a PARP inhibitor or bevacizumab. The primary endpoint of this study was to determine the maximum tolerated dose (MTD) of ribociclib when given concurrently with platinum and taxane chemotherapy. Secondary endpoints included response rates (RRs) and PFS. This study assesses a potentially novel combination of CDK4/6 inhibition with chemotherapy in ovarian cancer and demonstrates the safety and promising efficacy of sequencing this class of drugs with standard-of-care chemotherapy in ovarian cancer.

## Results

### Patient population.

Thirty-five patients were enrolled. Two patients were enrolled in the 200 mg ribociclib dosing group, and 33 patients were enrolled in the 400 mg ribociclib dosing group. Patient characteristics are described in Table 1. The majority (91.4%) of patients had high-grade serous histology, and the population reflected typical ovarian cancer demographics, with a mean age of 65 years and 17% *BRCA1*/*BRCA2* mutation carriers. The mean number of prior lines of chemotherapy was 2.4 (range, 1–8 prior lines; maintenance therapy was not included as a separate line of therapy). More than half of patients in the trial cohort had previously received bevacizumab and/or PARP maintenance therapy (*n* = 18 [51%]).

### Treatment.

The mean number of chemotherapy cycles per patient was 5.3 cycles. The mean number of maintenance ribociclib cycles was 10.2. Eleven patients remained actively on maintenance therapy at the time of analysis.

### Safety.

Dose-limiting toxicities (DLTs) were assessed during the first 2 cycles of chemotherapy. In the intention-to-treat population, no patients (0%) in the 200 mg dosing group and 11 patients (33.3%) in the 400 mg dosing group experienced a DLT. Thus, the MTD was determined to be 400 mg for concurrent therapy, and no patients were treated with 600 mg. At initiation of maintenance therapy, all patients received a 600 mg dose (the FDA-approved maintenance dose). The probabilities of DLT at the 2 dose levels used estimated by the time-to-event continual reassessment method (TITE-CRM) model were ultimately 0.144 (95% CI = [0.078–0.24]) for level 1 (200 mg) and 0.263 (95% CI = [0.166–0.384]) for level 2 (400 mg).

Adverse events (AEs) were collected for the full duration of the trial (both chemotherapy and maintenance phases). Overall, grade 3 AEs were experienced by 22 patients (62.9%), while grade 4 AEs were experienced by 11 patients (31.4%). In the 200 mg ribociclib dosing group, 1 patient (50%) experienced a grade 3 AE and 1 patient (50%) experienced a grade 4 event. In the 400 mg dosing group, 21 patients (62.9%) experienced a grade 3 AEs and 10 patients (31.4%) experienced a grade 4 AE. One patient experienced a grade 5 AE, possibly related to the study drug. This patient experienced a fall at home and did not seek medical treatment for 24 hours and subsequently died. In-depth investigation surrounding this event revealed that the patient had adequate blood counts (neutrophil count, >1,000; platelet count, >100,000) and normal EKG, with a normal QTcF interval directly prior to the event; therefore, it was unlikely due to neutropenic infection, bleeding related to thrombocytopenia, or prolonged QTc. Grade 3 and 4 AEs were more common during concurrent chemotherapy (n = 33 events) than during maintenance therapy (*n* = 9 events). The most commonly experienced AEs are described in Table 2. The most common grade 3 and 4 AEs were hematologic, including leukopenia (*n* = 19, 54.3%), neutropenia (*n* = 19, 54.3%), lymphopenia (*n* = 8, 22.9%), anemia (*n* = 6, 17.1%), and thrombocytopenia (*n* = 6, 17.1%). However, there was only 1 occurrence of grade 3 and 4 febrile neutropenia. Other common AEs were fatigue, nausea, and hypertension, although grade 3 and 4 AEs in these categories were rare. Notably, a grade 3 and 4 prolonged QTcF interval was only experienced by 1 patient.

### Efficacy.

**Figure 1:**
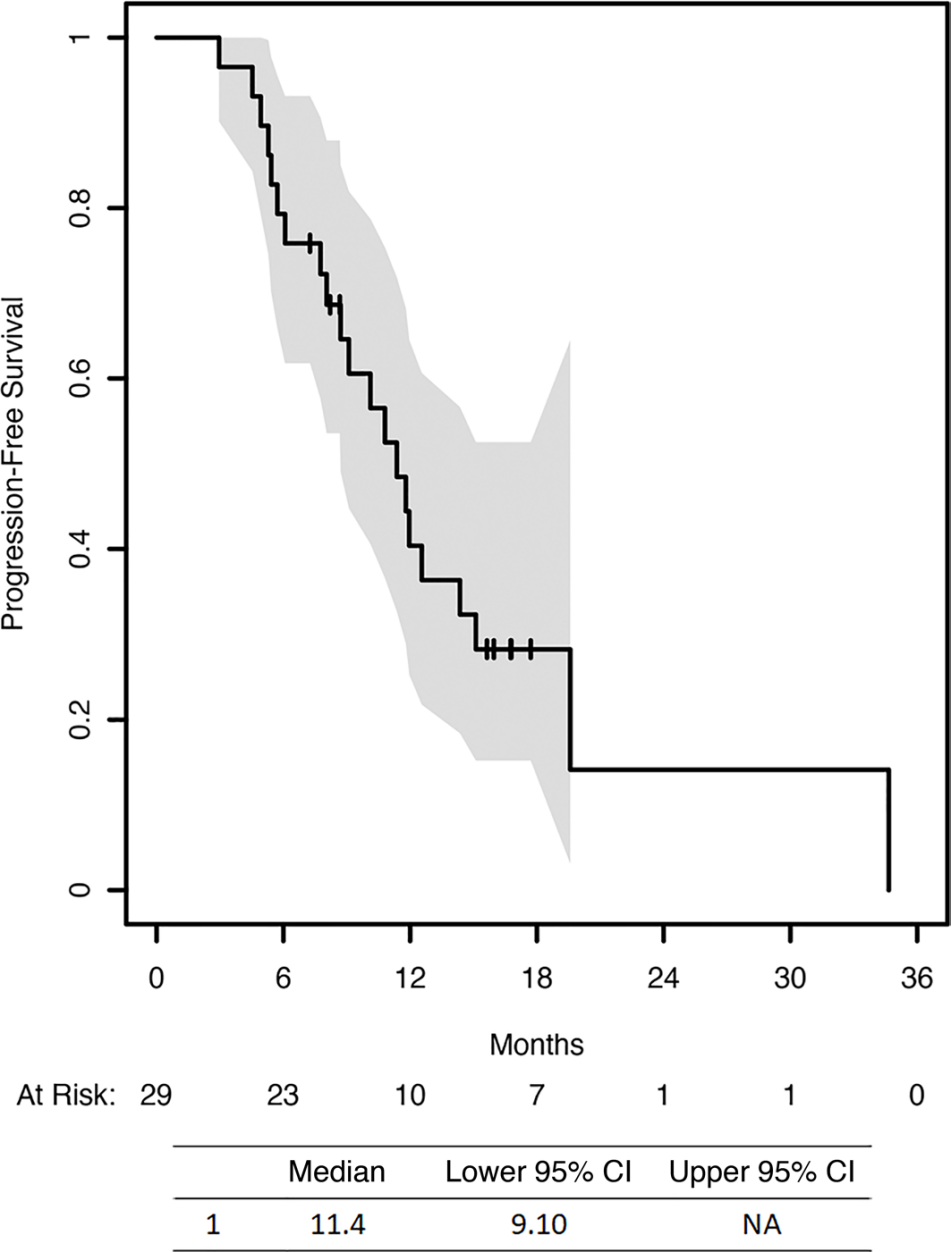
Progression-free survival.

We were able to evaluate 29 patients for response (4 patients withdrew from the trial prior to first-response evaluation via CA125 or CT scan). Nineteen patients (65.5%) demonstrated a partial response, and 4 patients (13.8%) demonstrated a complete response to therapy. Therefore, the overall RR was 79.3% (2 patients in 200 mg group, 21 patients in 400 mg group). When including patients with stable disease (*n* = 5), the overall clinical benefit rate was 96.6% (Table 3). Median PFS was 11.4 months (95% CI = [9.10 months, not reached]) (Figure 1). Among patients who received maintenance therapy, the median PFS during the maintenance phase was 9.4 months.

**Figure 2:**
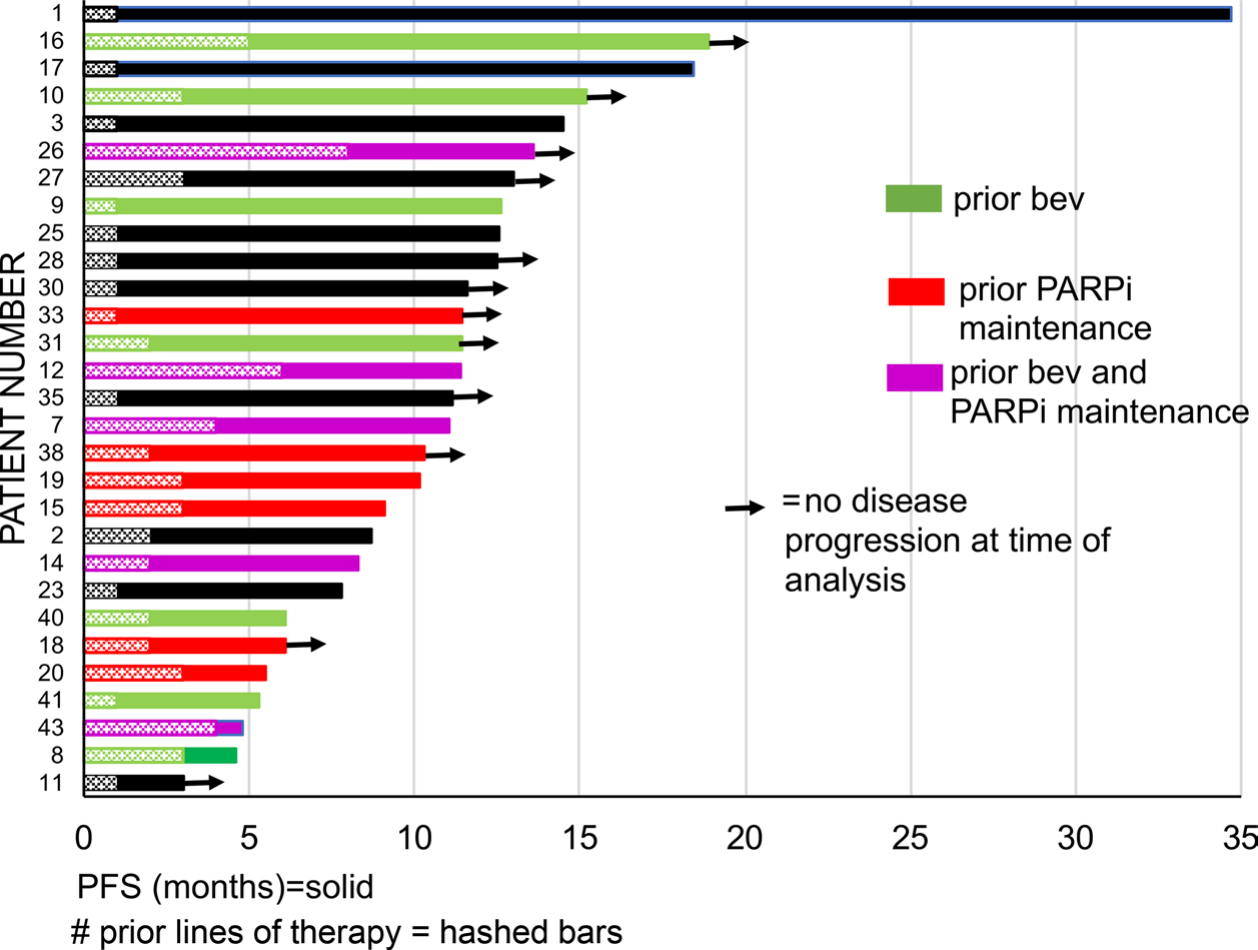
Swimmers plot of individual patient progression-free survival. Bars with solid colors indicate prior maintenance therapy: green indicates prior bevacizumab (bev) therapy; red indicates prior poly(ADP-ribose) polymerase inhibitor (PARPi) therapy; purple indicates prior bev and PARPi therapy; and black indicates no prior therapy. Bars with hashed marks represent the number of prior lines of therapy. Arrows indicate that no disease progression was found at the time of analysis. Number on the *x* axis indicate months of progression-free survival.

On exploratory analysis, there was no significant difference in PFS between patients who had received prior maintenance PARP inhibitor (PARPi) or bevacizumab therapy compared with those without prior maintenance therapy (median PFS, 10.12 months with prior maintenance versus 14.36 months without, *P* = 0.068). Figure 2 summarizes per patient PFS by prior maintenance therapy and number of lines of prior therapy. The average number of lines of prior therapy in the 29 patients evaluable for response was 2.4. Proportional hazards (Cox) regression demonstrated that the number of prior lines of chemotherapy was not significantly associated with PFS (HR, 0.996; 95% CI = [0.77–1.29], *P* = 0.97). We additionally compared the PFS of patients on trial to PFS on their most recent therapy prior to trial enrollment. We found that 37% of patients had either improved PFS compared with that on prior therapy or had not yet progressed. We performed a proportional hazards (Cox) regression on the current trial PFS with prior PFS as a predictor. We found that prior PFS was not significantly associated with current trial PFS (HR, 0.9945; 95% CI = [0.96–1.03], *P* = 0.75). Additionally, both prior maintenance therapy (χ^2^ test, *P* = 0.14) and the number of lines of prior therapy (χ^2^ test, *P* = 0.31) were not significantly associated with clinical response.

Given the hypothesized resistance to CDK4/6 inhibitors in patients with RB tumor-suppressor gene (*RB1*) mutations, we also sought to correlate responses to *RB1* mutational status. Paraffin-embedded tumor samples were available for 30 patients (86%). *RB1* sequencing was performed on all 30 samples. After gene analysis, 1 patient (3.33%) was found to have a clinically relevant *RB1* mutation; however, her disease was not evaluable for response due to withdrawal from trial.

## Discussion

This phase I trial demonstrates that ribociclib can be safely administered in the population of patients with platinum-sensitive ovarian cancer, both sequenced concurrently with platinum and taxane chemotherapy and as maintenance therapy. Ribociclib was associated with a higher occurrence of grade 3 and 4 hematologic AEs when sequenced with cytotoxic chemotherapy. However, only 1 patient experienced grade 3 and 4 febrile neutropenia.

These safety data are consistent with those in prior studies of ribociclib (19, 21, 27, 28). In the MONALEESA-2 trial, ribociclib was administered concurrently with letrozole for patients with advanced or recurrent hormone receptor–positive, HER2-negative breast cancer (19). In the updated results from that trial, 52.4% and 9.6% of patients experienced grade 3 and 4 neutropenia, and 20.1% and 1.2% of patients experienced grade 3 and 4 leukopenia, respectively (27). A recent phase II study of combined ribociclib and letrozole for relapsed estrogen receptor–positive ovarian and endometrial cancer reported that 60% of patients experienced grade 3–5 AEs during the trial (28). Additionally, CDK4/6 inhibitors are actively being studied in combination with chemotherapy in other solid malignancies to test similar potential synergistic effects, as proposed in this study (29). A phase I trial using palbociclib in combination with paclitaxel was conducted in breast cancer (30). Palbociclib was administered after paclitaxel in a fashion similar to our dosing schedule, with paclitaxel given on days 1, 8, 15, and 22, followed by palbociclib on days 2–6, 9–13, and 16–20 in 28-day cycles. The most common grade 3 and 4 AE was neutropenia (22% after the first cycle at the calculated recommended phase II dose of palbociclib).

In addition to being safely administered during concurrent therapy, ribociclib was also well tolerated as maintenance therapy. Only 9 grade 3 and 4 AEs occurred on maintenance therapy during this trial, as compared with 33 events during concurrent administration with platinum and taxane chemotherapy. Due to the prolonged time course over which maintenance therapy is typically administered, the tolerability of the maintenance regimen is a key component of use. Advantages of ribociclib over other maintenance regimens include oral administration and a mechanism of action that is not dependent on *BRCA1/BRCA2* or homologous recombination status.

Efficacy was a secondary objective of this study. While it is important not to overinterpret results on small single-institution studies, the efficacy results are encouraging. In the overall cohort, RR was 79.3%, with a clinical benefit rate of 96.6%. Previous reports demonstrate that platinum-based chemotherapy alone achieved a RR of 54%–66%, while platinum-based chemotherapy in combination with bevacizumab achieved a RR of 78.5% in the platinum-sensitive recurrent ovarian cancer setting (9, 31). This high RR validates the preclinical work that demonstrated a unique synergy with ribociclib sequenced after cisplatin chemotherapy enhancing cisplatin-mediated cytotoxicity (26). Furthermore, median PFS was 11.4 months. ≥This strong PFS may be a result of both a deepened cytotoxic response with ribociclib plus chemotherapy, as well as delayed progression with ribociclib maintenance. While cross-trial comparisons are fraught, these findings are similar to those of the OCEANS trial, which was a landmark study assessing the addition of bevacizumab to gemcitabine and carboplatin, followed by bevacizumab maintenance in platinum-sensitive recurrent ovarian cancer (9). The investigators found that the bevacizumab arm had a significantly improved PFS of 12.4 months compared with the placebo arm (PFS of 8.4 months), which is similar to the findings in this trial of ribociclib given concurrently with chemotherapy and as a maintenance regimen (9). However, in the OCEANS trial, only 1 prior line of therapy was allowed, whereas the number of prior lines of therapy in this study ranged from 1 to 8, with an average of 2.4 (with maintenance therapy not included as a line of therapy) (9). Our patient cohort aligned with the expected demographics in epithelial ovarian cancer (largely high-grade serous histology, average age of 65 years, and 17% *BRCA1*/*BRCA2* mutation rate) but was more heavily pretreated than that in the OCEANS trial cohort. Overall, our results are encouraging and merit further investigation in a larger patient cohort.

Given the paucity of data on treatment response after maintenance therapy in patients with ovarian cancer, an exploratory analysis was performed to assess PFS in patients with and without prior maintenance therapy. Interestingly, there was no statistically significant difference in PFS between the groups. On further analysis, there was also no statistically significant association between the number of prior lines of therapy and PFS. Additionally, over 30% of patients had an improved PFS on trial compared with their most recent prior PFS. This is surprising, as most patients retreated with chemotherapy will have reduced benefit with each subsequent therapy (as indicated by a decreased PFS with each successive course of therapy). While not the primary aims of this study, these results suggest that ribociclib may have activity in patients who have received multiple prior lines of chemotherapy or who have previously received another maintenance regimen. This is particularly important, given the ongoing clinical need for treatment approaches for the increasing number of patients that are receiving PARPi or bevacizumab early in their treatment course.

In conclusion, this trial demonstrates that ribociclib can be safely administered concurrently with chemotherapy in patients with recurrent ovarian cancer. Although safety was the primary objective of this trial, the RR and PFS data suggest substantial activity of ribociclib against ovarian cancer in combination and following platinum-based chemotherapy. This work strongly supports the further investigation of ribociclib for the treatment of ovarian cancer.

## Methods

This study was a phase I, open-label, single-institution dose-escalation trial of ribociclib with platinum-based chemotherapy in recurrent platinum-sensitive ovarian cancer (NCT03056833).

### Patients.

Eligible patients were women of at least 18 years of age with platinum-sensitive recurrent ovarian, fallopian, or primary peritoneal cancer. Platinum-sensitive disease was defined as recurrent disease more than 6 months after completion of the last platinum-based chemotherapy. All epithelial histologies (high-grade serous, endometrioid, clear cell, carcinosarcoma, and low-grade serous) were included. Patients were recruited from the University of Michigan and UPMC cancer clinics. To participate, patients had to be able to provide informed consent and comply with all study protocols. Patients were required to have completed at least 1 prior line of platinum-based therapy and to have an Eastern Cooperative Oncology Group performance score of 0–1, with a life expectancy of at least 3 months. Disease progression or recurrence was defined by the Gynecologic Cancer InterGroup Response Evaluation Criteria in Solid Tumors criteria (32). Before trial enrollment, patients were required to undergo laboratory screening for adequate renal, hepatic, hematologic, and electrolyte parameters and were required to have a pretreatment electrocardiogram demonstrating a QTcF interval of less than or equal to 450 ms (using Fridericia’s correction).

Patients were excluded based on the following criteria: borderline or low-malignant potential histology, platinum-resistant disease, grade 3 baseline neuropathy, prior use of CDK4/6 inhibitors, congenital long QT syndrome or family history of unexpected sudden cardiac death, clinically significant uncontrolled heart disease or cardiac repolarization abnormalities, history of HIV infection, current pregnancy or lactation, impairment of gastrointestinal function that might alter absorption of the study drugs, or concurrent malignancy or malignancy within 3 years prior to starting the study drug (with the exception of adequately treated basal or squamous cell carcinoma, nonmelanomatous skin cancer, or curatively resected cervical cancer). Patients were also excluded if they were currently receiving warfarin or other coumadin-derived anticoagulants (low-molecular-weight heparin and fondaparinux were allowed), currently receiving or had received systemic corticosteroids within 2 weeks of starting the study drug, had major surgery within 14 days prior to starting study drug or had not recovered from major side effects, or had participated in a prior investigational study within 30 days of enrollment or 5 half-lives of the investigational product (whichever was longer).

### Study design.

**Figure 3:**
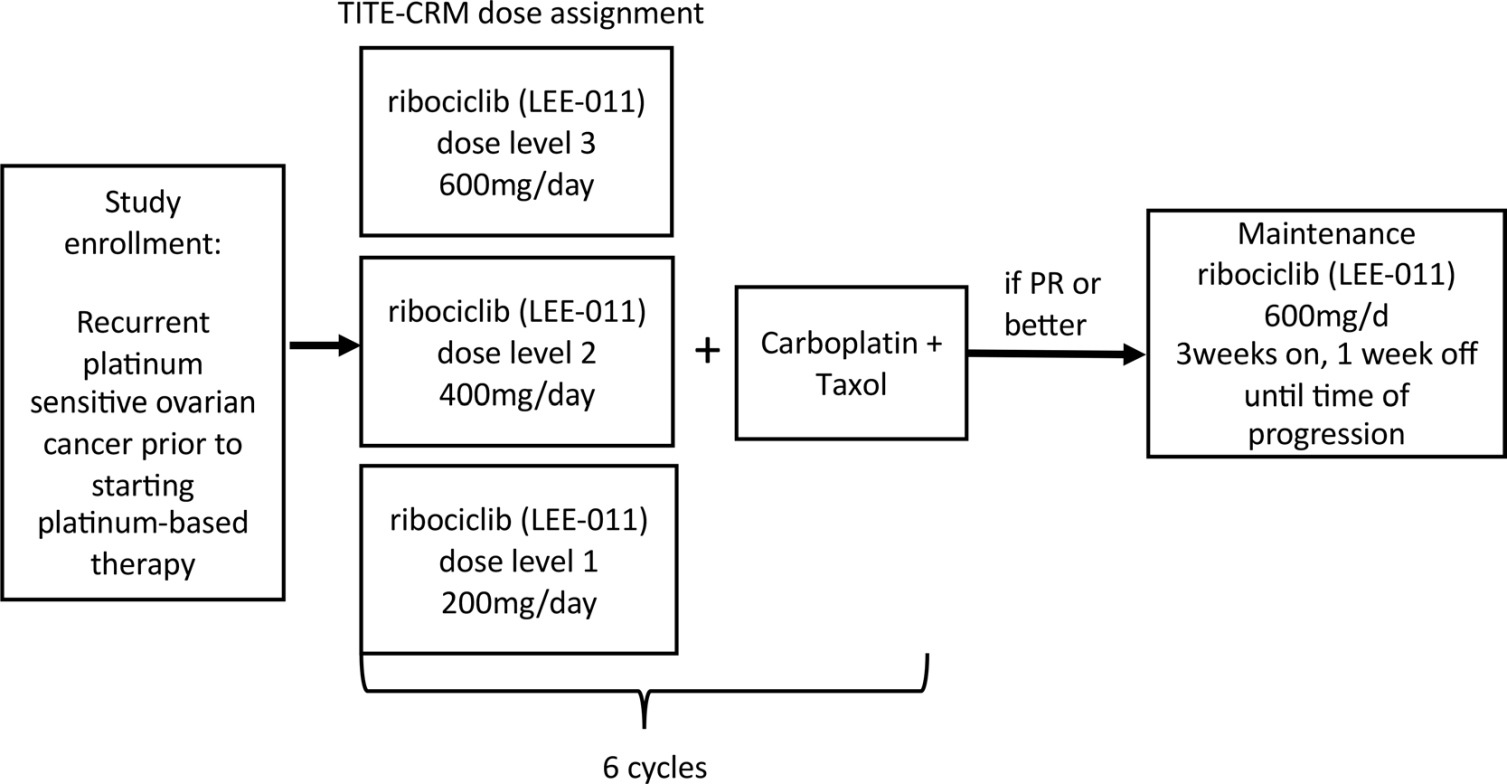
Time-to-event continual reassessment method study schema. TITE-CRM, Time-to-event continual reassessment method.

The primary endpoint of this study was to determine the MTD of ribociclib when given with platinum and taxane chemotherapy. Secondary endpoints were RR and PFS. Dose assignment was determined using TITE-CRM (Figure 3) (33, 34). Ribociclib dosing levels were (a) 200 mg, (b) 400 mg, and (c) 600 mg. The target rate for the MTD was set at 0.25, and the standard deviation of the dose-toxicity parameter was assigned a value of 0.1, which was found to work well in simulation studies. The probabilities of DLT at the 3 dose levels were originally estimated to be 0.1, 0.2, and 0.3, respectively. The trial was originally designed to accrue 40 participants; however, the study was closed to accrual after enrolling 35 patients due to COVID-19 limitations and high confidence in the recommended phase II dose. Attrition rates and reason for coming off trial were documented for all participants. For the first 15 participants, assignment to a dose level with probability of toxicity 0.05 greater than the target rate was permitted; afterwards, assignment was allowed only to a dose with probability of toxicity less than or equal to the target rate. Ribociclib dosing was initiated at level 1 (200 mg), and dose escalation was determined for newly enrolled patients based on the TITE-CRM algorithm. The 200 mg dose was chosen as the initial starting point for concurrent therapy, as ribociclib has not previously been tested in combination with platinum and taxane chemotherapy. Dose-limiting toxicities were assessed during the first 2 cycles of chemotherapy (first 8 weeks of the study). Safety and toxicity data were followed for the duration of the study (including chemotherapy and maintenance portions).

### Treatment.

Enrolled patients were treated with ribociclib in combination with weekly carboplatin AUC2 and 60 mg/m^2^ paclitaxel (35). Carboplatin and taxane chemotherapy were administered on days 1, 8, and 15 of a 28-day cycle, and ribociclib was administered on days 1–4, 8–11, and 15–18 after receiving chemotherapy (with chemotherapy given in the morning and ribociclib started that night). Therapy was planned for a total of 6 treatment cycles. Ribociclib was administered orally, and platinum and taxane chemotherapy were administered intravenously per standard dosing. Patients who achieved at least a partial response after completion of chemotherapy then received maintenance ribociclib at 600 mg daily, starting within 6 weeks of completion of chemotherapy and continuing (3 weeks on, 1 week off) until time of progression. Patients were followed for at least 18 months from the time of enrollment.

### Correlative studies.

*RB1* sequencing was performed on patient tumor samples. DNA extraction was performed on paraffin-embedded tumor samples using the Qiagen DNeasy Blood and Tissue Kit. DNA samples then underwent next-generation sequencing. After sequencing, the reads were demultiplexed and trimmed for adapter sequences and quality. Reads were then mapped to the reference genome GRCh37 (hg19) using *CLC Genomics Workbench 20* (Qiagen) core read mapping algorithm with default settings. To better align the reads after mapping, multipass local realignment using the default tools in *CLC* was done, and the resulting maps were compared with the targeted amplicon region to ensure quality coverage. Variants were called using *CLC*’s low-variant detection tool to screen for germline and somatic variants. A significance level of α = 0.05 was used for the error model, and variants with a frequency of more than 3% were called. Resulting variants were filtered for count and coverage (>3 and >19, respectively), forward-reverse read balance (no zeros), and significance (QUAL > 30). Clinical significance of the filtered variants was assessed by annotating to the ClinVar (RRID:SCR_006169) and dbSNP (RRID:SCR_002338) databases (NCBI).

### Data availability.

All data related to this manuscript, including the full clinical protocol, were included in the submission. DNA-sequencing data will be made available upon reasonable request upon confirmation from the University of Pittsburgh’s IRB.

### Statistics.

A TITE-CRM statistical design was utilized for this study. The TITE-CRM (33, 34) is a modification of the continual reassessment method (36) that employs a Bayesian model that is weighted to account for trial participants who have not been observed to failure. As patients were enrolled, dosing was assigned based on the survival of the patients currently on the trial. Toxicities were tabulated at all dose levels, although levels 1 and 2 were the only ones tested. The dose-toxicity function was estimated. Because only 2 of the 35 toxicity-evaluable participants were treated at dose level 1, RR was calculated without respect to dose (with a 95% exact credible interval), as was PFS, using the product-limit (Kaplan-Meier) method with a 95% Brookmeyer-Crowley credible region. Grade 3 and 4 AEs were tabulated by type.

### Study approval.

This study was approved by the University of Michigan and the University of Pittsburgh IRBs, and written consent was obtained from all patients prior to participation in the trial. No randomization or blinding was performed.

## Author contributions

LGC, RB, and KG conceived the project. LGC, DN, TJO, and LF curated data. LGC, TJO, DN, and LF provided formal analysis. LGC, DN, SU, KM, JB, MB, MCB, RPE, JL, HM, AO, PS, SET, RB, and KG provided investigation. LGC, RB, KG, and DN provided methodology. LGC, DN, SU, KM, JB, MB, MCB, RPE, JL, HM, AO, PS, SET, and RB provided supervision. LGC, TJO, TL, and DN wrote the original draft of the manuscript. LGC, TJO, TL, LF, DN, SU, KM, JB, MB, MCB, RPE, JL, HM, AO, PS, SET, and RB reviewed and edited the manuscript.

## Supplementary Material

ICMJE disclosure forms

## Figures and Tables

**Table 1:**
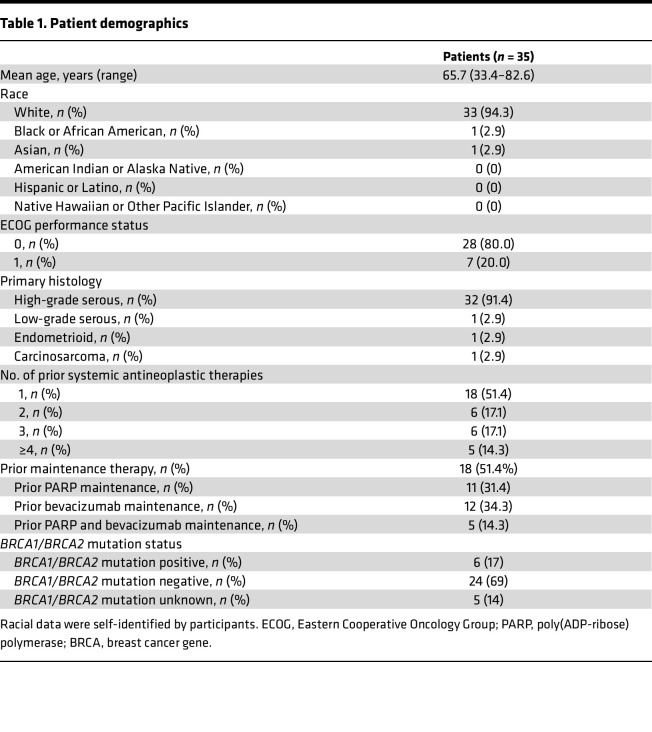
Patient demographics

**Table 2:**
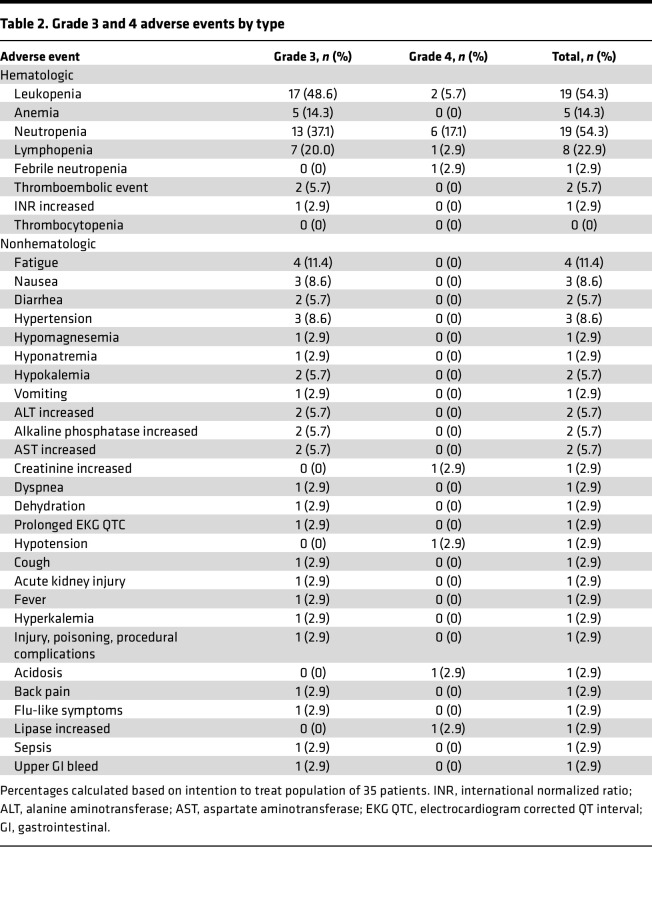
Grade 3 and 4 adverse events by type

**Table 3:**
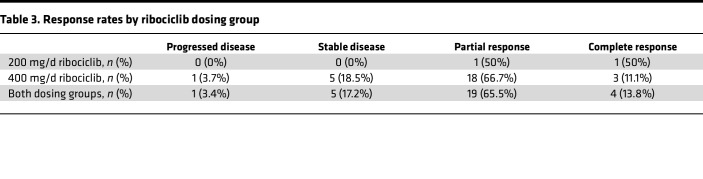
Response rates by ribociclib dosing group

## Version 1 08/16/2022

In-Press Preview

## Version 2 09/22/2022

Electronic publication
