# Supplementary material for: Phase I trial of ribociclib with platinum chemotherapy in ovarian cancer
Source: JCI Insight. 2022 Sep 22;7(18):e160573. doi: 10.1172/jci.insight.160573 (PMC9675476; doi:10.1172/jci.insight.160573)
Supplement: ICMJE disclosure forms [file jciinsight-7-160573-s098.pdf]

# ICMJE DISCLOSURE FORM

**Date:** 7/21/2022

**Your Name:** Lan Coffman

**Manuscript Title:** Phase I Trial of Ribociclib (LEE-011) with Platinum-based Chemotherapy in Recurrent Platinum Sensitive Ovarian Cancer

**Manuscript Number (if known):** 160573-INS-CMED-RV-2

In the interest of transparency, we ask you to disclose all relationships/activities/interests listed below that are related to the content of your manuscript. "Related" means any relation with for-profit or not-for-profit third parties whose interests may be affected by the content of the manuscript. Disclosure represents a commitment to transparency and does not necessarily indicate a bias. If you are in doubt about whether to list a relationship/activity/interest, it is preferable that you do so.

The author's relationships/activities/interests should be defined broadly. For example, if your manuscript pertains to the epidemiology of hypertension, you should declare all relationships with manufacturers of antihypertensive medication, even if that medication is not mentioned in the manuscript.

In item #1 below, report all support for the work reported in this manuscript without time limit. For all other items, the time frame for disclosure is the past 36 months.

| | | Name all entities with whom you have this relationship or indicate none (add rows as needed) | Specifications/Comments (e.g., if payments were made to you or to your institution) |
|---|---|---|---|
| **Time frame: Since the initial planning of the work** | | | |
| 1 | All support for the present manuscript (e.g., funding, provision of study materials, medical writing, article processing charges, etc.) **No time limit for this item.** | ☐ None | |
| | | Novartis provided funding for this investigator initiated trial | |
| | | | |
| | | | Click the tab key to add additional rows. |
| **Time frame: past 36 months** | | | |
| **2** | Grants or contracts from any entity (if not indicated in item #1 above). | ☒ **None** | |
| | | | |
| | | | |
| | | | |
| **3** | Royalties or licenses | ☒ **None** | |
| | | | |
| | | | |
| | | | |

| | | Name all entities with whom you have this relationship or indicate none (add rows as needed) | Specifications/Comments (e.g., if payments were made to you or to your institution) |
|---|---|---|---|
| **4** | Consulting fees | ☐ **None** | |
| | | Immunogen | One-time consulting relationship with a $600 fee |
| | | | |
| | | | |
| | | | |
| **5** | Payment or honoraria for lectures, presentations, speakers bureaus, manuscript writing or educational events | ☒ **None** | |
| | | | |
| | | | |
| | | | |
| **6** | Payment for expert testimony | ☒ **None** | |
| | | | |
| | | | |
| | | | |
| **7** | Support for attending meetings and/or travel | ☐ **None** | |
| | | Travel was paid for by the Tina's Wish foundation to attending the annual grant awardee meeting | |
| | | | |
| | | | |
| **8** | Patents planned, issued or pending | ☒ **None** | |
| | | | |
| | | | |
| | | | |
| **9** | Participation on a Data Safety Monitoring Board or Advisory Board | ☐ **None** | |
| | | Participated in an Advisory Board meeting for Immunogen | |
| | | | |
| | | | |
| **10** | Leadership or fiduciary role in other board, society, committee or advocacy group, paid or unpaid | ☒ **None** | |
| | | | |
| | | | |
| | | | |

| | | Name all entities with whom you have this relationship or indicate none (add rows as needed) | Specifications/Comments (e.g., if payments were made to you or to your institution) |
|---|---|---|---|
| **11** | Stock or stock options | ☒ **None** | |
| | | | |
| | | | |
| | | | |
| **12** | Receipt of equipment, materials, drugs, medical writing, gifts or other services | ☒ **None** | |
| | | | |
| | | | |
| | | | |
| **13** | Other financial or non-financial interests | ☒ **None** | |
| | | | |
| | | | |
| | | | |

**Please place an "X" next to the following statement to indicate your agreement:**

☒ I certify that I have answered every question and have not altered the wording of any of the questions on this form.

# ICMJE DISCLOSURE FORM

**Date:** 7/24/2022

**Your Name:** Taylor Orellana, MD

**Manuscript Title:** Phase I Trial of Ribociclib (LEE-011) with Platinum-based Chemotherapy in Recurrent Platinum Sensitive Ovarian Cancer

**Manuscript Number (if known):** 160573-INS-CMED-RV-2

In the interest of transparency, we ask you to disclose all relationships/activities/interests listed below that are related to the content of your manuscript. "Related" means any relation with for-profit or not-for-profit third parties whose interests may be affected by the content of the manuscript. Disclosure represents a commitment to transparency and does not necessarily indicate a bias. If you are in doubt about whether to list a relationship/activity/interest, it is preferable that you do so.

The author's relationships/activities/interests should be defined broadly. For example, if your manuscript pertains to the epidemiology of hypertension, you should declare all relationships with manufacturers of antihypertensive medication, even if that medication is not mentioned in the manuscript.

In item #1 below, report all support for the work reported in this manuscript without time limit. For all other items, the time frame for disclosure is the past 36 months.

| | | Name all entities with whom you have this relationship or indicate none (add rows as needed) | Specifications/Comments (e.g., if payments were made to you or to your institution) |
|---|---|---|---|
| | | **Time frame: Since the initial planning of the work** | |
| 1 | All support for the present manuscript (e.g., funding, provision of study materials, medical writing, article processing charges, etc.) **No time limit for this item.** | ☒ None | |
| | | | |
| | | | |
| | | | Click the tab key to add additional rows. |
| | | **Time frame: past 36 months** | |
| 2 | Grants or contracts from any entity (if not indicated in item #1 above). | ☒ **None** | |
| | | | |
| | | | |
| | | | |
| 3 | Royalties or licenses | ☒ **None** | |
| | | | |
| | | | |
| | | | |

| | | Name all entities with whom you have this relationship or indicate none (add rows as needed) | Specifications/Comments (e.g., if payments were made to you or to your institution) |
|---|---|---|---|
| **4** | Consulting fees | ☒ **None** | |
| | | | |
| | | | |
| | | | |
| | | | |
| **5** | Payment or honoraria for lectures, presentations, speakers bureaus, manuscript writing or educational events | ☒ **None** | |
| | | | |
| | | | |
| | | | |
| **6** | Payment for expert testimony | ☒ **None** | |
| | | | |
| | | | |
| | | | |
| **7** | Support for attending meetings and/or travel | ☒ **None** | |
| | | | |
| | | | |
| | | | |
| **8** | Patents planned, issued or pending | ☒ **None** | |
| | | | |
| | | | |
| | | | |
| **9** | Participation on a Data Safety Monitoring Board or Advisory Board | ☒ **None** | |
| | | | |
| | | | |
| | | | |
| **10** | Leadership or fiduciary role in other board, society, committee or advocacy group, paid or unpaid | ☒ **None** | |
| | | | |
| | | | |
| | | | |

| | | Name all entities with whom you have this relationship or indicate none (add rows as needed) | Specifications/Comments (e.g., if payments were made to you or to your institution) |
|---|---|---|---|
| **11** | Stock or stock options | ☒ **None** | |
| | | | |
| | | | |
| | | | |
| **12** | Receipt of equipment, materials, drugs, medical writing, gifts or other services | ☒ **None** | |
| | | | |
| | | | |
| | | | |
| **13** | Other financial or non-financial interests | ☒ **None** | |
| | | | |
| | | | |
| | | | |

**Please place an "X" next to the following statement to indicate your agreement:**

☒ I certify that I have answered every question and have not altered the wording of any of the questions on this form.

# ICMJE DISCLOSURE FORM

**Date:** 7/23/2022

**Your Name:** Tianshi Liu

**Manuscript Title:** Phase I Trial of Ribociclib (LEE-011) with Platinum-based Chemotherapy in Recurrent Platinum Sensitive Ovarian Cancer

**Manuscript Number (if known):** 160573-INS-CMED-RV-2

In the interest of transparency, we ask you to disclose all relationships/activities/interests listed below that are related to the content of your manuscript. "Related" means any relation with for-profit or not-for-profit third parties whose interests may be affected by the content of the manuscript. Disclosure represents a commitment to transparency and does not necessarily indicate a bias. If you are in doubt about whether to list a relationship/activity/interest, it is preferable that you do so.

The author's relationships/activities/interests should be defined broadly. For example, if your manuscript pertains to the epidemiology of hypertension, you should declare all relationships with manufacturers of antihypertensive medication, even if that medication is not mentioned in the manuscript.

In item #1 below, report all support for the work reported in this manuscript without time limit. For all other items, the time frame for disclosure is the past 36 months.

| | | **Name all entities with whom you have this relationship or indicate none (add rows as needed)** | **Specifications/Comments (e.g., if payments were made to you or to your institution)** |
|---|---|---|---|
| | | **Time frame: Since the initial planning of the work** | |
| 1 | All support for the present manuscript (e.g., funding, provision of study materials, medical writing, article processing charges, etc.) **No time limit for this item.** | ☒ None | |
| | | | |
| | | | |
| | | | Click the tab key to add additional rows. |
| | | **Time frame: past 36 months** | |
| **2** | Grants or contracts from any entity (if not indicated in item #1 above). | ☒ **None** | |
| | | | |
| | | | |
| | | | |
| **3** | Royalties or licenses | ☒ **None** | |
| | | | |
| | | | |
| | | | |

| | | Name all entities with whom you have this relationship or indicate none (add rows as needed) | Specifications/Comments (e.g., if payments were made to you or to your institution) |
|---|---|---|---|
| **4** | Consulting fees | ☒ **None** | |
| | | | |
| | | | |
| | | | |
| | | | |
| **5** | Payment or honoraria for lectures, presentations, speakers bureaus, manuscript writing or educational events | ☒ **None** | |
| | | | |
| | | | |
| | | | |
| **6** | Payment for expert testimony | ☒ **None** | |
| | | | |
| | | | |
| | | | |
| **7** | Support for attending meetings and/or travel | ☒ **None** | |
| | | | |
| | | | |
| | | | |
| **8** | Patents planned, issued or pending | ☒ **None** | |
| | | | |
| | | | |
| | | | |
| **9** | Participation on a Data Safety Monitoring Board or Advisory Board | ☒ **None** | |
| | | | |
| | | | |
| | | | |
| **10** | Leadership or fiduciary role in other board, society, committee or advocacy group, paid or unpaid | ☒ **None** | |
| | | | |
| | | | |
| | | | |

| | | Name all entities with whom you have this relationship or indicate none (add rows as needed) | Specifications/Comments (e.g., if payments were made to you or to your institution) |
|---|---|---|---|
| **11** | Stock or stock options | ☒ **None** | |
| | | | |
| | | | |
| | | | |
| **12** | Receipt of equipment, materials, drugs, medical writing, gifts or other services | ☒ **None** | |
| | | | |
| | | | |
| | | | |
| **13** | Other financial or non-financial interests | ☒ **None** | |
| | | | |
| | | | |
| | | | |

**Please place an "X" next to the following statement to indicate your agreement:**

☒ I certify that I have answered every question and have not altered the wording of any of the questions on this form.

# ICMJE DISCLOSURE FORM

| | |
|---|---|
| **Date:** | 7/23/2022 |
| **Your Name:** | Daniel Normolle |
| **Manuscript Title:** | Phase I Trial of Ribociclib (LEE-011) with Platinum-based Chemotherapy in Recurrent Platinum Sensitive Ovarian Cancer |
| **Manuscript Number (if known):** | 160573-INS-CMED-RV-2 |

In the interest of transparency, we ask you to disclose all relationships/activities/interests listed below that are related to the content of your manuscript. "Related" means any relation with for-profit or not-for-profit third parties whose interests may be affected by the content of the manuscript. Disclosure represents a commitment to transparency and does not necessarily indicate a bias. If you are in doubt about whether to list a relationship/activity/interest, it is preferable that you do so.

The author's relationships/activities/interests should be defined broadly. For example, if your manuscript pertains to the epidemiology of hypertension, you should declare all relationships with manufacturers of antihypertensive medication, even if that medication is not mentioned in the manuscript.

In item #1 below, report all support for the work reported in this manuscript without time limit. For all other items, the time frame for disclosure is the past 36 months.

| | | **Name all entities with whom you have this relationship or indicate none (add rows as needed)** | **Specifications/Comments (e.g., if payments were made to you or to your institution)** |
|---|---|---|---|
| | | Time frame: Since the initial planning of the work | |
| 1 | All support for the present manuscript (e.g., funding, provision of study materials, medical writing, article processing charges, etc.) **No time limit for this item.** | ☒ None | |
| | | | |
| | | | |
| | | | Click the tab key to add additional rows. |
| | | Time frame: past 36 months | |
| **2** | Grants or contracts from any entity (if not indicated in item #1 above). | ☒ **None** | |
| | | | |
| | | | |
| | | | |
| **3** | Royalties or licenses | ☒ **None** | |
| | | | |
| | | | |
| | | | |

| | | Name all entities with whom you have this relationship or indicate none (add rows as needed) | Specifications/Comments (e.g., if payments were made to you or to your institution) |
|---|---|---|---|
| **4** | Consulting fees | ☒ **None** | |
| | | | |
| | | | |
| | | | |
| | | | |
| **5** | Payment or honoraria for lectures, presentations, speakers bureaus, manuscript writing or educational events | ☒ **None** | |
| | | | |
| | | | |
| | | | |
| **6** | Payment for expert testimony | ☒ **None** | |
| | | | |
| | | | |
| | | | |
| **7** | Support for attending meetings and/or travel | ☒ **None** | |
| | | | |
| | | | |
| | | | |
| **8** | Patents planned, issued or pending | ☒ **None** | |
| | | | |
| | | | |
| | | | |
| **9** | Participation on a Data Safety Monitoring Board or Advisory Board | ☒ **None** | |
| | | | |
| | | | |
| | | | |
| **10** | Leadership or fiduciary role in other board, society, committee or advocacy group, paid or unpaid | ☒ **None** | |
| | | | |
| | | | |
| | | | |

| | | Name all entities with whom you have this relationship or indicate none (add rows as needed) | Specifications/Comments (e.g., if payments were made to you or to your institution) |
|---|---|---|---|
| **11** | Stock or stock options | ☒ **None** | |
| | | | |
| | | | |
| | | | |
| **12** | Receipt of equipment, materials, drugs, medical writing, gifts or other services | ☒ **None** | |
| | | | |
| | | | |
| | | | |
| **13** | Other financial or non-financial interests | ☒ **None** | |
| | | | |
| | | | |
| | | | |

**Please place an "X" next to the following statement to indicate your agreement:**

☒ I certify that I have answered every question and have not altered the wording of any of the questions on this form.

# ICMJE DISCLOSURE FORM

| | |
|---|---|
| **Date:** | 7/24/2022 |
| **Your Name:** | Kent Griffith, PhD |
| **Manuscript Title:** | Phase I Trial of Ribociclib (LEE-011) with Platinum-based Chemotherapy in Recurrent Platinum Sensitive Ovarian Cancer |
| **Manuscript Number (if known):** | 160573-INS-CMED-RV-2 |

In the interest of transparency, we ask you to disclose all relationships/activities/interests listed below that are related to the content of your manuscript. "Related" means any relation with for-profit or not-for-profit third parties whose interests may be affected by the content of the manuscript. Disclosure represents a commitment to transparency and does not necessarily indicate a bias. If you are in doubt about whether to list a relationship/activity/interest, it is preferable that you do so.

The author's relationships/activities/interests should be defined broadly. For example, if your manuscript pertains to the epidemiology of hypertension, you should declare all relationships with manufacturers of antihypertensive medication, even if that medication is not mentioned in the manuscript.

In item #1 below, report all support for the work reported in this manuscript without time limit. For all other items, the time frame for disclosure is the past 36 months.

| | | **Name all entities with whom you have this relationship or indicate none (add rows as needed)** | **Specifications/Comments (e.g., if payments were made to you or to your institution)** |
|---|---|---|---|
| **Time frame: Since the initial planning of the work** | | | |
| 1 | All support for the present manuscript (e.g., funding, provision of study materials, medical writing, article processing charges, etc.) **No time limit for this item.** | ☒ None | |
| | | | |
| | | | |
| | | | Click the tab key to add additional rows. |
| **Time frame: past 36 months** | | | |
| **2** | Grants or contracts from any entity (if not indicated in item #1 above). | ☒ **None** | |
| | | | |
| | | | |
| | | | |
| **3** | Royalties or licenses | ☒ **None** | |
| | | | |
| | | | |
| | | | |

| | | Name all entities with whom you have this relationship or indicate none (add rows as needed) | Specifications/Comments (e.g., if payments were made to you or to your institution) |
|---|---|---|---|
| **4** | Consulting fees | ☒ **None** | |
| | | | |
| | | | |
| | | | |
| | | | |
| **5** | Payment or honoraria for lectures, presentations, speakers bureaus, manuscript writing or educational events | ☒ **None** | |
| | | | |
| | | | |
| | | | |
| **6** | Payment for expert testimony | ☒ **None** | |
| | | | |
| | | | |
| | | | |
| **7** | Support for attending meetings and/or travel | ☒ **None** | |
| | | | |
| | | | |
| | | | |
| **8** | Patents planned, issued or pending | ☒ **None** | |
| | | | |
| | | | |
| | | | |
| **9** | Participation on a Data Safety Monitoring Board or Advisory Board | ☒ **None** | |
| | | | |
| | | | |
| | | | |
| **10** | Leadership or fiduciary role in other board, society, committee or advocacy group, paid or unpaid | ☒ **None** | |
| | | | |
| | | | |
| | | | |

| | | Name all entities with whom you have this relationship or indicate none (add rows as needed) | Specifications/Comments (e.g., if payments were made to you or to your institution) |
|---|---|---|---|
| **11** | Stock or stock options | ☒ **None** | |
| | | | |
| | | | |
| | | | |
| **12** | Receipt of equipment, materials, drugs, medical writing, gifts or other services | ☒ **None** | |
| | | | |
| | | | |
| | | | |
| **13** | Other financial or non-financial interests | ☒ **None** | |
| | | | |
| | | | |
| | | | |

**Please place an "X" next to the following statement to indicate your agreement:**

☒ I certify that I have answered every question and have not altered the wording of any of the questions on this form.

# ICMJE DISCLOSURE FORM

**Date:** 7/21/2022

**Your Name:** Shitanshu Uppal

**Manuscript Title:** Phase I Trial of Ribociclib (LEE-011) with Platinum-based Chemotherapy in Recurrent Platinum Sensitive Ovarian Cancer

**Manuscript Number (if known):** 160573-INS-CMED-RV-2

In the interest of transparency, we ask you to disclose all relationships/activities/interests listed below that are related to the content of your manuscript. "Related" means any relation with for-profit or not-for-profit third parties whose interests may be affected by the content of the manuscript. Disclosure represents a commitment to transparency and does not necessarily indicate a bias. If you are in doubt about whether to list a relationship/activity/interest, it is preferable that you do so.

The author's relationships/activities/interests should be defined broadly. For example, if your manuscript pertains to the epidemiology of hypertension, you should declare all relationships with manufacturers of antihypertensive medication, even if that medication is not mentioned in the manuscript.

In item #1 below, report all support for the work reported in this manuscript without time limit. For all other items, the time frame for disclosure is the past 36 months.

| | | **Name all entities with whom you have this relationship or indicate none (add rows as needed)** | **Specifications/Comments (e.g., if payments were made to you or to your institution)** |
|---|---|---|---|
| | | Time frame: Since the initial planning of the work | |
| 1 | All support for the present manuscript (e.g., funding, provision of study materials, medical writing, article processing charges, etc.) **No time limit for this item.** | ☒ None | |
| | | | |
| | | | |
| | | | Click the tab key to add additional rows. |
| | | Time frame: past 36 months | |
| **2** | Grants or contracts from any entity (if not indicated in item #1 above). | ☒ **None** | |
| | | | |
| | | | |
| | | | |
| **3** | Royalties or licenses | ☒ **None** | |
| | | | |
| | | | |
| | | | |

| | | Name all entities with whom you have this relationship or indicate none (add rows as needed) | Specifications/Comments (e.g., if payments were made to you or to your institution) |
|---|---|---|---|
| **4** | Consulting fees | ☒ **None** | |
| | | | |
| | | | |
| | | | |
| | | | |
| **5** | Payment or honoraria for lectures, presentations, speakers bureaus, manuscript writing or educational events | ☒ **None** | |
| | | | |
| | | | |
| | | | |
| **6** | Payment for expert testimony | ☒ **None** | |
| | | | |
| | | | |
| | | | |
| **7** | Support for attending meetings and/or travel | ☒ **None** | |
| | | | |
| | | | |
| | | | |
| **8** | Patents planned, issued or pending | ☒ **None** | |
| | | | |
| | | | |
| | | | |
| **9** | Participation on a Data Safety Monitoring Board or Advisory Board | ☒ **None** | |
| | | | |
| | | | |
| | | | |
| **10** | Leadership or fiduciary role in other board, society, committee or advocacy group, paid or unpaid | ☒ **None** | |
| | | | |
| | | | |
| | | | |

| | | Name all entities with whom you have this relationship or indicate none (add rows as needed) | Specifications/Comments (e.g., if payments were made to you or to your institution) |
|---|---|---|---|
| **11** | Stock or stock options | ☒ **None** | |
| | | | |
| | | | |
| | | | |
| **12** | Receipt of equipment, materials, drugs, medical writing, gifts or other services | ☒ **None** | |
| | | | |
| | | | |
| | | | |
| **13** | Other financial or non-financial interests | ☒ **None** | |
| | | | |
| | | | |
| | | | |

**Please place an "X" next to the following statement to indicate your agreement:**

☒ I certify that I have answered every question and have not altered the wording of any of the questions on this form.

Shitanshu Uppal

7/21/22

# ICMJE DISCLOSURE FORM

| | |
|---|---|
| **Date:** | 7/22/2022 |
| **Your Name:** | Karen McLean, MD, PhD |
| **Manuscript Title:** | Phase I Trial of Ribociclib (LEE-011) with Platinum-based Chemotherapy in Recurrent Platinum Sensitive Ovarian Cancer |
| **Manuscript Number (if known):** | 160573-INS-CMED-RV-2 |

In the interest of transparency, we ask you to disclose all relationships/activities/interests listed below that are related to the content of your manuscript. "Related" means any relation with for-profit or not-for-profit third parties whose interests may be affected by the content of the manuscript. Disclosure represents a commitment to transparency and does not necessarily indicate a bias. If you are in doubt about whether to list a relationship/activity/interest, it is preferable that you do so.

The author's relationships/activities/interests should be defined broadly. For example, if your manuscript pertains to the epidemiology of hypertension, you should declare all relationships with manufacturers of antihypertensive medication, even if that medication is not mentioned in the manuscript.

In item #1 below, report all support for the work reported in this manuscript without time limit. For all other items, the time frame for disclosure is the past 36 months.

| | | **Name all entities with whom you have this relationship or indicate none (add rows as needed)** | **Specifications/Comments (e.g., if payments were made to you or to your institution)** |
|---|---|---|---|
| Time frame: Since the initial planning of the work | | | |
| 1 | All support for the present manuscript (e.g., funding, provision of study materials, medical writing, article processing charges, etc.) **No time limit for this item.** | ☒ None | |
| | | | |
| | | | |
| | | | Click the tab key to add additional rows. |
| Time frame: past 36 months | | | |
| 2 | Grants or contracts from any entity (if not indicated in item #1 above). | ☒ **None** | |
| | | | |
| | | | |
| | | | |
| 3 | Royalties or licenses | ☒ **None** | |
| | | | |
| | | | |
| | | | |

| | | Name all entities with whom you have this relationship or indicate none (add rows as needed) | Specifications/Comments (e.g., if payments were made to you or to your institution) |
|---|---|---|---|
| **4** | Consulting fees | ☒ **None** | |
| | | | |
| | | | |
| | | | |
| | | | |
| **5** | Payment or honoraria for lectures, presentations, speakers bureaus, manuscript writing or educational events | ☒ **None** | |
| | | | |
| | | | |
| | | | |
| **6** | Payment for expert testimony | ☒ **None** | |
| | | | |
| | | | |
| | | | |
| **7** | Support for attending meetings and/or travel | ☒ **None** | |
| | | | |
| | | | |
| | | | |
| **8** | Patents planned, issued or pending | ☒ **None** | |
| | | | |
| | | | |
| | | | |
| **9** | Participation on a Data Safety Monitoring Board or Advisory Board | ☒ **None** | |
| | | | |
| | | | |
| | | | |
| **10** | Leadership or fiduciary role in other board, society, committee or advocacy group, paid or unpaid | ☒ **None** | |
| | | | |
| | | | |
| | | | |

| | | Name all entities with whom you have this relationship or indicate none (add rows as needed) | Specifications/Comments (e.g., if payments were made to you or to your institution) |
|---|---|---|---|
| **11** | Stock or stock options | ☒ **None** | |
| | | | |
| | | | |
| | | | |
| **12** | Receipt of equipment, materials, drugs, medical writing, gifts or other services | ☒ **None** | |
| | | | |
| | | | |
| | | | |
| **13** | Other financial or non-financial interests | ☒ **None** | |
| | | | |
| | | | |
| | | | |

**Please place an "X" next to the following statement to indicate your agreement:**

☒ I certify that I have answered every question and have not altered the wording of any of the questions on this form.

# ICMJE DISCLOSURE FORM

**Date:** 7/24/2022

**Your Name:** Jessica Berger, MD

**Manuscript Title:** Phase I Trial of Ribociclib (LEE-011) with Platinum-based Chemotherapy in Recurrent Platinum Sensitive Ovarian Cancer

**Manuscript Number (if known):** 160573-INS-CMED-RV-2

In the interest of transparency, we ask you to disclose all relationships/activities/interests listed below that are related to the content of your manuscript. "Related" means any relation with for-profit or not-for-profit third parties whose interests may be affected by the content of the manuscript. Disclosure represents a commitment to transparency and does not necessarily indicate a bias. If you are in doubt about whether to list a relationship/activity/interest, it is preferable that you do so.

The author's relationships/activities/interests should be defined broadly. For example, if your manuscript pertains to the epidemiology of hypertension, you should declare all relationships with manufacturers of antihypertensive medication, even if that medication is not mentioned in the manuscript.

In item #1 below, report all support for the work reported in this manuscript without time limit. For all other items, the time frame for disclosure is the past 36 months.

| | | **Name all entities with whom you have this relationship or indicate none (add rows as needed)** | **Specifications/Comments (e.g., if payments were made to you or to your institution)** |
|---|---|---|---|
| **Time frame: Since the initial planning of the work** | | | |
| 1 | All support for the present manuscript (e.g., funding, provision of study materials, medical writing, article processing charges, etc.) **No time limit for this item.** | ☒ None | |
| | | | |
| | | | |
| | | | Click the tab key to add additional rows. |
| **Time frame: past 36 months** | | | |
| 2 | Grants or contracts from any entity (if not indicated in item #1 above). | ☒ **None** | |
| | | | |
| | | | |
| | | | |
| 3 | Royalties or licenses | ☒ **None** | |
| | | | |
| | | | |
| | | | |

| | | Name all entities with whom you have this relationship or indicate none (add rows as needed) | Specifications/Comments (e.g., if payments were made to you or to your institution) |
|---|---|---|---|
| 4 | Consulting fees | ☒ None | |
| | | | |
| | | | |
| | | | |
| | | | |
| 5 | Payment or honoraria for lectures, presentations, speakers bureaus, manuscript writing or educational events | ☒ None | |
| | | | |
| | | | |
| | | | |
| 6 | Payment for expert testimony | ☒ None | |
| | | | |
| | | | |
| | | | |
| 7 | Support for attending meetings and/or travel | ☒ None | |
| | | | |
| | | | |
| | | | |
| 8 | Patents planned, issued or pending | ☒ None | |
| | | | |
| | | | |
| | | | |
| 9 | Participation on a Data Safety Monitoring Board or Advisory Board | ☒ None | |
| | | | |
| | | | |
| | | | |
| 10 | Leadership or fiduciary role in other board, society, committee or advocacy group, paid or unpaid | ☒ None | |
| | | | |
| | | | |
| | | | |

| | | Name all entities with whom you have this relationship or indicate none (add rows as needed) | Specifications/Comments (e.g., if payments were made to you or to your institution) |
|---|---|---|---|
| **11** | Stock or stock options | ☒ **None** | |
| | | | |
| | | | |
| | | | |
| **12** | Receipt of equipment, materials, drugs, medical writing, gifts or other services | ☒ **None** | |
| | | | |
| | | | |
| | | | |
| **13** | Other financial or non-financial interests | ☒ **None** | |
| | | | |
| | | | |
| | | | |

**Please place an "X" next to the following statement to indicate your agreement:**

☒ I certify that I have answered every question and have not altered the wording of any of the questions on this form.

# ICMJE DISCLOSURE FORM

**Date:** 7/24/2022

**Your Name:** Michelle Boisen, MD

**Manuscript Title:** Phase I Trial of Ribociclib (LEE-011) with Platinum-based Chemotherapy in Recurrent Platinum Sensitive Ovarian Cancer

**Manuscript Number (if known):** 160573-INS-CMED-RV-2

In the interest of transparency, we ask you to disclose all relationships/activities/interests listed below that are related to the content of your manuscript. "Related" means any relation with for-profit or not-for-profit third parties whose interests may be affected by the content of the manuscript. Disclosure represents a commitment to transparency and does not necessarily indicate a bias. If you are in doubt about whether to list a relationship/activity/interest, it is preferable that you do so.

The author's relationships/activities/interests should be defined broadly. For example, if your manuscript pertains to the epidemiology of hypertension, you should declare all relationships with manufacturers of antihypertensive medication, even if that medication is not mentioned in the manuscript.

In item #1 below, report all support for the work reported in this manuscript without time limit. For all other items, the time frame for disclosure is the past 36 months.

| | | Name all entities with whom you have this relationship or indicate none (add rows as needed) | Specifications/Comments (e.g., if payments were made to you or to your institution) |
|---|---|---|---|
| **Time frame: Since the initial planning of the work** | | | |
| 1 | All support for the present manuscript (e.g., funding, provision of study materials, medical writing, article processing charges, etc.) **No time limit for this item.** | ☒ None | |
| | | | |
| | | | |
| | | | Click the tab key to add additional rows. |
| **Time frame: past 36 months** | | | |
| **2** | Grants or contracts from any entity (if not indicated in item #1 above). | ☒ **None** | |
| | | | |
| | | | |
| | | | |
| **3** | Royalties or licenses | ☒ **None** | |
| | | | |
| | | | |
| | | | |

| | | Name all entities with whom you have this relationship or indicate none (add rows as needed) | Specifications/Comments (e.g., if payments were made to you or to your institution) |
|---|---|---|---|
| **4** | Consulting fees | ☒ **None** | |
| | | | |
| | | | |
| | | | |
| | | | |
| **5** | Payment or honoraria for lectures, presentations, speakers bureaus, manuscript writing or educational events | ☒ **None** | |
| | | | |
| | | | |
| | | | |
| **6** | Payment for expert testimony | ☒ **None** | |
| | | | |
| | | | |
| | | | |
| **7** | Support for attending meetings and/or travel | ☒ **None** | |
| | | | |
| | | | |
| | | | |
| **8** | Patents planned, issued or pending | ☒ **None** | |
| | | | |
| | | | |
| | | | |
| **9** | Participation on a Data Safety Monitoring Board or Advisory Board | ☒ **None** | |
| | | | |
| | | | |
| | | | |
| **10** | Leadership or fiduciary role in other board, society, committee or advocacy group, paid or unpaid | ☒ **None** | |
| | | | |
| | | | |
| | | | |

| | | Name all entities with whom you have this relationship or indicate none (add rows as needed) | Specifications/Comments (e.g., if payments were made to you or to your institution) |
|---|---|---|---|
| **11** | Stock or stock options | ☒ **None** | |
| | | | |
| | | | |
| | | | |
| **12** | Receipt of equipment, materials, drugs, medical writing, gifts or other services | ☒ **None** | |
| | | | |
| | | | |
| | | | |
| **13** | Other financial or non-financial interests | ☒ **None** | |
| | | | |
| | | | |
| | | | |

**Please place an "X" next to the following statement to indicate your agreement:**

☒ I certify that I have answered every question and have not altered the wording of any of the questions on this form.

# ICMJE DISCLOSURE FORM

**Date:** 7/22/2022

**Your Name:** Madeleine Courtney-Brooks

**Manuscript Title:** Phase I Trial of Ribociclib (LEE-011) with Platinum-based Chemotherapy in Recurrent Platinum Sensitive Ovarian Cancer

**Manuscript Number (if known):** 160573-INS-CMED-RV-2

In the interest of transparency, we ask you to disclose all relationships/activities/interests listed below that are related to the content of your manuscript. "Related" means any relation with for-profit or not-for-profit third parties whose interests may be affected by the content of the manuscript. Disclosure represents a commitment to transparency and does not necessarily indicate a bias. If you are in doubt about whether to list a relationship/activity/interest, it is preferable that you do so.

The author's relationships/activities/interests should be defined broadly. For example, if your manuscript pertains to the epidemiology of hypertension, you should declare all relationships with manufacturers of antihypertensive medication, even if that medication is not mentioned in the manuscript.

In item #1 below, report all support for the work reported in this manuscript without time limit. For all other items, the time frame for disclosure is the past 36 months.

| | | **Name all entities with whom you have this relationship or indicate none (add rows as needed)** | **Specifications/Comments (e.g., if payments were made to you or to your institution)** |
|---|---|---|---|
| **Time frame: Since the initial planning of the work** | | | |
| 1 | All support for the present manuscript (e.g., funding, provision of study materials, medical writing, article processing charges, etc.) **No time limit for this item.** | ☒ None | |
| | | | |
| | | | |
| | | | Click the tab key to add additional rows. |
| **Time frame: past 36 months** | | | |
| **2** | Grants or contracts from any entity (if not indicated in item #1 above). | ☒ **None** | |
| | | | |
| | | | |
| | | | |
| **3** | Royalties or licenses | ☒ **None** | |
| | | | |
| | | | |
| | | | |

| | | Name all entities with whom you have this relationship or indicate none (add rows as needed) | Specifications/Comments (e.g., if payments were made to you or to your institution) |
|---|---|---|---|
| 4 | Consulting fees | ☒ **None** | |
| | | | |
| | | | |
| | | | |
| | | | |
| 5 | Payment or honoraria for lectures, presentations, speakers bureaus, manuscript writing or educational events | ☒ **None** | |
| | | | |
| | | | |
| | | | |
| 6 | Payment for expert testimony | ☒ **None** | |
| | | | |
| | | | |
| | | | |
| 7 | Support for attending meetings and/or travel | ☒ **None** | |
| | | | |
| | | | |
| | | | |
| 8 | Patents planned, issued or pending | ☒ **None** | |
| | | | |
| | | | |
| | | | |
| 9 | Participation on a Data Safety Monitoring Board or Advisory Board | ☒ **None** | |
| | | | |
| | | | |
| | | | |
| 10 | Leadership or fiduciary role in other board, society, committee or advocacy group, paid or unpaid | ☒ **None** | |
| | | | |
| | | | |
| | | | |

| | | Name all entities with whom you have this relationship or indicate none (add rows as needed) | Specifications/Comments (e.g., if payments were made to you or to your institution) |
|---|---|---|---|
| **11** | Stock or stock options | ☒ **None** | |
| | | | |
| | | | |
| | | | |
| **12** | Receipt of equipment, materials, drugs, medical writing, gifts or other services | ☒ **None** | |
| | | | |
| | | | |
| | | | |
| **13** | Other financial or non-financial interests | ☒ **None** | |
| | | | |
| | | | |
| | | | |

**Please place an "X" next to the following statement to indicate your agreement:**

☒ I certify that I have answered every question and have not altered the wording of any of the questions on this form.

# ICMJE DISCLOSURE FORM

| | |
|---|---|
| **Date:** | 7/24/2022 |
| **Your Name:** | Robert P. Edwards |
| **Manuscript Title:** | Phase I Trial of Ribociclib (LEE-011) with Platinum-based Chemotherapy in Recurrent Platinum Sensitive Ovarian Cancer |
| **Manuscript Number (if known):** | 160573-INS-CMED-RV-2 |

In the interest of transparency, we ask you to disclose all relationships/activities/interests listed below that are related to the content of your manuscript. "Related" means any relation with for-profit or not-for-profit third parties whose interests may be affected by the content of the manuscript. Disclosure represents a commitment to transparency and does not necessarily indicate a bias. If you are in doubt about whether to list a relationship/activity/interest, it is preferable that you do so.

The author's relationships/activities/interests should be defined broadly. For example, if your manuscript pertains to the epidemiology of hypertension, you should declare all relationships with manufacturers of antihypertensive medication, even if that medication is not mentioned in the manuscript.

In item #1 below, report all support for the work reported in this manuscript without time limit. For all other items, the time frame for disclosure is the past 36 months.

| | | **Name all entities with whom you have this relationship or indicate none (add rows as needed)** | **Specifications/Comments (e.g., if payments were made to you or to your institution)** |
|---|---|---|---|
| | | Time frame: Since the initial planning of the work | |
| 1 | All support for the present manuscript (e.g., funding, provision of study materials, medical writing, article processing charges, etc.) **No time limit for this item.** | ☐ None | |
| | | Contributed study patients. | |
| | | | |
| | | | Click the tab key to add additional rows. |
| | | Time frame: past 36 months | |
| **2** | Grants or contracts from any entity (if not indicated in item #1 above). | ☒ **None** | |
| | | | |
| | | | |
| | | | |
| **3** | Royalties or licenses | ☒ **None** | |
| | | | |
| | | | |
| | | | |

| | | Name all entities with whom you have this relationship or indicate none (add rows as needed) | Specifications/Comments (e.g., if payments were made to you or to your institution) |
|---|---|---|---|
| **4** | Consulting fees | ☐ **None** | |
| | | Merck | DSMB for two international trials |
| | | | |
| | | | |
| | | | |
| **5** | Payment or honoraria for lectures, presentations, speakers bureaus, manuscript writing or educational events | ☒ **None** | |
| | | | |
| | | | |
| | | | |
| **6** | Payment for expert testimony | ☒ **None** | |
| | | | |
| | | | |
| | | | |
| **7** | Support for attending meetings and/or travel | ☒ **None** | |
| | | | |
| | | | |
| | | | |
| **8** | Patents planned, issued or pending | ☐ **None** | |
| | | Cell line patent pending. U of Pitt | |
| | | | |
| | | | |
| **9** | Participation on a Data Safety Monitoring Board or Advisory Board | ☐ **None** | |
| | | Merck Phase III trials | Two different trials |
| | | | |
| | | | |
| **10** | Leadership or fiduciary role in other board, society, committee or advocacy group, paid or unpaid | ☒ **None** | |
| | | | |
| | | | |
| | | | |

| | | Name all entities with whom you have this relationship or indicate none (add rows as needed) | Specifications/Comments (e.g., if payments were made to you or to your institution) |
|---|---|---|---|
| **11** | Stock or stock options | ☒ **None** | |
| | | | |
| | | | |
| | | | |
| **12** | Receipt of equipment, materials, drugs, medical writing, gifts or other services | ☒ **None** | |
| | | | |
| | | | |
| | | | |
| **13** | Other financial or non-financial interests | ☒ **None** | |
| | | | |
| | | | |
| | | | |

**Please place an "X" next to the following statement to indicate your agreement:**

☒ I certify that I have answered every question and have not altered the wording of any of the questions on this form.

# ICMJE DISCLOSURE FORM

| | |
|---|---|
| **Date:** | 7/22/2022 |
| **Your Name:** | Jamie Lesnock, MD |
| **Manuscript Title:** | Phase I Trial of Ribociclib (LEE-011) with Platinum-based Chemotherapy in Recurrent Platinum Sensitive Ovarian Cancer |
| **Manuscript Number (if known):** | 160573-INS-CMED-RV-2 |

In the interest of transparency, we ask you to disclose all relationships/activities/interests listed below that are related to the content of your manuscript. "Related" means any relation with for-profit or not-for-profit third parties whose interests may be affected by the content of the manuscript. Disclosure represents a commitment to transparency and does not necessarily indicate a bias. If you are in doubt about whether to list a relationship/activity/interest, it is preferable that you do so.

The author's relationships/activities/interests should be defined broadly. For example, if your manuscript pertains to the epidemiology of hypertension, you should declare all relationships with manufacturers of antihypertensive medication, even if that medication is not mentioned in the manuscript.

In item #1 below, report all support for the work reported in this manuscript without time limit. For all other items, the time frame for disclosure is the past 36 months.

| | | **Name all entities with whom you have this relationship or indicate none (add rows as needed)** | **Specifications/Comments (e.g., if payments were made to you or to your institution)** |
|---|---|---|---|
| **Time frame: Since the initial planning of the work** | | | |
| 1 | All support for the present manuscript (e.g., funding, provision of study materials, medical writing, article processing charges, etc.) **No time limit for this item.** | ☒ None | |
| | | | |
| | | | |
| | | | Click the tab key to add additional rows. |
| **Time frame: past 36 months** | | | |
| **2** | Grants or contracts from any entity (if not indicated in item #1 above). | ☒ **None** | |
| | | | |
| | | | |
| | | | |
| **3** | Royalties or licenses | ☒ **None** | |
| | | | |
| | | | |
| | | | |

| | | Name all entities with whom you have this relationship or indicate none (add rows as needed) | Specifications/Comments (e.g., if payments were made to you or to your institution) |
|---|---|---|---|
| **4** | Consulting fees | ☒ **None** | |
| | | | |
| | | | |
| | | | |
| | | | |
| **5** | Payment or honoraria for lectures, presentations, speakers bureaus, manuscript writing or educational events | ☒ **None** | |
| | | | |
| | | | |
| **6** | Payment for expert testimony | ☒ **None** | |
| | | | |
| | | | |
| | | | |
| **7** | Support for attending meetings and/or travel | ☒ **None** | |
| | | | |
| | | | |
| | | | |
| **8** | Patents planned, issued or pending | ☒ **None** | |
| | | | |
| | | | |
| | | | |
| **9** | Participation on a Data Safety Monitoring Board or Advisory Board | ☒ **None** | |
| | | | |
| | | | |
| | | | |
| **10** | Leadership or fiduciary role in other board, society, committee or advocacy group, paid or unpaid | ☒ **None** | |
| | | | |
| | | | |
| | | | |

| | | Name all entities with whom you have this relationship or indicate none (add rows as needed) | Specifications/Comments (e.g., if payments were made to you or to your institution) |
|---|---|---|---|
| **11** | Stock or stock options | ☒ **None** | |
| | | | |
| | | | |
| | | | |
| **12** | Receipt of equipment, materials, drugs, medical writing, gifts or other services | ☒ **None** | |
| | | | |
| | | | |
| | | | |
| **13** | Other financial or non-financial interests | ☒ **None** | |
| | | | |
| | | | |
| | | | |

**Please place an "X" next to the following statement to indicate your agreement:**

☒ I certify that I have answered every question and have not altered the wording of any of the questions on this form.

# ICMJE DISCLOSURE FORM

**Date:** 7/24/2022

**Your Name:** Haider Mahdi, MD

**Manuscript Title:** Phase I Trial of Ribociclib (LEE-011) with Platinum-based Chemotherapy in Recurrent Platinum Sensitive Ovarian Cancer

**Manuscript Number (if known):** 160573-INS-CMED-RV-2

In the interest of transparency, we ask you to disclose all relationships/activities/interests listed below that are related to the content of your manuscript. "Related" means any relation with for-profit or not-for-profit third parties whose interests may be affected by the content of the manuscript. Disclosure represents a commitment to transparency and does not necessarily indicate a bias. If you are in doubt about whether to list a relationship/activity/interest, it is preferable that you do so.

The author's relationships/activities/interests should be defined broadly. For example, if your manuscript pertains to the epidemiology of hypertension, you should declare all relationships with manufacturers of antihypertensive medication, even if that medication is not mentioned in the manuscript.

In item #1 below, report all support for the work reported in this manuscript without time limit. For all other items, the time frame for disclosure is the past 36 months.

| | | Name all entities with whom you have this relationship or indicate none (add rows as needed) | Specifications/Comments (e.g., if payments were made to you or to your institution) |
|---|---|---|---|
| Time frame: Since the initial planning of the work | | | |
| 1 | All support for the present manuscript (e.g., funding, provision of study materials, medical writing, article processing charges, etc.) **No time limit for this item.** | ☒ None | |
| | | | |
| | | | |
| | | | Click the tab key to add additional rows. |
| Time frame: past 36 months | | | |
| **2** | Grants or contracts from any entity (if not indicated in item #1 above). | ☒ **None** | |
| | | | |
| | | | |
| | | | |
| **3** | Royalties or licenses | ☒ **None** | |
| | | | |
| | | | |
| | | | |

| | | Name all entities with whom you have this relationship or indicate none (add rows as needed) | Specifications/Comments (e.g., if payments were made to you or to your institution) |
|---|---|---|---|
| **4** | Consulting fees | ☒ **None** | |
| | | | |
| | | | |
| | | | |
| | | | |
| **5** | Payment or honoraria for lectures, presentations, speakers bureaus, manuscript writing or educational events | ☒ **None** | |
| | | | |
| | | | |
| | | | |
| **6** | Payment for expert testimony | ☒ **None** | |
| | | | |
| | | | |
| | | | |
| **7** | Support for attending meetings and/or travel | ☒ **None** | |
| | | | |
| | | | |
| | | | |
| **8** | Patents planned, issued or pending | ☒ **None** | |
| | | | |
| | | | |
| | | | |
| **9** | Participation on a Data Safety Monitoring Board or Advisory Board | ☒ **None** | |
| | | | |
| | | | |
| | | | |
| **10** | Leadership or fiduciary role in other board, society, committee or advocacy group, paid or unpaid | ☒ **None** | |
| | | | |
| | | | |
| | | | |

| | | Name all entities with whom you have this relationship or indicate none (add rows as needed) | Specifications/Comments (e.g., if payments were made to you or to your institution) |
|---|---|---|---|
| **11** | Stock or stock options | ☒ **None** | |
| | | | |
| | | | |
| | | | |
| **12** | Receipt of equipment, materials, drugs, medical writing, gifts or other services | ☒ **None** | |
| | | | |
| | | | |
| | | | |
| **13** | Other financial or non-financial interests | ☒ **None** | |
| | | | |
| | | | |
| | | | |

**Please place an "X" next to the following statement to indicate your agreement:**

☒ I certify that I have answered every question and have not altered the wording of any of the questions on this form.

# ICMJE DISCLOSURE FORM

| | |
|---|---|
| **Date:** | 7/23/2022 |
| **Your Name:** | Alexander Olawaiye |
| **Manuscript Title:** | Phase I Trial of Ribociclib (LEE-011) with Platinum-based Chemotherapy in Recurrent Platinum Sensitive Ovarian Cancer |
| **Manuscript Number (if known):** | 160573-INS-CMED-RV-2 |

In the interest of transparency, we ask you to disclose all relationships/activities/interests listed below that are related to the content of your manuscript. "Related" means any relation with for-profit or not-for-profit third parties whose interests may be affected by the content of the manuscript. Disclosure represents a commitment to transparency and does not necessarily indicate a bias. If you are in doubt about whether to list a relationship/activity/interest, it is preferable that you do so.

The author's relationships/activities/interests should be defined broadly. For example, if your manuscript pertains to the epidemiology of hypertension, you should declare all relationships with manufacturers of antihypertensive medication, even if that medication is not mentioned in the manuscript.

In item #1 below, report all support for the work reported in this manuscript without time limit. For all other items, the time frame for disclosure is the past 36 months.

| | | **Name all entities with whom you have this relationship or indicate none (add rows as needed)** | **Specifications/Comments (e.g., if payments were made to you or to your institution)** |
|---|---|---|---|
| | | Time frame: Since the initial planning of the work | |
| 1 | All support for the present manuscript (e.g., funding, provision of study materials, medical writing, article processing charges, etc.) **No time limit for this item.** | ☒ None | |
| | | | |
| | | | |
| | | | Click the tab key to add additional rows. |
| | | Time frame: past 36 months | |
| **2** | Grants or contracts from any entity (if not indicated in item #1 above). | ☒ **None** | |
| | | | |
| | | | |
| | | | |
| **3** | Royalties or licenses | ☒ **None** | |
| | | | |
| | | | |
| | | | |

| | | Name all entities with whom you have this relationship or indicate none (add rows as needed) | Specifications/Comments (e.g., if payments were made to you or to your institution) |
|---|---|---|---|
| **4** | Consulting fees | ☒ **None** | |
| | | | |
| | | | |
| | | | |
| | | | |
| **5** | Payment or honoraria for lectures, presentations, speakers bureaus, manuscript writing or educational events | ☒ **None** | |
| | | | |
| | | | |
| | | | |
| **6** | Payment for expert testimony | ☒ **None** | |
| | | | |
| | | | |
| | | | |
| **7** | Support for attending meetings and/or travel | ☒ **None** | |
| | | | |
| | | | |
| | | | |
| **8** | Patents planned, issued or pending | ☒ **None** | |
| | | | |
| | | | |
| | | | |
| **9** | Participation on a Data Safety Monitoring Board or Advisory Board | ☒ **None** | |
| | | | |
| | | | |
| | | | |
| **10** | Leadership or fiduciary role in other board, society, committee or advocacy group, paid or unpaid | ☒ **None** | |
| | | | |
| | | | |
| | | | |

| | | Name all entities with whom you have this relationship or indicate none (add rows as needed) | Specifications/Comments (e.g., if payments were made to you or to your institution) |
|---|---|---|---|
| **11** | Stock or stock options | ☒ **None** | |
| | | | |
| | | | |
| | | | |
| **12** | Receipt of equipment, materials, drugs, medical writing, gifts or other services | ☒ **None** | |
| | | | |
| | | | |
| | | | |
| **13** | Other financial or non-financial interests | ☒ **None** | |
| | | | |
| | | | |
| | | | |

**Please place an "X" next to the following statement to indicate your agreement:**

☒ I certify that I have answered every question and have not altered the wording of any of the questions on this form.

# ICMJE DISCLOSURE FORM

| | |
|---|---|
| **Date:** | 7/24/2022 |
| **Your Name:** | Paniti Sukumvanich, MD |
| **Manuscript Title:** | Phase I Trial of Ribociclib (LEE-011) with Platinum-based Chemotherapy in Recurrent Platinum Sensitive Ovarian Cancer |
| **Manuscript Number (if known):** | 160573-INS-CMED-RV-2 |

In the interest of transparency, we ask you to disclose all relationships/activities/interests listed below that are related to the content of your manuscript. "Related" means any relation with for-profit or not-for-profit third parties whose interests may be affected by the content of the manuscript. Disclosure represents a commitment to transparency and does not necessarily indicate a bias. If you are in doubt about whether to list a relationship/activity/interest, it is preferable that you do so.

The author's relationships/activities/interests should be defined broadly. For example, if your manuscript pertains to the epidemiology of hypertension, you should declare all relationships with manufacturers of antihypertensive medication, even if that medication is not mentioned in the manuscript.

In item #1 below, report all support for the work reported in this manuscript without time limit. For all other items, the time frame for disclosure is the past 36 months.

| | | **Name all entities with whom you have this relationship or indicate none (add rows as needed)** | **Specifications/Comments (e.g., if payments were made to you or to your institution)** |
|---|---|---|---|
| **Time frame: Since the initial planning of the work** | | | |
| 1 | All support for the present manuscript (e.g., funding, provision of study materials, medical writing, article processing charges, etc.) **No time limit for this item.** | ☒ None | |
| | | | |
| | | | |
| | | | Click the tab key to add additional rows. |
| **Time frame: past 36 months** | | | |
| **2** | Grants or contracts from any entity (if not indicated in item #1 above). | ☒ **None** | |
| | | | |
| | | | |
| | | | |
| **3** | Royalties or licenses | ☒ **None** | |
| | | | |
| | | | |
| | | | |

| | | Name all entities with whom you have this relationship or indicate none (add rows as needed) | Specifications/Comments (e.g., if payments were made to you or to your institution) |
|---|---|---|---|
| **4** | Consulting fees | ☒ **None** | |
| | | | |
| | | | |
| | | | |
| | | | |
| **5** | Payment or honoraria for lectures, presentations, speakers bureaus, manuscript writing or educational events | ☒ **None** | |
| | | | |
| | | | |
| | | | |
| **6** | Payment for expert testimony | ☒ **None** | |
| | | | |
| | | | |
| | | | |
| **7** | Support for attending meetings and/or travel | ☒ **None** | |
| | | | |
| | | | |
| | | | |
| **8** | Patents planned, issued or pending | ☒ **None** | |
| | | | |
| | | | |
| | | | |
| **9** | Participation on a Data Safety Monitoring Board or Advisory Board | ☒ **None** | |
| | | | |
| | | | |
| | | | |
| **10** | Leadership or fiduciary role in other board, society, committee or advocacy group, paid or unpaid | ☒ **None** | |
| | | | |
| | | | |
| | | | |

| | | Name all entities with whom you have this relationship or indicate none (add rows as needed) | Specifications/Comments (e.g., if payments were made to you or to your institution) |
|---|---|---|---|
| **11** | Stock or stock options | ☒ **None** | |
| | | | |
| | | | |
| | | | |
| **12** | Receipt of equipment, materials, drugs, medical writing, gifts or other services | ☒ **None** | |
| | | | |
| | | | |
| | | | |
| **13** | Other financial or non-financial interests | ☒ **None** | |
| | | | |
| | | | |
| | | | |

**Please place an "X" next to the following statement to indicate your agreement:**

☒ I certify that I have answered every question and have not altered the wording of any of the questions on this form.

# ICMJE DISCLOSURE FORM

**Date:** 7/23/2022

**Your Name:** Sarah Taylor, MD

**Manuscript Title:** Phase I Trial of Ribociclib (LEE-011) with Platinum-based Chemotherapy in Recurrent Platinum Sensitive Ovarian Cancer

**Manuscript Number (if known):** 160573-INS-CMED-RV-2

In the interest of transparency, we ask you to disclose all relationships/activities/interests listed below that are related to the content of your manuscript. "Related" means any relation with for-profit or not-for-profit third parties whose interests may be affected by the content of the manuscript. Disclosure represents a commitment to transparency and does not necessarily indicate a bias. If you are in doubt about whether to list a relationship/activity/interest, it is preferable that you do so.

The author's relationships/activities/interests should be defined broadly. For example, if your manuscript pertains to the epidemiology of hypertension, you should declare all relationships with manufacturers of antihypertensive medication, even if that medication is not mentioned in the manuscript.

In item #1 below, report all support for the work reported in this manuscript without time limit. For all other items, the time frame for disclosure is the past 36 months.

| | | Name all entities with whom you have this relationship or indicate none (add rows as needed) | Specifications/Comments (e.g., if payments were made to you or to your institution) |
|---|---|---|---|
| **Time frame: Since the initial planning of the work** | | | |
| 1 | All support for the present manuscript (e.g., funding, provision of study materials, medical writing, article processing charges, etc.) **No time limit for this item.** | ☒ None | |
| | | | |
| | | | |
| | | | Click the tab key to add additional rows. |
| **Time frame: past 36 months** | | | |
| 2 | Grants or contracts from any entity (if not indicated in item #1 above). | ☒ **None** | |
| | | | |
| | | | |
| | | | |
| 3 | Royalties or licenses | ☒ **None** | |
| | | | |
| | | | |
| | | | |

| | | Name all entities with whom you have this relationship or indicate none (add rows as needed) | Specifications/Comments (e.g., if payments were made to you or to your institution) |
|---|---|---|---|
| 4 | Consulting fees | ☒ None | |
| | | | |
| | | | |
| | | | |
| | | | |
| 5 | Payment or honoraria for lectures, presentations, speakers bureaus, manuscript writing or educational events | ☒ None | |
| | | | |
| | | | |
| | | | |
| 6 | Payment for expert testimony | ☒ None | |
| | | | |
| | | | |
| | | | |
| 7 | Support for attending meetings and/or travel | ☒ None | |
| | | | |
| | | | |
| | | | |
| 8 | Patents planned, issued or pending | ☒ None | |
| | | | |
| | | | |
| | | | |
| 9 | Participation on a Data Safety Monitoring Board or Advisory Board | ☒ None | |
| | | | |
| | | | |
| | | | |
| 10 | Leadership or fiduciary role in other board, society, committee or advocacy group, paid or unpaid | ☒ None | |
| | | | |
| | | | |
| | | | |

| | | Name all entities with whom you have this relationship or indicate none (add rows as needed) | Specifications/Comments (e.g., if payments were made to you or to your institution) |
|---|---|---|---|
| **11** | Stock or stock options | ☒ **None** | |
| | | | |
| | | | |
| | | | |
| **12** | Receipt of equipment, materials, drugs, medical writing, gifts or other services | ☒ **None** | |
| | | | |
| | | | |
| | | | |
| **13** | Other financial or non-financial interests | ☒ **None** | |
| | | | |
| | | | |
| | | | |

**Please place an "X" next to the following statement to indicate your agreement:**

☒ I certify that I have answered every question and have not altered the wording of any of the questions on this form.

# ICMJE DISCLOSURE FORM

**Date:** 7/21/2021

**Your Name:** Ronald Buckanovich

**Manuscript Title:** Phase I Trial of Ribociclib (LEE-011) with Platinum-based Chemotherapy in Recurrent Platinum Sensitive Ovarian Cancer

**Manuscript Number (if known):** 160573-INS-CMED-RV-2

In the interest of transparency, we ask you to disclose all relationships/activities/interests listed below that are related to the content of your manuscript. "Related" means any relation with for-profit or not-for-profit third parties whose interests may be affected by the content of the manuscript. Disclosure represents a commitment to transparency and does not necessarily indicate a bias. If you are in doubt about whether to list a relationship/activity/interest, it is preferable that you do so.

The author's relationships/activities/interests should be defined broadly. For example, if your manuscript pertains to the epidemiology of hypertension, you should declare all relationships with manufacturers of antihypertensive medication, even if that medication is not mentioned in the manuscript.

In item #1 below, report all support for the work reported in this manuscript without time limit. For all other items, the time frame for disclosure is the past 36 months.

| | | **Name all entities with whom you have this relationship or indicate none (add rows as needed)** | **Specifications/Comments (e.g., if payments were made to you or to your institution)** |
|---|---|---|---|
| | | **Time frame: Since the initial planning of the work** | |
| 1 | All support for the present manuscript (e.g., funding, provision of study materials, medical writing, article processing charges, etc.) **No time limit for this item.** | ☐ None | |
| | | Novaritis | Provided study drug and supported trial |
| | | | |
| | | | Click the tab key to add additional rows. |
| | | **Time frame: past 36 months** | |
| **2** | Grants or contracts from any entity (if not indicated in item #1 above). | ☒ **None** | |
| | | | |
| | | | |
| | | | |
| **3** | Royalties or licenses | ☐ **None** | |
| | | Co-founder of Tradewinds Bioscience | |
| | | | |
| | | | |

| | | Name all entities with whom you have this relationship or indicate none (add rows as needed) | Specifications/Comments (e.g., if payments were made to you or to your institution) |
|---|---|---|---|
| **4** | Consulting fees | ☒ **None** | |
| | | | |
| | | | |
| | | | |
| | | | |
| **5** | Payment or honoraria for lectures, presentations, speakers bureaus, manuscript writing or educational events | ☒ **None** | |
| | | | |
| | | | |
| | | | |
| **6** | Payment for expert testimony | ☒ **None** | |
| | | | |
| | | | |
| | | | |
| **7** | Support for attending meetings and/or travel | ☒ **None** | |
| | | | |
| | | | |
| | | | |
| **8** | Patents planned, issued or pending | ☒ **None** | |
| | | Nothing related to this study | Patent pending on ALDH inhibitors as immunotherapeutic adjunct |
| | | | |
| | | | |
| **9** | Participation on a Data Safety Monitoring Board or Advisory Board | ☐ **None** | |
| | | Abound Bio | Advisory Board |
| | | | |
| | | | |
| **10** | Leadership or fiduciary role in other board, society, committee or advocacy group, paid or unpaid | ☐ **None** | |
| | | Ovarian Cancer Research Alliance | scientific advisory board member |
| | | | |
| | | | |

| | | Name all entities with whom you have this relationship or indicate none (add rows as needed) | Specifications/Comments (e.g., if payments were made to you or to your institution) |
|---|---|---|---|
| **11** | Stock or stock options | ☒ **None** | |
| | | | |
| | | | |
| | | | |
| **12** | Receipt of equipment, materials, drugs, medical writing, gifts or other services | ☒ **None** | |
| | | | |
| | | | |
| | | | |
| **13** | Other financial or non-financial interests | ☒ **None** | |
| | | | |
| | | | |
| | | | |

**Please place an "X" next to the following statement to indicate your agreement:**

☒ I certify that I have answered every question and have not altered the wording of any of the questions on this form.

# ICMJE DISCLOSURE FORM

**Date:** 8/8/2022

**Your Name:** Leonard Frisbie, MS

**Manuscript Title:** Phase I Trial of Ribociclib (LEE-011) with Platinum-based Chemotherapy in Recurrent Platinum Sensitive Ovarian Cancer

**Manuscript Number (if known):** 160573-INS-CMED-RV-2

In the interest of transparency, we ask you to disclose all relationships/activities/interests listed below that are related to the content of your manuscript. "Related" means any relation with for-profit or not-for-profit third parties whose interests may be affected by the content of the manuscript. Disclosure represents a commitment to transparency and does not necessarily indicate a bias. If you are in doubt about whether to list a relationship/activity/interest, it is preferable that you do so.

The author's relationships/activities/interests should be defined broadly. For example, if your manuscript pertains to the epidemiology of hypertension, you should declare all relationships with manufacturers of antihypertensive medication, even if that medication is not mentioned in the manuscript.

In item #1 below, report all support for the work reported in this manuscript without time limit. For all other items, the time frame for disclosure is the past 36 months.

| | | **Name all entities with whom you have this relationship or indicate none (add rows as needed)** | **Specifications/Comments (e.g., if payments were made to you or to your institution)** |
|---|---|---|---|
| Time frame: Since the initial planning of the work | | | |
| 1 | All support for the present manuscript (e.g., funding, provision of study materials, medical writing, article processing charges, etc.) **No time limit for this item.** | ☒ None | |
| | | | |
| | | | |
| | | | Click the tab key to add additional rows. |
| Time frame: past 36 months | | | |
| **2** | Grants or contracts from any entity (if not indicated in item #1 above). | ☒ **None** | |
| | | | |
| | | | |
| | | | |
| **3** | Royalties or licenses | ☒ **None** | |
| | | | |
| | | | |
| | | | |

| | | Name all entities with whom you have this relationship or indicate none (add rows as needed) | Specifications/Comments (e.g., if payments were made to you or to your institution) |
|---|---|---|---|
| **4** | Consulting fees | ☒ **None** | |
| | | | |
| | | | |
| | | | |
| | | | |
| **5** | Payment or honoraria for lectures, presentations, speakers bureaus, manuscript writing or educational events | ☒ **None** | |
| | | | |
| | | | |
| | | | |
| **6** | Payment for expert testimony | ☒ **None** | |
| | | | |
| | | | |
| | | | |
| **7** | Support for attending meetings and/or travel | ☒ **None** | |
| | | | |
| | | | |
| | | | |
| **8** | Patents planned, issued or pending | ☒ **None** | |
| | | | |
| | | | |
| | | | |
| **9** | Participation on a Data Safety Monitoring Board or Advisory Board | ☒ **None** | |
| | | | |
| | | | |
| | | | |
| **10** | Leadership or fiduciary role in other board, society, committee or advocacy group, paid or unpaid | ☒ **None** | |
| | | | |
| | | | |
| | | | |

| | | Name all entities with whom you have this relationship or indicate none (add rows as needed) | Specifications/Comments (e.g., if payments were made to you or to your institution) |
|---|---|---|---|
| **11** | Stock or stock options | ☒ **None** | |
| | | | |
| | | | |
| | | | |
| **12** | Receipt of equipment, materials, drugs, medical writing, gifts or other services | ☒ **None** | |
| | | | |
| | | | |
| | | | |
| **13** | Other financial or non-financial interests | ☒ **None** | |
| | | | |
| | | | |
| | | | |

**Please place an "X" next to the following statement to indicate your agreement:**

☒ I certify that I have answered every question and have not altered the wording of any of the questions on this form.

Supplemental information: Full clinical trial protocol

PROTOCOL

**Phase I Trial of Ribociclib (ribociclib (LEE-011)) with Platinum-based Chemotherapy in Recurrent Platinum Sensitive Ovarian Cancer**

**HCC #:** 18-006

**IND#:** 133614

**Phase:** I

**Protocol Version Date:** 2.16 dated 3/31/2021

**Investigational Agent**: ribociclib (LEE-011)

**Principle Investigator:** **Lan G. Coffman, MD, Ph.D.**
**Magee-Womens Hospital of UPMC**
**300 Halket Street Pittsburgh, PA 15213**
**(412) 641-2016**
**coffmanl@mwri.magee.edu**

**Biostatistician:** Daniel Normolle, Ph.D
Associate Professor of Biostatistics
Director, UPMC Hillman Cancer Center Biostatistics Facility
Sterling Plaza Suite 325
201 North Craig Street
Pittsburgh, PA 15213
dpn7@pitt.edu

**Source(s) of Support:** Novartis, Inc., UPMC Hillman Cancer Center

**Study Monitor**: Education and Compliance Office for Human Subject Research
Research Conduct and Compliance Office University of Pittsburgh
3500 Fifth Avenue, Suite 205
Pittsburgh, PA 15213

TABLE OF CONTENTS

LIST OF TABLES ........ 7

LIST OF FIGURES ........ 7

ABBREVIATIONS ........ 8

STUDY SYNOPSIS ........ 10

1.0 BACKGROUND AND RATIONALE ........ 16

1.1 Disease Background ........ 16

1.2 Study Agent(s) Background and Associated Known Toxicities ........ 16

1.3 Other Agents ........ 17

1.4 Rationale ........ 18

1.5 Correlative Studies ........ 21

2.0 STUDY OBJECTIVES ........ 22

2.1 Primary Objectives ........ 22

2.2 Secondary Objectives ........ 22

2.3 Primary Endpoints ........ 22

3.0 PATIENT ELIGIBILITY ........ 22

3.1 Inclusion Criteria ........ 22

3.2 Exclusion Criteria ........ 24

4.0 SUBJECT SCREENING AND REGISTRATION PROCEDURES ........ 25

5.0 TREATMENT PLAN ........ 25

5.1 Treatment Dosage and Administration ........ 26

5.2 Toxicities and Dosing Delays/Dose Modifications ........ 28

5.3 Concomitant Medications/Treatments ........ 35

5.7 Off Study Criteria ........ 40

5.8 Patient Replacement ........ 41

**Patients enrolled who did not receive a dose of ribociclib (LEE-011) or platinum + taxane will not contribute to MTD determination and will be replaced ........ 41**

**6.0 STUDY PROCEDURES ........ 41**

**6.1** Screening/Baseline Procedures ........ 41

6.1.1 Informed Consent ........ 41

6.1.2 Medical history ........ 41

6.1.3 Demographics ........ 41

6.1.4 Review subject eligibility criteria ........ 41

6.1.5 Review previous andconcomitant medications ........ 41

6.1.6 Physical exam including vital signs, height and weight ........ 41

6.1.7 Performance status ........ 42

6.1.8 Adverse event assessment ........ 42

6.1.9 Laboratory assessment ........ 42

6.1.10 Acquisition of previous tissue sample for correlative studies ........ 42

6.1.11 Pregnancy test (for females of childbearing potential) ........ 42

**6.2** Procedures During Treatment ........ 42

6.2.1.1 Day 1 (± 2days) (week 1 ribociclib (LEE-011) +platinum+ taxane) ........ 42

6.2.2 Ribociclib (LEE-011) Maintenance ........ 43

**6.3** Follow-Up Procedures ........ 43

**6.4** Time and Events Table ........ 44

7.1.1 Definitions........................................................................................................ 45

7.1.2 Disease Parameters ........................................................................................ 46

7.1.3 Guidelines for Evaluation of Measurable Disease ....................................... 47

7.1.4 Response Criteria ........................................................................................... 47

7.1.5 Duration of Response...................................................................................... 49

7.1.6 Progression-Free Survival............................................................................... 50

7.2 Safety/Tolerability ............................................................................................ 50

**8.0 ADVERSE EVENTS ...........................................................................50**

**8.1** Experimental Therapy (ribociclib (LEE-011))............................................ 50

8.1.1 Contraindications ........................................................................................... 50

8.1.2 Drug Interactions ........................................................................................... 50

8.1.3 Adverse Reactions (see section 8.3)............................................................... 50

**8.2** Adverse Event Reporting Requirements ................................................... 50

**8.3** Definitions (adapted from NCI Guidelines: Adverse Event Reporting Requirements)...51

8.3.1 Adverse Event................................................................................................ 51

8.3.2 Serious Adverse Event.................................................................................... 51

8.3.3 Expected Adverse Events ............................................................................... 52

8.3.4 Unexpected Adverse Event............................................................................. 52

**8.4** Adverse Event Characteristics.................................................................... 52

8.4.1 CTCAEv5 Term.............................................................................................. 52

8.4.2 Attribution of the AE....................................................................................... 53

**8.5** Serious Adverse Event Reporting Guidelines ............................................. 53

**8.8** Stopping Rules............................................................................................. 55

**9.0 DRUG INFORMATION ....................................................................55**

**9.1** Novartis investigational drug, LEE011 .......................................................................... 55

**9.2** Chemotherapeutics ........................................................................................................ 57

• Commercial supply of carboplatin, cisplatin, paclitaxel, and docetaxel will beused and prepared/administered per institutional standards andthe package insert......................................................................................... 57

**10.0 CORRELATIVES/SPECIAL STUDIES ...................................................57**

**10.1** Sample Collection Guidelines ........................................................................................ 57

**10.2** Specimen Banking .......................................................................................................... 57

**11.0 STATISTICAL CONSIDERATION .........................................................58**

**11.1** Study Design/ Endpoints................................................................................................. 58

11.1.1 Endpoint(s) ........................................................................................................ 58

11.1.2 Study Design ...................................................................................................... 58

11.1.3 Dose allocation .................................................................................................. 59

11.1.4 Cohort size......................................................................................................... 60

11.1.5 Evaluability ........................................................................................................ 60

11.1.6 Operating Characteristics................................................................................... 60

11.1.7 Early Stopping Guidelines .................................................................................. 61

**11.2** Sample Size/ Accrual Rate ............................................................................................. 62

**11.3** Stratification Factors ...................................................................................................... 62

**11.4** Analysis of Secondary Endpoints ................................................................................... 62

12.0 **DATA AND SAFETY MONITORING.......................................................** 62

13.0 References

**14.0** APPENDDICES............................................................................................................64

LIST OF TABLES

Table 1. DLT Criteria ....................27
Table 2. Dose-Escalation Criteria ....................28
Table 3. Hematological Toxicity Dose Reductions for ribociclib (LEE-011) ....................30
Table 4. Hepatotoxicity Dose Reductions for ribociclib (LEE-011) ....................31
Table 5. Dose modification for QTc prolongation ....................35
Table 6. Dose modification for Interstitial lung disease/pneumonitis ....................36
Table 7. Ribociclib (LEE-011) Dose Adjustment and Management Recommendation for All Other Adverse Reactions ....................37
Table 8. Permitted concomitant therapyrequiring caution ....................39
Table 9. Prohibited drugs and therapy requiring caution and/or action ....................42
Table 10. Study calendar during chemotherapy ....................48
Table 11. Study calendar during maintenance ribociclib (LEE-011)… ....................49

**LIST OF FIGURES**

Figure 1. LEE-011 decreases growth in $Rb^{wt}$ ovarian cancer cells, but not in $Rb^{null}$ lines ......18
Figure 2. Impact of LEE-011 treatment combined withcisplatin therapy ....................18
Figure 3. Impact of combined LEE-011 and cisplatin in long-term cell recovery ....................20
Figure 4. LEE-011 decreases ovarian cancer growth in murinexenograft model ....................21

**ABBREVIATIONS**

| | |
|---|---|
| AE | Adverse Event |
| ALT | Alanine Aminotransferase |
| ALC | Absolute Lymphocyte Count |
| AST | Aspartate Aminotransferase |
| BUN | Blood Urea Nitrogen |
| CBC | Complete Blood Count |
| CMP | Comprehensive Metabolic Panel |
| CR | Complete Response |
| CT | Computed Tomography |
| CTCAE | Common Terminology Criteria for Adverse Events |
| CTO | Clinical Trials Office |
| DLT | Dose Limiting Toxicity |
| DSMB | Data and Safety Monitoring Board |
| H&P | History & Physical Exam |
| HRPP | Human Research Protections Program |
| IND | Investigational New Drug |
| IRB | Institutional Review Board |
| IV (or iv) | Intravenously |
| MTD | Maximum Tolerated Dose |
| NCI | National Cancer Institute |
| ORR | Overall Response Rate |
| OS | Overall Survival |
| PBMCs | Peripheral Blood Mononuclear Cells |
| PD | Progressive Disease |
| PFS | Progression Free Survival |
| PI | Principal Investigator |
| PO | per os/by mouth/orally |
| PR | Partial Response |
| PRC | Protocol Review Committee |
| Rb | Retinoblastoma |
| SAE | Serious Adverse Event |
| SD | Stable Disease |
| SGOT | Serum Glutamic Oxaloacetic Transaminase |
| SPGT | Serum Glutamic Pyruvic Transaminase |
| UaP | Unanticipated Problem |
| UMCCC | University of Michigan Comprehensive Cancer Center |

## STUDY SCHEMA

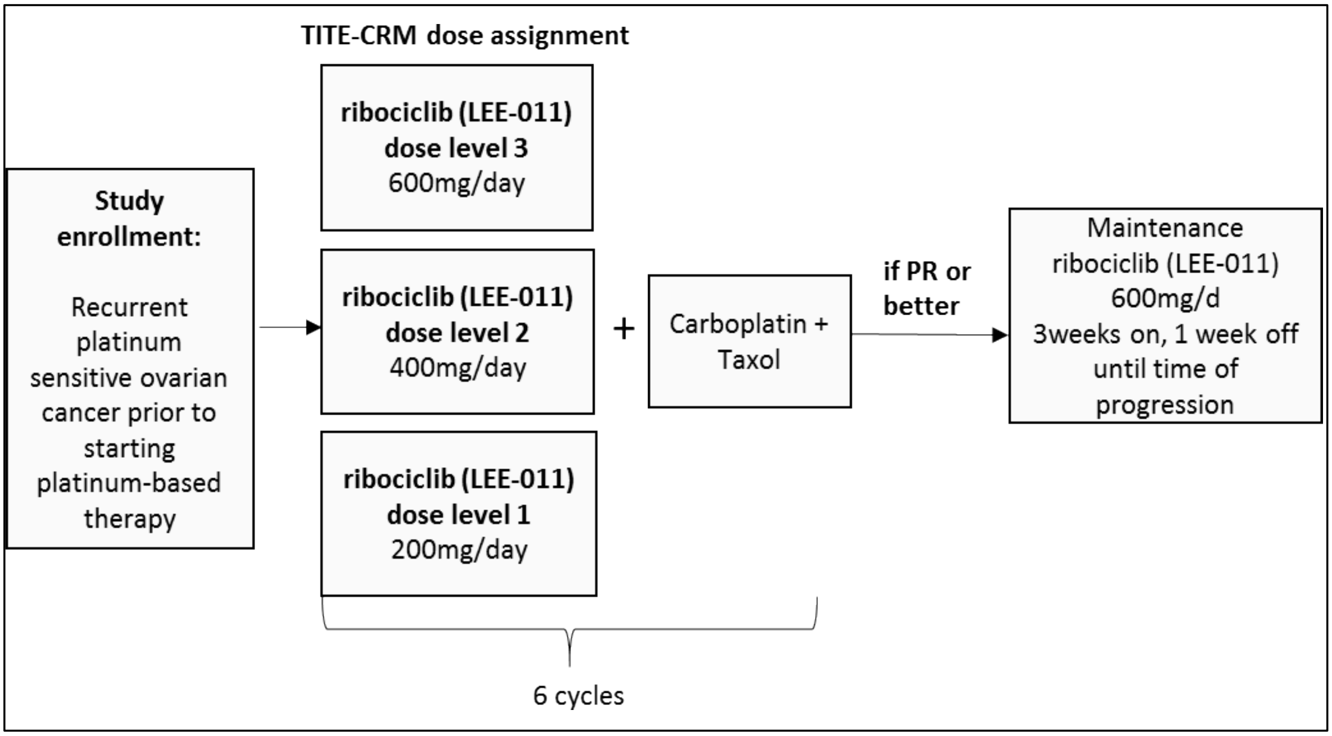

**STUDY SYNOPSIS**

| | |
|---|---|
| Title | Phase I trial of ribociclib (LEE-011) with platinum-based chemotherapy in recurrent platinum sensitive ovarian cancer |
| Phase | Phase 1 |
| Methodology | Open label |
| Study Duration | 38 months |
| Study Center(s) | Single center, University of Pittsburgh Medical Center |
| Objectives | **Primary Objective(s):**<br>Determine the maximal tolerated dose (MTD) of ribociclib (LEE-011) when given with platinum + taxane in platinum-sensitive recurrent ovarian cancer.<br>**Secondary Objective(s):**<br>a) Overall Survival (OS)<br>b) To observe and record anti-tumor activity. Although the clinical benefit of this drug has not yet been established, the intent of offering this treatment is to provide a possible therapeutic benefit, and thus the patient will be carefully monitored for tumor response and symptom relief in addition to safety and tolerability.<br>c) Determine the response rate (RR) and progression free survival (PFS)of ribociclib (LEE-011) with platinum + taxane followed by maintenance ribociclib (LEE-011).<br>d) Further characterize the safety profile of ribociclib (LEE-011) with platinum + taxane in recurrent platinum-sensitive ovarian cancer.<br>e) Correlate RR and PFS with Rb mutational status and NFAT nuclear expression levels from patient tissuesamples |
| Number of Subjects | Approximately 40 patients |

| Inclusion Criteria | |
|---|---|
| | 1. Women ≥18 years old with platinum-sensitive recurrent ovarian, fallopian or primary peritoneal cancer (defined as recurrent disease >6 months after completing last platinum-based chemotherapy) eligible to receive platinum-based doubletchemotherapy.<br>2. Must have had at least 1 prior line of platinum-based therapy<br>3. ECOG 0-1 with life expectancy of ≥ 3months<br>4. Adequate organ function:<br>• Serum creatinine ≤1.5mg/dL or 24-hour clearance≥50mL/min<br>• AST/ALT <2.5x ULN (or <5x ULN if liver metastasis are present)<br>• Total bilirubin ≤ ULN or total bilirubin ≤3.0 x ULN or direct bilirubin ≤1.5 x ULN in patients with well-documented Gilbert's Syndrome.<br>• Hemoglobin ≥9 gm/dl, Platelets ≥100,000/µl, ANC≥1500/µl<br>• INR ≤1.5<br>• Potassium, total calcium (corrected for serum albumin),magnesium, and sodium within normal limits for the institution or corrected to within normal limits with supplements before first dose of study medication<br>5. Screening ECG (defined as the mean of the triplicate ECGs) with QTcF interval at screening ≤450msec (using Fridericia's correction) and resting heart rate 50-90bpm<br>6. Must be able to swallow ribociclib (LEE-011) tablet/capsule<br>7. Documented disease recurrence/progression based on GCIG-RECIST<br>8. Able to provide informed consent and comply with all study protocols<br>9. Treated CNS metastasis allowed if treatment is complete ≥8 weeks prior to enrollment. Patients must be asymptomatic off systemic corticosteroids for at least 4 weeks after completion of radiation therapy. CNS disease must be stable or regressed on repeat imaging performed at least 4 weeks after completion oftherapy.<br>10. Women of child-bearing potential (those who have had a menstrual cycle within the last year and have not had a tubal ligation orsurgical removal of both ovaries and/or hysterectomy) must agree to abstain from vaginal intercourse or use and continue highly effective methods of contraception for 3 weeks after discontinuation of study treatment.<br>11. Patients must have histologically confirmed malignancy that is metastatic or unresectable and for which standard curative or palliative measures do not exist or are no longer effective. |

UPMC | HILLMAN CANCER CENTER

| | |
|---|---|
| Exclusion Criteria | 1. Borderline or low-malignant potential histology.<br>2. Platinum-resistant disease (as defined as progressive diseasewithin6months of completion of chemotherapy with a platinum agent)<br>3. Grade 3 baselineneuropathy.<br>4. Known hypersensitivity to any of the excipients of ribociclib (LEE-011), including peanuts andsoy<br>5. Prior use of CDK4/6 inhibitors.<br>6. Congenital long QT syndrome or family history of unexpected sudden cardiac death<br>7. Concurrent malignancy or malignancy within 3 years prior to starting study drug, with the exception of adequately treated basal or squamous cell carcinoma, non-melanomatous skin cancer or curatively resected cervical cancer or per physician discretion that the previous cancer was adequately treated with curative intent and unlikely to recur (the study PI must concur with thisdetermination)<br>8. Impairment of gastrointestinal (GI) function or disease thatmay significantly alter the absorption of the studydrugs<br>9. History of HIV infection<br>10. Patient has any other concurrent severe and/or uncontrolled medical condition that would, in the investigator's judgment, cause unacceptable safety risks and contraindicate patient's participation in the clinical study or compromise compliance with the protocol (e.g. chronic pancreatitis, chronic activehepatitis, active untreated or uncontrolled fungal, bacterial or viral infections, etc.).<br>11. Clinically significant, uncontrolled heart disease and/orcardiac repolarization abnormalities, including any of the following:<br>a. Heart Association functional classification III-IV)<br>b. Documented cardiomyopathy<br>c. Left Ventricular Ejection Fraction (LVEF) <50% as determined by Multiple Gated acquisition (MUGA) scan or echocardiogram (ECHO) at screening<br>d. Clinically significant cardiac arrhythmias (e.g. ventricular tachycardia), complete left bundle branch block, high-grade AV block (e.g. bifascicular block, Mobitz type II and third-degree AV block)<br>e. Long QT syndrome or family history of idiopathic sudden death or congenital long QT syndrome, or any of the following:<br>i. Risk factors for Torsades de Pointe (TdP) including uncorrected hypokalemia or hypomagnesaemia, historyof cardiac failure, or history of clinically significant/symptomatic bradycardia.<br>ii. Concomitant use of medication(s) with a known risk to prolong the QT interval and/or known to cause Torsades de Pointe that cannot be discontinued (within 5 half-lives or 7 days prior to starting study drug) or replaced by safe alternative medication<br>iii. Inability to determine the QT interval on screening(QTcF using Fridericia's correction)<br>f. Systolic blood pressure (SBP) >160 mmHg or <90mmHg at screening<br>g. History of acute coronary syndromes (including myocardial infarction, unstable angina, coronary artery bypass grafting, coronary angioplasty, or stenting) or symptomatic pericarditis within 6 months prior to screening |

| | |
|---|---|
| | 12. Use of prohibited medications (see section 5.3) that cannot be changed to an alternative therapy<br>13. Patient is currently receiving or has received systemic corticosteroids ≤2 weeks prior to starting study drug, or who have not fully recovered from side effects of such treatment.<br>a. The following uses of corticosteroids are permitted: single doses, topical applications (e.g., for rash), inhaled sprays (e.g., for obstructive airways diseases), eye drops or local injections (e.g., intra-articular)<br>14. Patient is currently receiving warfarin or other coumadin-derived anticoagulant for treatment, prophylaxis or otherwise. Therapy with heparin, low molecular weight heparin (LMWH) or fondaparinux is allowed.<br>15. Use of herbal supplements unless discontinued ≥7 days prior to initiation of study drug<br>16. Consumption of foods which are strong inducers or inhibitors of CYP3A4/5 has to be discontinued 7 days prior to initiation of study drug<br>17. Pregnancy or lactation<br>18. Participation in a prior investigational study within 30 days prior to enrollment or within 5 half-lives of the investigational product, whichever is longer<br>19. Patient who has received radiotherapy ≤4 weeks or limited field radiation for palliation ≤2 weeks prior to starting study drug, and who has not recovered to grade 1 or better from related side effects of such therapy (exceptions include alopecia) and/or in whom ≥25% of the bone marrow (Ellis, 1961) was irradiated.<br>20. Patient has had major surgery within 14 days prior to starting study drug or has not recovered from major side effects (tumor biopsy is not considered as major surgery).<br>21. Patient has not recovered from all toxicities related to prior anticancer therapies to NCI-CTCAE version 5 Grade ≤1 (Exception to this criterion: patients with any grade of alopecia and/or neuropathy ≤ grade 2 are allowed to enter the study).<br>22. Patient with a Child-Pugh score B or C. |

<table>
<tr>
<td>Study Product(s), Dose, Route, Regimen</td>
<td>
Study Drug: ribociclib (LEE-011)
<ul><li>Dose levels (combined with weekly Carboplatin AUC2 + paclitaxel 60mg/m2):</li></ul>
<table>
<tr><th colspan="4">Dose-Escalation Schedule</th></tr>
<tr><th>Dose level</th><th>Dose of study agent: ribociclib</th><th>Dose of standard agent: Carboplatin[a]</th><th>Dose of standard agent: Paclitaxel[b]</th></tr>
<tr><td>Level 1**</td><td>200 mg/d</td><td>AUC 2</td><td>60 mg/m2</td></tr>
<tr><td>Level 2</td><td>400 mg/d</td><td>AUC 2</td><td>60 mg/m2</td></tr>
<tr><td>Level 3</td><td>600 mg/d</td><td>AUC 2</td><td>60 mg/m2</td></tr>
<tr><td colspan="4">** starting dose level</td></tr>
<tr><td colspan="4">a: For patients who develop allergy to carboplatin, weekly cisplatin 35mg/m2 can be used</td></tr>
<tr><td colspan="4">b: For patients allergic/intolerant of Paclitaxel, docetaxel (30mg/m2) can be used</td></tr>
</table>
<ul>
<li>Ribociclib (LEE-011) will be given on days 1-4, 8-11, and 15-18 with weekly platinum + taxane (given days 1, 8, and 15 of a 28 day cycle) for 6 total cycles of chemotherapy. If women achieve at least a PR as measured after completion of chemotherapy, maintenance ribociclib (LEE-011) at a dose level of 600mg/day will be given 3 weeks on, 1 week off until progression.</li>
<li>Ribociclib (LEE-011) will be administered orally</li>
<li>platinum + taxane will be administered intravenously asper standard dosing.</li>
</ul>
</td>
</tr>
<tr>
<td>Duration of Administration</td>
<td>Patients will receive 6 cycles of platinum + taxane given weekly days 1, 8, and 15 (± 2 days) of a 28 day cycle with ribociclib (LEE-011) as above. Maintenance ribociclib (LEE-011) will start within 6 weeks of completing chemotherapy in women achieving at least a PR and continue (3 weeks on, 1 week off ±2 days) until time of progression. Each patient will have follow up every 3 months as measured from the time of enrollment.</td>
</tr>
<tr>
<td>Reference Therapy</td>
<td>N/A</td>
</tr>
<tr>
<td>Statistical Methodology</td>
<td>
<ul>
<li>Primary endpoint:<br>Occurrence of DLT in each patient with assessment of AEs/SAEs per CTCAE v5 in the first two cycles</li>
<li>Secondary endpoints:<br>Overall Survival (OS), Response rate (RR) and Progression-free survival (PFS) per GCIG-RECIST Tolerability as measured by history & physical exam and laboratory assessment</li>
<li>Correlative/Explorative endpoints:<br>Determine level of association between baseline Rb mutational status as measured via PCR and NFAT nuclear expression via IHC in initial tumor samples with RR and PFS clinical endpoints</li>
</ul>
</td>
</tr>
</table>

| | |
|---|---|
| | • A time-to-event continual reassessment model (TITE-CRM) will be utilized for dose escalation of ribociclib (LEE-011) treatment with platinum + taxane therapy. We anticipate enrolling 40 patients in order to determine the MTD.<br>• DLTs will be assessed for 2 cycles of ribociclib (LEE-011) treatment with platinum + taxane (4 week cycle schedule, total 8weeks).<br>• Rb mutation rates and NFAT4 nuclear expression levels will be correlated with response rate andPFS.<br>• We plan to actively accrue patients for ~20 months with at least 3 months follow up starting from the time of enrollment. |

# 1.0 BACKGROUND AND RATIONALE

## 1.1 Disease Background

Ovarian cancer is the most deadly gynecologic cancer in the US, killing over 14,000 women yearly [1]. Despite a complete clinical remission in response to surgical debulking and platinum based chemotherapy, most patients will relapse within 12-18 months of therapy, develop chemotherapy resistance, and ultimately die of the disease. Improving progression free survival after chemotherapy could improve survival and reduce exposure to cytotoxic chemotherapy. Ovarian cancer recurrence is thought to arise from cells which are "left behind" after chemotherapy that are capable of regenerating bulk tumor, often referred to as cancer stem cell-like cells (CSCs). These cells survive in a quiescent state (alive but not actively dividing) until a point where they are triggered to begin division and re-populate the tumor (similar to normal stem cells which remain dormant until needed for re-population of a tissue after injury) [2, 3]. The "trigger" which moves a CSC from a quiescent state into active division is currently unknown. However, the ability to maintain residual cancer cells in a quiescent state could dramatically improve progression free survival and possibly impact overall survival. Alternatively, the ability to target these CSCs by modulating quiescence pathways, effectively driving these cells into cycle may dramatically chemo-sensitize these inherently resistant cells and may ultimately lead to less disease recurrence.

## 1.2 Study Agent(s) Background and Associated Known Toxicities

Therapies targeted at inducing quiescence/halting cell division is of increasing interest in oncology. Of particular interest are inhibitors of the cell cycle check point proteins, CDK4 and CDK6. Ribociclib (LEE-011) is a small molecule inhibitor of CDK4/6. Ribociclib has been approved by the United States Food and Drug Administration (U.S. FDA) and the European Commission as an initial endocrine-based therapy for the treatment of postmenopausal women with hormone receptor (HR)-positive, human epidermal growth factor receptor 2 (HER2)-negative advanced or metastatic breast cancer in combination with an aromatase inhibitor based on a randomized, double-blind, placebo-controlled, international clinical trial (MONALEESA-2). Additional marketing authorizations are under review by health authorities. Additional phase III clinical trials for the treatment of hormone receptor positive (HR+) breast cancer patients, as well as several other phase I or II clinical studies are being conducted. The toxicity profile in these studies was tolerable, with nausea, neutropenia, and fatigue as the most common side effects [4-10]. Specific toxicity data is described below:

In single agent phase I trials, a total of 308 patients have been treated.

Based on study CLEE011X2101, the recommended dose for expansion (RDE) was declared as 600 mg qd on a 3 weeks on/1 week off schedule. At the RDE, the most common (in at least 20% of patients) adverse events (AEs) related to study treatment were (all grades; grades 3/4): nausea (48.6%; 1.4%), neutropenia (33.8%; 21.6%), fatigue (31.1%; 0%), white blood cell count decrease (31.1%; 13.5%), diarrhoea (31.1%; 2.7%), vomiting (27%;0%), anaemia (21.6%; 2.7%). As of 09-Mar-2017, 153 patients have been treated with single agent ribociclib in the first -inhuman (FIH) phase I study: 134 on the 3 weeks on/1 week off regimen and 19 on the continuous dosing regimen. Out of the 134 patients treated in the 3 weeks on/1 week off regimen; 8 patients were enrolled in the liquid formulation cohort.

**Three weeks on 1 week off regimen**

Patients were treated with increasing doses of ribociclib orally on days 1-21 in a 28-day cycle until the MTD/RDE was determined. All patients discontinued the study treatment for the following primary reasons: disease progression (115 [85.8%] patients); AEs (11 [8.2%] patients); death (1 [0.7 %] patient); withdrawal of consent (4[3.0%] patient); abnormal test procedure results (1 [0.7 %]);

missing (transferred to another ribociclib study 1 [0.7%] patient) and loss to follow up (1 [0.7%] patient). A total of 15 events meeting DLT criteria were observed at the indicated dose s in 14 out of 126 patients evaluable for dose determination and include Grade 3 mucositis/stomatitis (later determined to be due to herpes simplex virus infection) (n=1) at 50 mg, Grade 3 pulmonary embolism (n=1) at 280 mg, Grade 4 neutropenia (n=1) at 400 mg, Grade 3 febrile neutropenia
(n=1), Grade 3 QT prolongation (n=1), Grade 4 thrombocytopenia (n=1), Grade 4 neutropenia (n=1), Grade 4 platelet count decrease (n =1) at 600 mg, Grade 4 thrombocytopenia (n=2) at 750 mg, Grade 3 asymptomatic QTcF prolongation with Grade 3 neutropenia (n=1) at 900 mg and Grade 4 febrile neutropenia (n=1) and Grade 4 thrombocytopenia (n=1) at 1200 mg.
The most frequently reported AEs (≥20%), regardless of grade, causality and ribociclib dose were: nausea (52.2%); fatigue (42.5%); diarrhea (37.3%); vomiting (35.8%); neutropenia (35.1%); anemia (33.6%); thrombocytopenia (23.9%); white blood cell count decreased (23.9%); decreased appetite (23.1%); constipation (22.4%); leukopenia (21.6%) and dyspnea (20.9%).

**Continuous dosing regimen**

Daily ribociclib of a 28-day cycle at 600 mg (n=7), 400 mg (n=6) and 300 mg (n=6) was evaluated. All patients discontinued the study treatment for the following primary reasons: disease progression (16 [84.2%] patients); AEs (2 [10.5%] patients) and withdrawal of consent (1[5.3%] patient). A total of 3 events meeting DLT criteria were observed at the indicated doses in the 3 out of 19 patients evaluable for dose determination and include Grade 3 ALT increase (n=1) at 300mg; Grade 4 neutropenia (n=1) and Grade 3 neutropenia at 400 mg (n=1).
The most frequently reported AEs (≥20%), regardless of grade, causality and ribociclib dose were: nausea (63.2%); fatigue and neutropenia (42.1% each); diarrhea, white blood cell count decrease and abdominal pain (31.6% each); anemia, hyperglycemia and blood creatinine increased (26.3% each); vomiting, decreased appetite, constipation, asthenia, cough, ALT increased and AST increased (21.1% each).

### 1.3 Other Agents

Standard of care in recurrent platinum-sensitive ovarian cancer (as defined as disease recurrence >6 months after last platinum-based therapy) is retreatment with a platinum agent. The most common regimen with best survival data is the doublet of carboplatin and paclitaxel [11]. Unfortunately, disease inevitably recurs necessitating further lines of cytotoxic therapy.

### 1.4 Rationale

Interestingly, in normal stem cells, a master regulatory gene NFAT1 inhibits CDK4/6 expression and induces cellular quiescence. We have found that ovarian CSCs express NFAT4 which regulates CDK4/6 and similarly induces CSC quiescence. Given the observation that down regulation of CDK4/6 can induce quiescence, we have begun to assess the impact of the CDK4/6 inhibitor ribociclib (LEE-011) on ovarian cancer cell proliferation/quiescence. Treatment of ovarian cancer cells with ribociclib (LEE-011) alone was associated with a dose dependent reduction in cell growth (Fig 1A, C). Suggesting growth reduction is an on target effect, Rb mutant lines are resistant to ribociclib (LEE-011) (Fig 1 B, C). While cells expressed markers of senescence (Bgal etc., data not shown), cells showed slow steady growth suggesting a pseudo-senescent state. Consistent with this, washout of drug allowed full cell recovery (Fig 1D).

**Figure 1. LEE-011 decreases growth in Rb$^{wt}$ ovarian cancer cells, but not in Rb$^{null}$ lines**

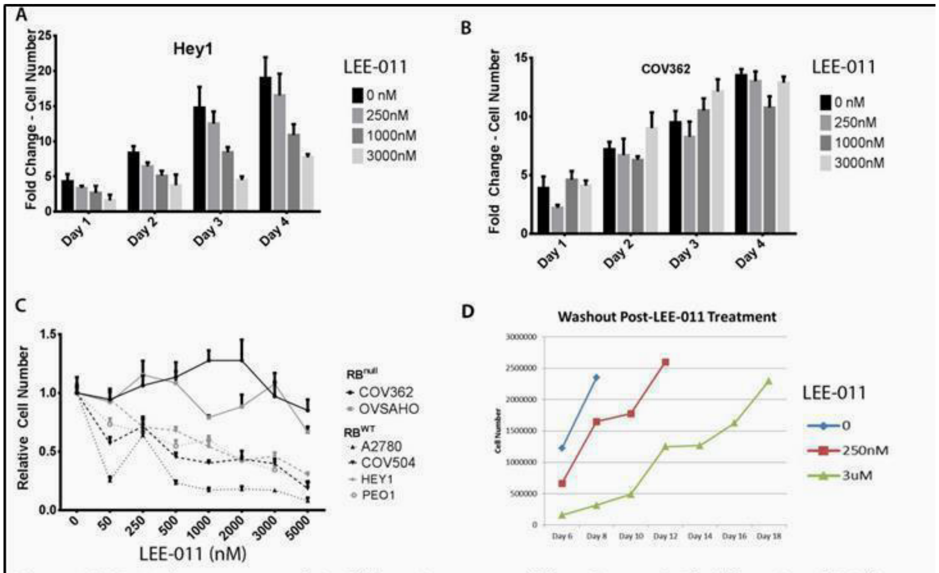


Figure 1: LEE-011 decreases growth in Rb$^{WT}$ ovarian cancer cell lines, but not in Rb$^{null}$ lines. A and B Cell counts of the Rb$^{WT}$ HEY1 line (A) and the Rb$^{null}$ COV362 line demonstrating decreased growth of HEY1 cells but not COV362 cells.C. Growth curves for multiple ovarian cancer cell lines treated with increasing doses of LEE-011. D. Cell growth of HEY1 cells after removal of LEE-011.

Following drug washout, we treated cells 48 hours (Fig 2C) or 24 hours prior to chemotherapy (Fig 2B) treatment or concurrent with chemotherapy (Fig 2A) and tracked cell numbers in an MTT assay at 72 hours. This was performed to determine if ribociclib (LEE-011) could act as a chemosensitizer by synchronizing cells in G1 and then driving cells into cycle. In all three cases, the addition of ribociclib (LEE-011) reduced cell growth. However, when cell numbers were normalized for the anticipated growth reduction associated with ribociclib (LEE-011) treatment we found that 48 hour pre-treatment with a 24 hour washout and concurrent treatment synergized with chemotherapy while, 24 hour pretreatment did not (Fig 2Bii).

**Figure 2. Impact of LEE-011 treatment combined with cisplatin therapy**

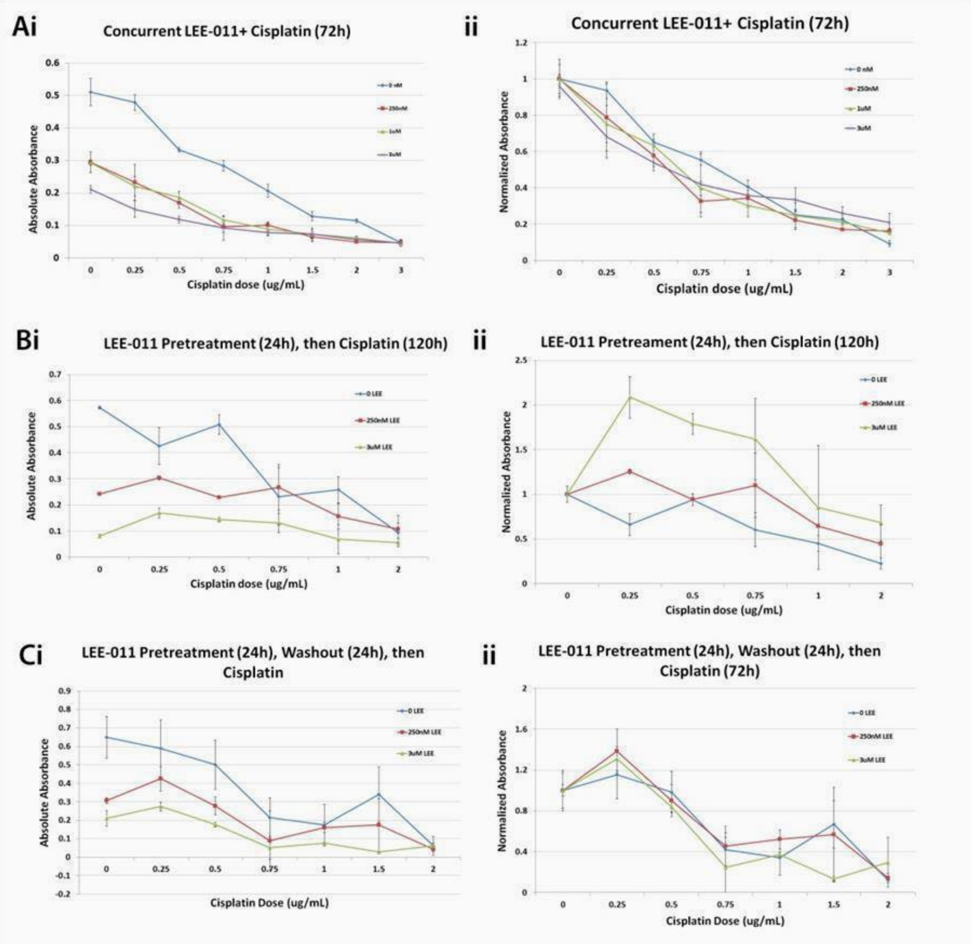


**Figure 2. Impact of LEE treatment combined with cisplatin therapy. A (i)** Absolute absorbance and (ii) normalized absorbance of Hey1 ovarian cancer cells treated with concurrently with cisplatin and increasing doses of LEE-011. B. **(i)** Absolute absorbance and (ii) normalized absorbance of Hey1 ovarian cancer cells treated with LEE-011 24 hours prior to cisplatin treatment. C. **(i)** Absolute absorbance and (ii) normalized absorbance of Hey1 ovarian cancer cells treated with LEE-011 48hrs prior to cisplating therapy. In all cases, LEE-011 treatment decreased absolute cell numbers. However when normalized for inital reductions in cell counts associated with LEE-011 pretreatment, 24h pretreatment with LEE-011 appeared to reduce the effectiveness of cisplatin at lower doses.

To further explore the role of LEE in combination with chemotherapy, we performed long-term cell recovery assays with HEY1 cells. Cells were treated with cisplatin for 3 days, followed immediately with ribociclib (LEE-011) maintenance therapy for 12 days. Unlike ribociclib (LEE-011) maintenance therapy alone (Fig 1) which resulted in growth delay, maintenance therapy after cisplatin demonstrated complete growth arrest at the highest dose of ribociclib (LEE-011) (Fig 3A). Concurrent ribociclib (LEE-011) and cisplatin demonstrated a significant growth delay compared to cisplatin alone (Fig 3B). Concurrent ribociclib (LEE-011) and cisplatin therapy followed by ribociclib (LEE-011) maintenance therapy demonstrated complete growth arrest at both high and intermediate dose ribociclib (LEE-011) (Fig 3C). Pretreatment with ribociclib (LEE-011) followed by cisplatin treatment delayed growth at the highest ribociclib (LEE-011) doses, but had little impact at the lower doses (Fig 3D). Interestingly, cisplatin treatment of the Rb mutant COV362 cells followed by maintenance therapy, while less effective than similar treatment in HEY1 cells, demonstrated significant growth reductions at intermediate and high doses of ribociclib (LEE-011) (Fig 3E). To further explore ribociclib's ability (LEE-011) to completely arrest cell growth following chemotherapy (as opposed to reduce growth in the absence of chemotherapy), we evaluated the impact of ribociclib (LEE-011) on cancer stem-like cells. We found that ribociclib (LEE-011) treatment, leads to significant reduction in the percent of ALDH+ ovarian cancer stem-like cells in both HEY1 (Rb Wt) and COV362 cells (Rb mut) (Fig 3F).

**Figure 3. Impact of combined LEE-011 and cisplatin in long-term cell recovery**

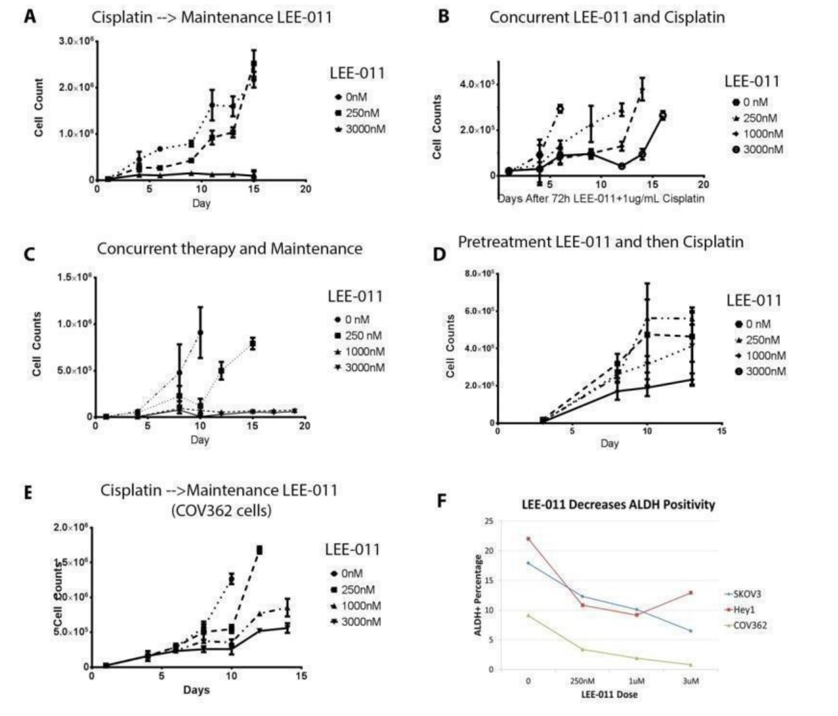


Figure 3.. impact of combined LEE-011 and cisplatin in long-term cell recovery. A. HEY1 cell growth curves following treatment with cisplatin (0.5ug/ml) followed by L EE-011 maintenance therapy with the indicated doses. B. HEY1 cell growth curves following treatment with cisplatin concurrent with the indicated doses of LEE-011. C. HEY1 cell growth curves following treatment with concurrent cisplatin and LEE-011 therapy followed by LEE-011 maintenance therapy. D. HEY1 cell growth curves following pre-treatment with the indicated doses of LEE-011 (24 hours of treatment and 24 hour washout) followed by cisplatin therapy. E. COV362 cell growth curves following treatment with cisplatin (0.5ug/ml) followed by L EE-011 maintenance therapy with the indicated doses. F. percentage of ALDH+ CSC in the indicated cell lines with increasing doses of LEE-011.

Taken together this data suggests that CDK4/6 inhibition at the time of cisplatin-induced DNA damage leads to a more permanent growth arrest. This is consistent with recent studies by Gustavo Baldassarre at the NCI Italy (presented at 2015 AACR Ovarian Cancer special emphasis meeting) who demonstrated CDK6 inhibition, via down regulation of FOX03a, inhibits ATR and thereby leads to platinum sensitization.

To test in vivo efficacy, ovarian cancer xenografts were created in NSG mice and treated with vehicle (control) or ribociclib (LEE-011) alone (50mg/kg per day). In addition we performed a maintenance study—cells were treated with cisplatin 1mg/kg/day x3 days and then treated with ribociclib (LEE-011) (50mg/kg/day) starting 24hrs after completion of cisplatin. Ribociclib (LEE-011) treatment following cisplatin treatment demonstrated a pronounced decrease in tumor growth (Fig 4). Studies to assess the impact of ribociclib (LEE-011) used as pre-treatment or concurrent with cisplatin are in progress.

**Figure 4. LEE-011 decreases ovarian cancer growth in murine xenograft model**

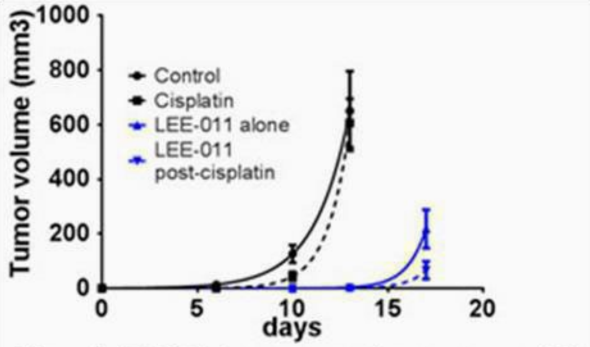


Figure 4. LEE-011 decreases ovarian cancer growth in a murine xenograft model. Mice were treated with cisplatin alone, LEE-011 alone daily, or cisplatin followed by LEE-011 daily and tumor size was followed until prespecified euthanasia criteria was met.

We propose using ribociclib (LEE-011) 1) as concurrent therapy with platinum-based chemotherapy in platinum-sensitive recurrent ovarian cancer and 2) as maintenance therapy after completion of platinum-based chemotherapy in platinum-sensitive recurrent ovarian cancer. <u>We hypothesize that concurrent ribociclib (LEE-011) treatment and chemotherapy will enhance the response to platinum-based therapy and maintenance therapy will slow ovarian cancer tumor growth leading to prolongation in progression free survival.</u>

## 1.5 Correlative Studies

As mentioned above, CDK4 and CDK6 work through modulation of the Rb protein therefore non-functional Rb protein will convey resistance to CDK4/6 inhibition. In ovarian cancer, based on TCGA data, ~2% of tumors have Rb deep deletions (leading to complete loss of Rb) while an additional ~16% have missense mutations or truncation mutations (with unclear effects on Rb expression and functionality) [12]. We will therefore assess Rb mutational status in patient tumor samples to determine correlation with ribociclib (LEE-011) treatment outcomes. Additionally, as NFAT4 is an innate CDK4/6 inhibitor, we will stain tumor samples for NFAT4 nuclear localization (which represents active NFAT4) to determine correlation with ribociclib (LEE-011)

(hypothesizing that low nuclear NFAT4 localization will be correlated with increased response to ribociclib (LEE-011)).

## 2.0 STUDY OBJECTIVES

### 2.1 Primary Objectives

2.1.1 To determine the MTD of ribociclib (LEE-011) with platinum + taxane chemotherapy in platinum-sensitive recurrent ovarian cancer.

### 2.2 Secondary Objectives

2.2.1 Overall Survival (OS)

2.2.2 To observe and record anti-tumor activity. Although the clinical benefit of this drug has not yet been established, the intent of offering this treatment is to provide a possible therapeutic benefit, and thus the patient will be carefully monitored for tumor response and symptom relief in addition to safety and tolerability.

2.2.3 Determine the RR and PFS of ribociclib (LEE-011) with platinum + taxane followed by maintenance ribociclib (LEE-011).

2.2.4 Further characterize the safety profile of ribociclib (LEE-011) with platinum + taxane in recurrent platinum-sensitive ovarian cancer.

2.2.5 To correlate RR and PFS with Rb mutational status and NFAT4 nuclear localization levels.

### 2.3 Primary Endpoints

2.3.1 Occurrence of DLTs in each patient with assessment of AEs/SAEs per CTCAE v5 in the first 2 cycles

### 2.4 Secondary Endpoints

2.4.1 ORR and PFS as per GCIG-RECIST

2.4.2 Tolerability as measured by history & physical exam and laboratory assessment

### 2.5 Correlative/Exploratory Endpoints

2.5.1 Determine level of association between baseline Rb mutational status as measured via PCR and NFAT nuclear expression via IHC from frozen or paraffin embedded tissue obtained from initial tissue diagnosis, debulking surgery or biopsy samples with RR and PFS clinical endpoints.

## 3.0 PATIENT ELIGIBILITY

Subjects must meet all of the inclusion and exclusion criteria to be enrolled to the study. Study treatment may not begin until a subject is enrolled.

### 3.1 Inclusion Criteria

1. Women ≥18 years old with platinum-sensitive recurrent ovarian, fallopian or primary peritoneal cancer (defined as recurrent disease >6 months after completing last platinum-based chemotherapy) eligible to receive platinum-based doublet chemotherapy.
2. Must have had at least 1 prior line of platinum-based therapy
3. ECOG 0-1 with life expectancy of ≥ 3months
4. Adequate organ function:

- Serum creatinine ≤1.5mg/dL or 24-hour clearance≥50mL/min
- AST/ALT <2.5x ULN (or <5x ULN if liver metastasis are present)
- Total bilirubin ≤ ULN or total bilirubin ≤3.0 x ULN or directbilirubin ≤1.5 x ULN in patients with well-documented Gilbert's Syndrome
- Hemoglobin ≥9gm/dl, Platelets ≥100,000/µl ANC ≥1500/µl
- INR ≤1.5
- Potassium, total calcium (corrected for serum albumin), magnesium, and sodium within normal limits for the institution or corrected to within normal limits with supplements before first dose of study medication

5. Screening ECG (defined as the mean of the triplicate ECGs) with QTcF interval at screening≤450msec (using Fridericia's correction) andrestingheart rate 50-90bpm
6. Must be able to swallow ribociclib (LEE-011)tablet/capsule
7. Documented disease recurrence/progression based onGCIG-RECIST
8. Able to provide informed consent and comply with all study protocols
9. Treated CNS metastasis allowed if treatment is completed ≥8 weeks prior to enrollment. Patients must be asymptomatic off systemic corticosteroids for at least 4 weeks after completion of radiation therapy. CNS disease must be stable or regressed on repeat imaging performed at least 4 weeks after completion oftherapy.

10. Women of child-bearing potential (those who have had a menstrual cycle within the last year and have not had a tubal ligation or surgical removal of both ovaries and/or hysterectomy) must agree to abstain from vaginal intercourse of use and continue highly effective methods of contraception for 3 weeks after discontinuation of study treatment.
    - Total abstinence when this is in line with the preferred and usual lifestyle of the patient. Periodic abstinence (e.g., calendar, ovulation, symptothermal, post-ovulation methods) and withdrawal are not acceptable methods ofcontraception
    - Female sterilization (have had surgical bilateral oophorectomy with or without hysterectomy), total hysterectomy, or tubal ligation at least six weeks before taking study treatment. In case of oophorectomy alone, only when the reproductive statusof the woman has been confirmed by follow up hormone level assessment
    - Male sterilization (at least 6 months prior to screening). The vasectomized male partner should be the sole partner for thatpatient
    - Use of oral, injected or implanted hormonal methods of contraception or placement of an intrauterine device (IUD) or intrauterine system (IUS), or other forms of hormonal contraception that have comparable efficacy (failure rate <1%), for example hormone vaginal ring or transdermal hormone contraception.
    - In case of use of oral contraception, women should have been stable on the same pill for a minimum of 3 months before taking study treatment.

**Note:** Oral contraceptives are allowed but should be used in conjunction with a barrier method of contraception due to unknown effect of drug-drug interaction.

Women are considered post-menopausal and not of child bearing potential if they have had 12 months of natural (spontaneous) amenorrhea with an appropriate clinical profile (e.g. age appropriate, history of vasomotor symptoms) or have had surgical bilateral oophorectomy (with or without hysterectomy) or tubal ligation at least six weeks ago. In the case of oophorectomy alone, only when the reproductive status of the woman has been confirmed by follow up hormone level assessment is she considered not of child bearing potential.]

11. Patients must have histologically confirmed malignancy that is metastatic or unresectable and for which standard curative or palliative measures do not exist or are no longer effective.

**3.2 Exclusion Criteria**

1. Borderline or low-malignant potential histology.
2. Platinum-resistant disease (as defined as progressive disease within 6 months of completion of chemotherapy with a platinum agent)
3. Grade 3 baseline neuropathy.
4. Known hypersensitivity to any of the excipients of ribociclib (LEE-011)including peanuts and soy
5. Prior use of CDK4/6 inhibitors.
6. Congenital long QT syndrome or family history of unexpected sudden cardiac death
7. Concurrent malignancy or malignancy within 3 years prior to starting study drug, with the exception of adequately treated basal or squamous cell carcinoma, non-melanomatous skin cancer or curatively resected cervical cancer or per physician discretion that the previous cancer was adequately treated with curative intent and unlikely to recur (the study PI must concur with this determination)
8. Gastrointestinal (GI) function or disease that may significantly alter the absorption of the study drugs
9. History of HIV infection
10. Patient has any other concurrent severe and/or uncontrolled medical condition that would, in the investigator's judgment, cause unacceptable safety risks and contraindicate patient's participation in the clinical study or compromise compliance with the protocol (e.g. chronic pancreatitis, chronic active hepatitis, active untreated or uncontrolled fungal, bacterial or viral infections, etc.).
11. Clinically significant, uncontrolled heart disease and/or cardiac repolarization abnormalities, including any of the following:
    a. Heart Association functional classification III-IV)
    b. Documented cardiomyopathy
    c. Left Ventricular Ejection Fraction (LVEF) <50% as determined by Multiple Gated acquisition (MUGA) scan or echocardiogram (ECHO)at screening
    d. Clinically significant cardiac arrhythmias (e.g. ventricular tachycardia), complete left bundle branch block, high-grade AV block (e.g. bifascicular block, Mobitz type II and third-degree AV block)
    e. Long QT syndrome or family history of idiopathic sudden death or congenital long QT syndrome, or any of the following:
        i. Risk factors for Torsades de Pointe (TdP) including uncorrected hypokalemia or hypomagnesaemia, history of cardiac failure, or history of clinically significant/symptomatic bradycardia.
        ii. Concomitant use of medication(s) with a known risk to prolong the QT interval and/or known to cause Torsades dePointe that cannot be discontinued (within 5 half-lives or 7 days prior to starting study drug) or replaced by safe alternative medication

iii. Inability to determine the QT interval on screening (QTcF using Fridericia's correction)

e. Systolic blood pressure (SBP) >160 mmHg or <90 mmHg at screening
f. History of acute coronary syndromes (including myocardial infarction, unstable angina, coronary artery bypass grafting, coronary angioplasty, or stenting) or symptomatic pericarditis within 6 months prior to screening

12. Use of prohibited medications (see section 5.3) that cannot be changed to an alternative therapy
13. Patient is currently receiving or has received systemic corticosteroids ≤2 weeks prior to starting study drug, or who have not fully recovered from side effects of such treatment.
    a. The following uses of corticosteroids are permitted: single doses, topical applications (e.g., for rash), inhaled sprays (e.g., for obstructive airways diseases), eye drops or local injections (e.g., intra-articular)
14. Patient is currently receiving warfarin or other coumadin-derived anticoagulant for treatment, prophylaxis or otherwise. Therapy with heparin, low molecular weight heparin (LMWH) or fondaparinux is allowed.
15. Use of herbal supplements unless discontinued ≥7 days prior to initiation of study drug
16. Consumption of foods which are strong inducers or inhibitors of CYP3A4/5 has to be discontinued 7 days prior to initiation of study drug
17. Pregnancy or lactation
18. Participation in a prior investigational study within 30 days prior to enrollment or within 5 half-lives of the investigational product, whichever is longer
19. Patient who has received radiotherapy ≤4 weeks or limited field radiation for palliation ≤2 weeks prior to starting study drug, and who has not recovered to grade 1 or better from related side effects of such therapy (exceptions include alopecia) and/or in whom ≥25% of the bone marrow (Ellis, 1961) was irradiated.
20. Patient has had major surgery within 14 days prior to starting study drug or has not recovered from major side effects (tumor biopsy is not considered as major surgery).
21. Patient has not recovered from all toxicities related to prior anticancer therapies to NCI-CTCAE version 5 Grade ≤1 (Exception to this criterion: patients with any grade of alopecia and/or neuropathy ≤ grade 2 are allowed to enter the study).
22. Patient with a Child-Pugh score B or C.

## 4.0 SUBJECT SCREENING AND REGISTRATION PROCEDURES

Subjects will be identified in clinic. After informed consent is obtained and PRIOR to the initiation of protocol therapy all patients satisfying the inclusion/exclusion criteria must have eligibility confirmed by the Clinical Trials Office. The patient will not be considered registered and enrolled in the study until all information is confirmed by the Clinical Trials Office Data Manager.

## 5.0 Treatment Dosage and Administration

Protocol treatment must start within 15 business days of enrollment to the study. Women with recurrent platinum-sensitive ovarian cancer clinically eligible to receive platinum-based doublet chemotherapy will be enrolled prior to starting chemotherapy.

### 5.0.1 Ribociclib (LEE-011) treatment with platinum + taxane chemotherapy

- Women will be given ribociclib (LEE-011) on days 1-4, 8-11, and 15-18 with weekly carboplatin + paclitaxel (given days 1, 8, and 15 of a 28 day cycle)for6 total cycles of chemotherapy. Ribociclib (LEE-011) dose will be determined through TITE-CRM doseescalation.
  - Ribociclib (LEE-011) will be administered orally
  - Carboplatin and paclitaxel will be administered via IV weeklydays1,8 and 15 of a 28 day cycle as per standard dosing.

For patients with an intolerance/allergy to taxol, weekly docetaxel (30 mg/m2) can be used.
For patients with an intolerance/allergy to carboplatin, weekly cisplatin (35 mg/m2) can be used.

- Blood count, renal and hepatic function and ECG (ECG only with the first 2 cycles) monitoring will be performed prior to carboplatin + paclitaxel + ribociclib (LEE-011) dosing (days 1,8, and 15).

### 5.0.2 Dose-escalation and dose-limiting toxicity

- Dose-limiting toxicity (DLT) is defined as any grade 3–4 non-hematological or grade 4 hematological toxicity at least possibly related to the treatment, occurring during the first two cycles of treatment. Per common terminology criteria for adverse events (CTCAE) v5.
- In the event that the patient receives ribociclib (LEE-011) but does not start carboplatin + paclitaxel, they will not be used to assess DLT.

**Table 1. DLT Criteria**

| **Toxicity** | **DLT Criteria** |
|---|---|
| Hematology | CTCAE grade 4 neutropenia<br>CTCAE grade 4 thrombocytopenia<br>CTCAE grade 4 lymphopenia<br>CTCAE grade 3 thrombocytopenia with bleeding<br>CTCAE grade 3 or 4 febrile neutropenia |
| ECG QT interval | QTcF interval ≥481 ms on at least 2 separate ECGs (calculated from the mean of triplicate ECGs) |
| Cardiac | Cardiac toxicity ≥ CTCAE grade 3<br>Clinical signs of cardiac disease, such as unstable angina or myocardial infarction, or Troponin ≥ CTCAE grade 3 |
| Gastro-intestinal | ≥ CTCAE grade 3 vomiting ≥48 hours despite optimal anti-emetic therapy<br>≥ CTCAE grade 3 diarrhea ≥48 hours despite optimal anti-diarrhea treatment |
| Hepato-biliary | ≥ CTCAE grade 2 total bilirubin for more than 7 consecutive days<br>≥ CTCAE grade 3 total bilirubin<br>≥ CTCAE grade 2 ALT with a ≥grade 2 bilirubin elevation of any duration in the absence of liver metastases<br>≥ CTCAE grade 3 ALT for >4 consecutive days<br>CTCAE grade 4 ALT or AST<br>Grade 4 serum alkaline phosphatase >7 consecutive days |
| Renal | ≥ CTCAE grade ≥3 serum creatinine |
| Non-hematologic events | ≥ CTCAE grade 3, except for the exclusions noted below: |
| Exceptions to DLT criteria | Grade 3 alopecia<br><5 days of CTCAE grade 3 fatigue<br>Grade 3 fever or infection without neutropenia <5 days duration<br>Grade 3 laboratory abnormalities that are responsive to oral supplementation or deemed by the investigator to be clinically insignificant |
| CTCAE version 5 should be used for grading.<br>Optimal therapy for vomiting and diarrhea should be based on institutional guidelines with consideration of the prohibited medications listed in these protocol guidelines. | |

**Table 2. Dose-Escalation Criteria**

| Dose-Escalation Schedule | | | |
|---|---|---|---|
| **Dose level** | **Dose of study agent: ribociclib (LEE-011)** | **Dose of standard agent: Carboplatin[a]** | **Dose of standard agent: Paclitaxel[b]** |
| Level 1 ** | 200 mg/d | AUC 2 | 60 mg/m2 |
| Level 2 | 400 mg/d | AUC 2 | 60 mg/m2 |
| Level 3 | 600 mg/d | AUC 2 | 60 mg/m2 |
| ** starting dose level | | | |
| a: For patients allergic to carboplatin, weekly cisplatin 35mg/m2 can be used | | | |
| b: For patients allergic/intolerant of Paclitaxel, docetaxel (30mg/m2) can be used | | | |

### 5.0.3 Ribociclib (LEE-011) maintenance after completion of chemotherapy

- Women who achieve at least a PR (as assessed after completion of chemotherapy) will continue on maintenance ribociclib (LEE-011) at 600mg/day 3 weeks on, 1 week off (± 2 days) until time of progression. Maintenance dose is based on previous phase 1 determine RP2D for ribociclib (LEE-011) maintenance therapy [7].
- Patients who are unable to complete 6 cycles of chemotherapy due to chemotherapy related toxicity but have achieved at least a PR will still be eligible for Ribociclib maintenance therapy. If the treating clinician feels chemotherapy treatment should be discontinued due to chemotherapy related toxicity, a CT scan should be obtained 4 weeks (± 2 days) of the last dose of chemotherapy. Ribociclib maintenance should be started within 6 weeks of completion of chemotherapy.
- To start maintenance therapy, the patient must meet the following criteria: ANC ≥1 x $10^9$/L, hemoglobin ≥9 g/dL and platelets ≥75 x $10^9$/L. Initiation of maintenance therapy may be delayed for hematologic count recovery but must start within 6 weeks from last dose of carboplatin + paclitaxel or patient will not be eligible for maintenance therapy.
- Study assessment will be performed 4 weeks (±2 days) prior to the start of each ribociclib (LEE-011) maintenance cycle (day 1 of ribociclib (LEE-011) maintenance cycle). Weekly telephone toxicity checks by the research team will be performed for the first 2 cycles of maintenance then biweekly for the next 2 cycles of maintenance. Interim labs every 2 weeks (CBCD, BMP) will be performed for the first 4 cycles of maintenancetherapy.
- Each actively enrolled subject will continue to be contacted weekly (via phone, video, or office visit) to address study drug use and diary maintenance, stress the importance of not starting any new medications without checking first with study staff, and to address any new consent forms or protocol changes.

- Response will be monitored with serial CA125 measurements and Q3 cycle CT scans during chemotherapy.
- Progression will be assessed per GCIG-RECIST criteria. CA125 values will be monitored monthly during the maintenance portion and CT imaging will be repeated Q3 months (± 1 week) to assess for disease progression. Patientswill

continue on therapy until time of progression or unacceptable toxicity.

## 5.1 Toxicities and Dosing Delays/Dose Modifications

- Any patient who receives treatment on this protocol will be evaluable for toxicity. Each patient will be assessed for the development of toxicity according to the Time and Events Table (Section 6.4). Toxicity will be assessed according to the NCI Common Terminology Criteria for Adverse Events (CTCAE), version 5. Dose escalation/de-escalation will be determined based on rate of DLTs via TITE-CRM algorithm.
- Dose modifications in the study are outlined below.
- If toxicities are clearly related to carboplatin + paclitaxel, dose reductions are allowed per standard of care.

- Following significant allergic reactions to carboplatin or paclitaxel during study, a standard desensitization protocol is allowed with next cycle of therapy.

**Table 3. Hematological Toxicity Dose Reductions for ribociclib (LEE-011)**

| Toxicity/Grade | Dose Adjustment and Management Recommendations |
|---|---|
| **Thrombocytopenia** | |
| Grade 1 (≥75 x $10^9$/L) | No dose adjustment required. |
| Grade 2 (≥50 x $10^9$/L – <75 x $10^9$/L) | Dose interruption until recovery to grade ≤1. Re-initiate ribociclib (LEE-011) at the same dose. |
| Grade 3 (≥25 x $10^9$/L - <50 x $10^9$/L) | Dose interruption until recovery to grade ≤1. Re-initiate ribociclib (LEE-011) at the same dose level. If toxicity recurs at grade 3: temporary dose interruption until recovery to grade ≤1 and reduce ribociclib (LEE-011) to the next lower dose level. |
| Grade 4 (<25 x $10^9$/L) | Dose interruption until recovery to grade ≤1. Re-initiate ribociclib (LEE-011) at the next lower dose level. If toxicity recurs at grade 4: discontinue ribociclib (LEE-011) |
| **Absolute neutrophil count (ANC)** | |
| Grade 1 (≥1.5 x $10^9$/L) | No dose adjustment required. |
| Grade 2 (≥1.0 - <1.5 x $10^9$/L) | No dose adjustment required. |
| Grade 3 (≥0.5 - <1.0 x $10^9$/L) | Dose interruption until recovery to ≥1.0 x $10^9$/L. Re-initiate ribociclib (LEE-011) at the same dose level. If toxicity recurs at grade 3: temporary dose interruption until recovery to ≥1.0 x $10^9$/L then reduce ribociclib (LEE-011) dose to the next lower dose level. |
| Grade 4 (<0.5 x $10^9$/L) | Dose interruption until recovery to ≥1.0 x $10^9$/L. Re-initiate ribociclib (LEE-011) at the next lower dose level. If toxicity recurs at grade 4: temporary dose interruption until recoveryto ≥1.0 x $10^9$/L and reduce ribociclib (LEE-011) at the next lower dose level. |
| **Febrile neutropenia** | |
| Grade 3 ANC <1.0 x $10^9$/L with a single temperature of >38.3 ºC (101 ºF) or a sustained temperature of ≥38 ºC (100.4 ºF) for more than one hour | Dose interruption until improvement of ANC ≥1.0 x $10^9$/L and no fever. Restart at the next lower dose level. If febrile neutropenia recurs, discontinue ribociclib(LEE-011). |
| Grade 4 Life-threatening consequences; urgent intervention indicated | Discontinue ribociclib (LEE-011). |
| **Anemia (Hemoglobin)** | |
| Grade 1 (≥10.0 – LLN g/dL) | No dose adjustment required. |
| Grade 2 (≥8.0 – <10.0 g/dL) | No dose adjustment required. |
| Grade 3 (<8.0 g/dL) | Dose interruption until recovery to grade ≤2. Re- initiate ribociclib (LEE-011) at the same dose. |

| | |
|---|---|
| Grade 4 Life-threatening consequences; urgent intervention indicated | Discontinue ribociclib (LEE-011). |
| **Other hematologic toxicity** | |
| Grade 1 | No dose adjustment required. |
| Grade 2 | No dose adjustment required. |
| Grade 3 | No dose adjustment required |
| Grade 4 | Dose interruption until recovery to grade 2 or lower. Re-initiate ribociclib (LEE-011) at the next lower dose level. If toxicity recurs at grade 4 after maximum dose reduction discontinue ribociclib |

| **HEPATOTOXICITY (BILIRUBIN, SGPT/ALT, SGOT/AST)** | |
|---|---|
| **TOTAL BILIRUBIN without ALT/AST increase above baseline value** | |
| Grade 1 (> ULN – 1.5 x ULN) (confirmed within 24 hours) | Maintain dose level with LFTs monitored bi-weekly |
| Grade 2 (>1.5 – 3.0 x ULN) | Dose interruption of ribociclib (LEE-011)<br>If resolved to ≤ grade 1 in ≤21 days, then maintain dose level<br>If resolved to ≤ grade 1 in >21 days or toxicity recurs, then reduce 1 dose level<br>Repeat liver enzymes and bilirubin tests twice weekly for 2 weeks after dose resumption<br>If toxicity recurs after two dose reductions, discontinue ribociclib (LEE-011) |
| Grade 3 (>3.0 – 10.0 x ULN) | Dose interruption of ribociclib (LEE-011)<br>If resolved to ≤ grade 1 in ≤21 days, lower 1 dose level of ribociclib (LEE-011)<br>Repeat liver enzymes and bilirubin tests twice weekly for 2 weeks after dose resumption<br>If resolved to ≤ grade 1 in >21 days or toxicity recurs, discontinue ribociclib (LEE-011) |
| Grade 4 (>10.0 x ULN) | Discontinue ribociclib (LEE-011) |
| Confounding factors and/or alternative causes for increase of total bilirubin should be excluded before dose interruption/reduction. They include but are not limited to: evidence of obstruction, such as elevated ALP and GGT typical of gall bladder or bile duct disease, hyperbilirubinemia due to the indirect component only (i.e. direct bilirubin component ≤1 x ULN) due to hemolysis or Gilbert Syndrome, pharmacologic treatment, viral hepatitis, alcoholic or autoimmune hepatitis, other hepatotoxic drugs.<br>For patients with Gilbert Syndrome, these dose modifications apply to changes in direct bilirubin only. Bilirubin will be fractionated if elevated. | |

**Table 4. Hepatotoxicity Dose Reductions for ribociclib (LEE-011)**

| | |
|---|---|
| **AST or ALT without bilirubin elevation >2 x ULN** | |
| Same grade as baseline or increase from baseline grade 0 to grade 1<br>(confirmed within 24hrs) | No dose adjustment required with LFTs monitored per protocol if same grade as baseline or bi-weekly in case of increase from baseline grade 0 to 1 |
| Increase from baseline grade 0 or 1 to grade 2<br>(>3.0 – 5.0 x ULN) | Dose interruption of ribociclib (LEE-011)<br>If resolved to ≤ baseline grade in ≤21 days, then maintain dose level<br>If resolved to ≤ baseline grade in >21 days or toxicity recurs, then reduce 1 dose level<br>Repeat liver enzymes and bilirubin tests twice weekly for 2 weeks after dose resumption<br>If toxicity recurs after two dose reductions or recovery to ≤ baseline grade is >28 days, discontinue ribociclib (LEE-011) |
| Increase from baseline grade 0 or 1 to grade 3<br>(>5.0 – 20.0 x ULN) | Dose interruption of ribociclib (LEE-011) until resolved to ≤ baseline grade, then lower 1 dose level of ribociclib (LEE-011)<br>Repeat liver enzymes and bilirubin tests twice weekly for 2 weeks after dose resumption<br>If recovery to ≤ baseline grade is >28 days, discontinue ribociclib (LEE-011)<br>If toxicity recurs, discontinue ribociclib (LEE-011) |
| Increase from baseline grade 2 to grade 3 (>5.0 – 20.0 x ULN) | Dose interruption of ribociclib (LEE-011) until resolved to < baseline grade, then lower 1 dose level of ribociclib (LEE-011)<br>Repeat liver enzymes and bilirubin tests twice weekly for 2 weeks after dose resumption<br>If toxicity reoccurs after 2 dose reductions or recovery to < baseline grade is > 28 days, discontinue ribociclib (LEE-011) |
| Grade 4 (>20.0 x ULN) | Discontinue ribociclib (LEE-011) |
| **AST or ALT and concurrent Bilirubin** | |
| For patients with normal ALT or AST or total bilirubin at baseline: AST or ALT ≥ grade 2 combined with total bilirubin >2 x ULN without evidence of cholestasis<br>**or**<br>For patient with elevated AST or ALT or total bilirubin at baseline: baseline: [AST or ALT >2 x baseline AND >3.0 x ULN] OR [AST or ALT 8.0 x ULN]- whichever is lower-combined with [total bilirubin 2 x baseline AND >2.0 x ULN]<br>Where whichever is lower- combined with [total bilirubin 2 x baseline AND >2.0 x ULN] | Discontinue ribociclib (LEE-011) |
| Confounding factors and/or alternative causes for increased transaminases should be excluded before dose interruption/reduction. They include but are not limited to: concomitant medications, herbal preparations or dietary supplements, infection, hepato-biliary disorder or obstruction, new or progressive liver metastasis, and alcohol intake. | |

Increase in transaminases combined with total bilirubin (TBIL) increase may be indicative of drug-induced liver injury (DILI), and should be considered as clinically important events.

The threshold for potential DILI may depend on the patient's baseline AST/ALT and TBIL value; patients meeting any of the following criteria will require further follow-up as outlined below:

- For patients with normal ALT or AST or TBIL value at baseline: AST orALT > 3.0 x ULN combined with TBIL > 2.0 x ULN

- For patients with elevated AST or ALT or TBIL value at baseline: [AST or ALT > 2 x baseline AND > 3.0 x ULN] OR [AST or ALT > 8.0 x ULN], whichever is lower, combined with [TBIL > 2 x baseline AND > 2.0 x ULN]

Medical review needs to ensure that liver test elevations are not caused by cholestasis, defined as: ALP elevation > 2.0 x ULN with R value < 2 in patients without bone metastasis, or elevation of ALP liver fraction in patients with bone metastasis. Note: (The R value is calculated by dividing the ALT by the ALP, using multiples of the ULN for both values. It denotes the relative pattern of ALT and/or ALP elevation is due to cholestatic or hepatocellular liver injury or mixed type injury).

In the absence of cholestasis, these patients should be immediately discontinued from study drug treatment, and repeat LFT testing as soon as possible, within 24 hours from the awareness of the abnormal results. The evaluation should include laboratory tests, detailed history, physical assessment and the possibility of liver metastasis or new liver lesions, obstructions/compressions, etc.
Hepatic toxicity monitoring includes the following LFTs: albumin, ALT, AST, total bilirubin, direct and indirect bilirubin, alkaline phosphatase (fractionated if alkaline phosphatase is grade 2 or higher), creatinine kinase, prothrombin time (PT/INR) and GGT.

For patients with Gilbert Syndrome: total and direct bilirubin must be monitored, intensified monitoring applies to changes in direct bilirubin only.

Close observation is recommended in case of AST, ALT, and/or bilirubin increase requiring dose interruption, which involves:

- Repeating liver enzyme and serum bilirubin tests two or three times weekly. Frequency of re-testing can decrease to once a week or less if abnormalities stabilize or return to normal values.

- Obtaining a more detailed history of currentsymptoms.

- Obtaining a more detailed history of prior and/or concurrent diseases, including history of any pre-existing liver conditions or riskfactors.

- Obtaining a history of concomitant drug use (including non-prescription medications, herbal and dietary supplements), alcohol use, recreational druguse,

and special diets.

• Ruling out acute viral hepatitis types A, B, C, D, and E; hepatotropic virus infections (CMV, EBV or HSV); autoimmune or alcoholic hepatitis; NASH; hypoxic/ischemic hepatopathy; and biliary tract disease.

• Obtaining a history of exposure to environmental chemical agents.

• Obtaining additional tests to evaluate liver function, as appropriate (e.g., INR, direct bilirubin).

• Considering gastroenterology or hepatology consultations.

• Assessing cardiovascular dysfunction or impaired liver oxygenation, including hypotension or right heart failure as possible etiologies for liverdysfunction.

• Liver biopsy as clinically indicated to assess pathological changeand degree of potential liver injury.

All cases confirmed on repeat testing meeting the laboratory criteria defined above, with no other alternative cause for LFT abnormalities identified, should be considered as “medically significant”, thus met the definition of SAE and reported as SAE using the term “potential drug-induced liver injury”. All events should be followed up with the outcome clearly documented.

**Table 5. Dose modification for QTcprolongation**

| **Grade** | **Dose Modification** |
|---|---|
| For all grades | • Check the quality of the ECG and the QT value and repeat if needed (QTc shouldbe the average of triplicate measurements).<br>• Perform analysis of serum electrolytes (K+, Ca++, Phos, Mg++). If below the lower limit of normal, interrupt ribociclib (LEE-011) administration, correct with supplements or appropriate therapy as soon as possible, and repeat electrolytes until documented as normal.<br>• Review concomitant medication usage for the potential to inhibitCYP3A4 and/or to prolong the QTinterval.<br>• Check compliance with correct dose and administration of ribociclib (LEE- 011). |
| 1<br>QTc 450-480 ms | Perform steps 1-4 as directed in "For All Grades." No dose adjustment required. |
| 2<br>QTc 481-500 ms | Interrupt ribociclib (LEE-011). Perform steps 1-4 as directed in "For All Grades."<br>• Perform a repeat ECG within one hour of the first QTcFof≥481ms<br>• Repeat ECG as clinically indicated until the QTcF returns to <481ms. Restart ribociclib (LEE-011) with dose reduced by 1 dose level (please refer to the dosing schedule table).<br>• If QTcF ≥481 ms recurs, ribociclib (LEE-011) should be reduced by 1 dose level (please refer to the dosing scheduletable)<br>• Repeat ECGs 7 days and 14 days after dose resumption (then as clinically indicated) for any patient who has therapy interrupted due to QTcF ≥481 ms |
| 3<br>QTc ≥501 ms on at least two separate ECGs | Interrupt ribociclib (LEE-011). Perform steps 1-4 as directed in "For All Grades."<br>• Perform a repeat ECG within one hour of the first QTcF of≥501ms.<br>• If QTcF remains ≥501 ms, consult with a cardiologist (or qualified specialist) and repeat cardiac monitoring as indicated until the QTcF returns to <481 ms.<br>• If QTcF returns to <481 ms, ribociclib (LEE-011) should be reduced by 1 dose level (please refer to the dosing schedule table) |
| | • If QTcF remains ≥481 after performing steps 1-4 as directed in "For All Grades," discontinue ribociclib<br>• Repeat ECGs 7 days and 14 days after dose resumption for any patient who has therapy interrupted due to QTcF ≥501ms<br>• If QTcF of ≥501 ms recurs, discontinue ribociclib(LEE-011) |
| 4<br>QT/QTc ≥501 or >60 ms change from baseline and Torsades de pointes or polymorphic ventricular tachycardia, or signs/symptoms of serious arrhythmia | Discontinue ribociclib (LEE-011). Perform steps 1-4 as directed in "For all Grades"<br>• Obtain local cardiologist consultation (or qualified specialist) and repeat cardiac monitoring as indicated until the QtcF returns to<481ms |

**Table 6. Dose Modification and Management for Interstitial Lung Disease/Pneumonitis**

| Grade | Dose modification |
|---|---|
| Grade 1 (asymptomatic) | No dose interruption or adjustment is required. Initiate appropriate medical therapy and monitor as clinically indicated |
| Grade 2 (symptomatic) | Dose interruption until recovery to Grade ≤1 then resume ribociclib at the next lower dose level. If grade 2 recurs, discontinue ribociclib |
| Grade 3 (sever symptomatic) or Grade 4 (life threatening) | Discontinue ribociclib |

**Table 7. Ribociclib (LEE-011) Dose Adjustment and Management Recommendation for All Other Adverse Reactions**

| **Grade** | **Dose Adjustment and Management Recommendations** |
|---|---|
| 1 | No dose adjustment recommended. Initiate appropriate medical therapy and monitor. |
| 2 | Dose interruption until recovery to grade ≤1. Initiate appropriate medical therapy and monitor.<br>Re-initiate ribociclib (LEE-011) at the same dose.<br>• If the same toxicity recurs at grade 2, interrupt ribociclib (LEE-011) until recovery to grade ≤1. Re-initiate ribociclib (LEE-011) at the next lower dose level. |
| 3 | Dose interruption until recovery to grade ≤1. Initiate appropriate medical therapy and monitor.<br>Re-initiate ribociclib (LEE-011) at the next lower dose level.<br>• If toxicity recurs at grade 2: temporary dose interruption until recovery to grade ≤1 and reduce ribociclib (LEE-011) dose the next lower dose level.<br>• If toxicity recurs at grade 3, discontinue ribociclib(LEE-011). |
| 4 | Discontinue ribociclib (LEE-011) and treat with appropriate medical therapy. |

**If ribociclib was dose reduced or stopped during the combined ribociclib and chemotherapy treatment period and the toxicity leading to dose reduction or discontinuation has resolved to grade 1 or better (or grade 2 or better for hematologic toxicity), ok to start ribociclib maintenance at the 600mg dose as long as the patient meets hematologic parameters for starting maintenance as outlined above.**

**5.2 Concomitant Medications/Treatments**

Permitted concomitant therapy: Medications required to treat AEs, manage cancer symptoms, concurrent diseases and supportive care agents, such as pain medications, anti-emetics and anti-diarrheal are allowed.

The patient must be told to notify the investigational site about any new medications she takes after the start of the study treatment. All medications (other than study drugs) and significant non- drug therapies

(including vitamins, herbal medications, physical therapy and blood transfusions) administered within 30 days of study entry and during the study must be listed on the Concomitant medications/Significant non-drug therapies section of the patient record.

Bisphosphonates and denosumab are permitted for the treatment of osteoporosis and prevention of skeletal related events for patients with bone metastases. Chronic concomitant bisphosphonate/denosumab therapy for the prevention of bone metastasis is not permitted.

Strong inhibitors or inducers of CYP3A4/5 must be discontinued at least 7 days prior to starting study drug.

Hematopoietic growth factors may be used according to ASCO guidelines.

Palliative radiation is permitted if done solely for bone pain relief. It should not be delivered to a target lesion and it should not encompass more than 25% of irradiated bone marrow. No dose modification of study treatment is needed during palliative radiotherapy.

Refer to the ribociclib (LEE-011) Investigator's Brochure, Table 1, and Table 2 for information on possible interactions with other drugs.

Permitted concomitant therapy requiring caution: Medications to be used with caution during ribociclib (LEE-011) in this study are listed below. This list is not comprehensive and is only meant to be used as a guide. These medications should be excluded from patient use if possible. If they must be given, then use with caution and consider a ribociclib (LEE-011) interruption if the concomitant medication is only needed for a short time.

- Moderate inhibitors or inducers ofCYP3A4/5
- Sensitive substrates of CYP3A4/5 that do not have narrow therapeutic index
- Strong inhibitors of BSEP
- Sensitive substrates of the renal transporters, MATE1andOCT2
- Sensitive substrates of BCRP
- Medications that carry a possible risk for QTprolongation

**Table 8. Permitted concomitant therapy requiring caution**

| Category | Drug Name |
|---|---|
| Moderate CYP3A4/5 inhibitors | Aprepitant, amprenavir, asafoetida resin (Ferula asafoetida) cimetidine, crizotinib, diltiazem, faldaprevir, imatinib, isavuconazole, netupitant, nilotinib, tofisopam, Schisandra sphenanthera (nan wu wei zi), verapamil |
| Moderate CYP3A4/5 inducers | Bosentan, dabrafenib, efavirenz, etravirine, genistein,lopinavir[5], modafinil, nafcillin,telotristat |
| Sensitive CYP3A4/5 substrates[1] | Alpha-dihydroergocryptine, aprepitant, atorvastatin, avanafil, bosutinib,brotizolam, budesonide, buspirone, cobimetinib, darifenacin, dasatininb, ebastine, eletriptan, eplerenone, everolimus, felodipine, fluticasone, grazoprevir, ibrutnib, isavuconazole, ivabradine, ivacaftor, , levomethadyl (LAAM), lomitapide, lovastatin, lumefantrine, lurasidone, maraviroc, midazolam, midostaurin, naloxegol, neratinib, nisoldipine, perospirone, quetiapine, ridaforolimus, sildenafil, simeprevir, simvastatin, ticagrelor, tilidine, tolvaptan, triazolam, ulipristal, vardenafil, venetoclax, vicriviroc, voclosporin |
| BSEP inhibitors | Alectinib, atorvastatin, bromocriptine, candesartan, clobetasol, clofaziminie, dabigatran, dipyridamole, glyburide, grazoprevir, ledipasvir, mifepristone,pioglitazone, reserpine,rifamycin, simeprevir, telmisartan,timcodar, troglitazone, valinomycin, velpatasvir |
| Medications that carry a possible risk for QT prolongation[2] | Alfuzosin, apomorphine, aripiprazole, artenimol+piperaquine,asenapine, , atomoxetine, bedaquiline, bendamustine, bortezomib, bosutinib, buprenorphine, cabozantinib, capecitabine, ceritinib, clomipramine, crizotinib,clozapine, cyamemazine (cyamepromazine), dabrafenib, dasatinib, degarilix, delamanid, desipramine, dexmedetomidine, dolasetron, efavirenz, eliglustat, epirubicin, eribulin mesylate, ezogabine(retigabine), famotidine, felbamate, fingolimod, flupentixol, gemifloxacin, granisetron, hydrocodone-ER, iloperidone, imipramine (melipramine), isradipine, ketanserin, lapatinib, lenvatinib, leuprolide, lithium, melperone, midostaurin, mifepristone, mirabegron, mirtazapine, moexipril/HCTZ, necitumumab, nicardipine, nilotinib, norfloxacin, nortriptyline, nusinersen, ofloxacin, osimertinib, oxytocin, paliperidone, palonosetron, panabinostat, pasireotide, pazopanib, perflutren lipid microspheres, perphenazine, pilsicainide, pimavanserin, pipamperone, promethazine, prothipendyl, rilpivirine, risperidone, romidepsin, sertindole, sorafenib, sunitinib, tamoxifen, tipiracil/trifluridine,tizanidine, tolterodine, toremifene,trimipramine, tropisetron, vardenafil, vemurafenib, venlafaxine, vorinostat, ziprasidone |
| MATE1/2 substrates[3] | Acyclovir, cephalexin, cimetidine, fexofenadine, ganciclovir, glycopyrronium, metformin, pindolol, plisicainide, ranitidine, topotecan, varenicline |

| | |
|---|---|
| OCT1/2 substrates[4] | Amantadine, 6-beta-hydroxycortisol, carboplatin, cisplatin, cephalexin, cephradine, , , , ipratropium, lamivudine, linagliptin, metformin, , oxyplatin, oxybutynin, phenformin, picoplatin, pilsicainide, pindolol, , ranitidine, sorafenib, tropisetron, trospium, umeclidinium,, , and zidovudine |
| BCRP substrates | Daunorubicin, dolutegravir, doxorubicin, hematoporphyrin, imatinib, methotrexate, mitoxantrone, pitavastatin, rosuvastatin, irinotecan, ethinyl estradiol, simvastatin, sulfasalazine, sofosbuvir, tenofovir, topotecan, venetoclax |

[1] Sensitive substrates: Drugs whose plasma AUC values have been shown to increase 5-fold or higher when co-administered with a potent inhibitor.[2] The list provided is as of January 2018. Check https www crediblemeds.org/healthcare-providers/drug-list for the most updated list.
[3] MATE1 and MATE2 share considerable substrate specificity.
[4] OCT1 and OCT2 share considerable substrate specificity.
[5] Lopinavir is prohibited when combined with ritonavir (see Table 14-1)

Source: Novartis PK Sciences Memorandum: Drug-Drug Interactions (DDI) and Co-medication Considerations for Novartis Clinical Trials (January 2018), which is compiled from Indiana University "Clinically Relevant" Flockhart Table™, University of Washington Drug Interaction Database, and FDA Drug Development and Drug Interactions: Table of Substrates, Inhibitors and Inducers.

Prohibited concomitant therapy: The following medications are prohibited during study treatment in the study (see table below). This list is not comprehensive and is only meant to be used as a guide.

- Strong inhibitors or inducers of CYP3A4/5
- Substrates of CYP3A4/5 with a narrow therapeutic index
- Medications that carry a known risk for QTprolongation
- Other investigational and antineoplastic therapies not part of thestudy
- Herbal medications/preparations, dietary supplements (except for vitamins) including, but not limited to: St. John's wort, Kava, ephedra (ma huang), gingko biloba, dehydroepiandrosterone (DHEA), yohimbe, saw palmetto, black cohosh and ginseng. Patients should stop using all herbal medications and dietary supplements at least 7 days prior to first dose of study treatment.

Drugs with QT prolongation: As far as possible, avoid co-administration of QT prolonging drugs or any other drugs with the potential to increase the risk of drug-related QT prolongation (e.g., via a potential DDI that increases the exposure of ribociclib or the exposure of the QT prolonging drug). A definitive list of drugs with a known risk, possible risk, or conditional risk of QT prolongation and/or Torsades de Pointes (TdP) is available online at www.qtdrug.org.

Medications with a known risk for QT prolongation are prohibited during study treatment.

The use of any other potential new concomitant medications may be discussed between the investigator and the PI on a case by case basis. The investigator should instruct the patient to notify the study site about any new medications including vitamins, supplements and herbal supplements he/she takes after the start of the study drug.

**Table 9. Prohibited drugs and therapy requiring caution and/or action**

| Category | Drug Name |
|---|---|
| Strong CYP3A4/5 inhibitors | Atazanavir/ritonavir, boceprevir, clarithromycin, cobicistat, conivaptan, danoprevir/ritonavir, darunavir/ritonavir, elvitegravir/ritonavir, grapefruit juice, idelalisib, indinavir, indinavir/ritonavir, itraconazole, ketoconazole, lopinavir/ritonavir, mibefradil, nefazodone, nelfinavir,ombitasvir/paritaprevir/dasabuvir/ritonavir (VIEKIRA PAK), posaconazole, ritonavir, saquinavir/ritonavir, telaprevir, telithromycin, tipranavir/ritonavir, troleandomycin, voriconazole |
| Strong CYP3A4/5 inducers | carbamazepine[3], enzalutamide, lumacaftor, mitotane, phenobarbital, phenytoin[3], rifabutin, rifampin (rifampicin)[3], St. John's wort (hypericum perforatum)[2,3] |
| CYP3A4/5 substrates with NTI[1] | Alfentanil, astemizole, cisapride, cyclosporine, diergotamine (dihydroergotamine), ergotamine, fentanyl, pimozide, quinidine, sirolimus, tacrolimus |
| Medications with a known risk for QT prolongation[4] | Amiodarone, anagrelide, arsenic trioxide, astemizole, azithromycin, chloroquine, chlorpromazine, cilostazol, ciprofloxacin, cisapride, citalopram, clarithromycin, disopyramide, dofetilide, domperidone, donepezil, dronedarone, droperidol, erythromycin, escitalopram, flecainide, fluconazole, gatifloxacine, grepafloxacin, halofantrine, haloperidol, ibutilide, levofloxacin, levomepromazine, levosulpiride, methadone, moxifloxacin, ondansetron, oxaliplatin, papaverine HCl (intra-coronary), pentamidine, pimozide, procainamide, propofol, quinidine, roxithromycin, sevoflurane, sotalol, sulpiride, sultopride, terlipressin, terodiline, thioridazine, vandetanib |
| Herbal preparations/ medications | Herbal preparations/medications known as strong inducers or inhibitors of CYP3A4/5 or those with a known risk of QT prolongation are prohibited throughout the study. These herbal medications include, but are not limited to: St. John’s wort, Kava, ephedra (ma huang), gingko biloba, dehydroepiandrosterone (DHEA), yohimbe, saw palmetto, and ginseng. Patients should stop using these herbal medications 7 days prior to first dose of study drug. |
| Other investigational and antineoplastic therapies | Other investigational therapies must not be used while the patient is on the study. Anticancer therapy (chemotherapy, all SERMS (including raloxifene), biologic or radiation therapy, and surgery) other than the study treatments must not be given to patients while the patient is on the study medication. If such agents are required for a patient then the patient must be discontinued study drug. |
| | |

| Category | Drug Name |
|---|---|
| [1] NTI = narrow therapeutic index drugs whose exposure-response indicates that increases in their exposure levels by the concomitant use of potent inhibitors may lead to serious safety concerns (e.g., Torsades de Pointes) or drugs which have <2-fold difference in the minimum toxic concentrations and minimum effective concentrations in the blood.<br>[2] Herbal product<br>[3] P-gp inducer<br>[4] The list provided is as of January 2018.Check https www crediblemeds.org/healthcare-providers/drug-list for the most updated list.<br>As far as possible, avoid co-administration of QT prolonging drugs or any other drugs with the potential to increase the risk of drug-related QT prolongation (e.g., via a potential DDI that increases the exposure of ribociclib or the exposure of the QT prolonging drug). A definitive list of drugs with a known risk, possible risk, or conditional risk of QT prolongation and/or Torsades de Pointes (TdP) is available online at qtdrugs.org.<br>Source: Novartis PK Sciences Memorandum: Drug-Drug Interactions (DDI) and Co-medication Considerations for Novartis Clinical Trials (January 2018), which is compiled from Indiana University "Clinically Relevant" Flockhart Table™, University of Washington Drug Interaction Database, and FDA Drug Development and Drug Interactions: Table of Substrates, Inhibitors and Inducers. | |

### 5.2 Duration of Therapy

Patients will receive 6 cycles of platinum + taxane (days1, 8, 15 on a 28 day cycle) given with ribociclib (LEE-011) as above (section 5). Maintenance ribociclib (LEE-011) after completing chemotherapy in women achieving at least a PR will start 3 weeks after last dose of platinum + taxane.

To start maintenance therapy, the patient must meet the following criteria: ANC ≥1 x $10^9$/L, hemoglobin ≥9 g/dL and platelets ≥75 x $10^9$/L.

Initiation of maintenance therapy may be delayed for hematologic count recovery but must start within 6 weeks from last dose of platinum + taxane or patient will not be eligible for maintenance therapy. Maintenance ribociclib (LEE-011) will continue (3 weeks on, 1 week off ±2days) until time of progression. Each patient will have follow-up every 3 months.

### 5.3 Off Treatment Criteria

- Unacceptable toxicity necessitating dose modifications as outlined in section5.2
- Disease progression
- Inability to start maintenance therapy within 6 weeks of last dose of platinum +taxane

### 5.4 Duration of Follow-Up

Survival follow-up data will be collected 3 years from the start of trial intervention (actual) date for the final patient enrolled (3/19/2020), so until 3/19/2023.

### 5.5 Off Study Criteria

Patients can be taken off study at any time at their own request, or they may be

withdrawn at the discretion of the investigator for safety, behavioral or administrative

reasons. The reason(s) for discontinuation from study will be documented and may include:

- Patient withdraws consent (termination of treatment and follow-up); Loss of ability to freely provide consent through imprisonment or involuntary incarceration for treatment;
- Patient is unable to comply with protocol requirements;
- Treating physician judges continuation on the study would not be in the patient's best interest;
- Patient becomes pregnant (pregnancy to be reported along same timelines as a serious adverse event);
- Development of second malignancy (except for basal cell carcinoma or squamous cell carcinoma of the skin) that requires treatment, which would interfere with this study;
- Lost to Follow-up: If a research subject cannot be located to document PFS after 6 months, the subject will be considered "lost to follow-up"
- Termination of the study by The University of Michigan;
- Patient completes protocol treatment and follow-up criteria.

### 5.6 Patient Replacement

Patients enrolled who did not receive a dose of ribociclib (LEE-011) or platinum + taxane will not contribute to MTD determination and will be replaced

## 6.0 STUDY PROCEDURES

### 6.1 Screening/Baseline Procedures

Assessments performed exclusively to determine eligibility for this study will be done only after obtaining informed consent. Assessments performed for clinical indications (not exclusively to determine study eligibility) may be used for baseline values even if the studies were done before informed consent was obtained.

All screening procedures must be performed within 14 days (except for imaging (CT, MRI, or PET) which can be within 28 days) prior to registration unless otherwise stated. The screening procedures include:

**6.1.1** Informed Consent

**6.1.2** Medical history

Complete medical and surgical history, history of infections

**6.1.3** Demographics

Age, race, ethnicity

**6.1.4** Review subject eligibility criteria

**6.1.5** Review previous and concomitant medications

**6.1.6** Physical exam including vital signs (temperature, pulse, respirations, blood pressure), height, weight

**6.1.7** Performance status

Performance status evaluated prior to study entry based on ECOG assessment.

**6.1.8** Adverse event assessment

Baseline adverse events will be assessed. See Section 8.0 for Adverse Event monitoring and reporting.

**6.1.9** Laboratory assessment

- Complete blood count with differential (CBCD)
- Comprehensive metabolic panel (CMP) to include: albumin,alkaline phosphatase, ALT/SGPT, AST/SGOT, BUN, creatinine, electrolytes (sodium, potassium, calcium, chloride, bicarbonate), glucose, and total bilirubin.
- Magnesium
- CA125
- PT/INR, PTT
- Lipid panel
- Urinalysis

**6.1.10** Acquisition of previous tissue sample for correlative studies

See Section 10.0 for details.

**6.1.11** Pregnancy test (for females of childbearing potential) See Section 3.1.6 for definition.

**6.1.12** Baseline ECG performed in triplicate

**6.1.13** CT chest, abdomen and pelvis with and without contrast (CT C/A/P)

**6.1.14** Transthoracic echocardiogram (TTE) or MUGA

## 6.2 Procedures During Treatment

Screening labs and ECG performed within 14 days of starting treatment do NOT need to be repeated for Day 1, Cycle 1 unless clinically indicated Ribociclib (LEE-011) treatment with platinum + taxane

**Day 1 (± 2days) (week 1 ribociclib (LEE-011) + platinum + taxane)**

- Physical exam, vital signs, performance status
- CBCD
- CMP
- Magnesium
- CA125
- ECG
- Urinalysis

**Day 8** (± 2days) **(week 2 ribociclib (LEE-011) +** platinum + taxane**)**

- Physical exam, vital signs, performance status, ECG (for first2cycles only)
- CBCD
- CMP

**Day 15** (± 2days) **(week 3 ribociclib (LEE-011) +** platinum + taxane**)**

- Physical exam, vital signs, performance status, ECG (for the first 2 cycles only)
- CBCD
- CMP

**After 3rd and 6th cycles**

- CT C/A/P

**Ribociclib (LEE-011) Maintenance to start within 6 weeks of completing 6 cycles of chemotherapy in women achieving at least a PR**

**Day 1 of each 28 day cycle (± 2 days)**

- Physical exam, vital signs
- CBCD
- CMP
- CA125
- urinalysis
- ECG

- ***CBC, BMP every 2 weeks (± 2 days) during first 4 cycles of maintenance; weekly telephone toxicity checks for the first 2 cycles of maintenance then biweekly for the next 2 cycles of maintenance. Weekly contact (via phone, video, or office visit) for protocol compliance.

**Every 3 cycles**

- CT C/A/P

**Off Treatment, 30 (± 2 days) days post last dose study drug**

- Physical exam, vital signs, performance status
- CBCD
- CMP

All research procedures will take place at Magee Women's Hospital; the clinical care team will administer drug and investigative physicians will do physical exams, review concomitant medications, orders related to study and ECGs at some visits.
The Magee Women's Hospital Investigational Drug Service (IDS) will prepare the study drug which will be supplied by Novartis.

### 6.3 Follow-Up Procedures

Patients will be followed via medical record review every 3 months for disease progression and survival until March 2023 or patient meets documented end points (section 2.3-2.4). Follow up can be performed over the phone if necessary. The follow-up may be done at another clinic visit if subject is seeing a UPMC physician. The study team will interact with the clinical provider to obtain study outcomes, if subject chooses that option.

**Time and Events Table**

| Table 10 Study calendar during chemotherapy | | | | |
|---|---|---|---|---|
| | Pre-study++ | Day1 of each cycle(± 2days) | Days 8 and 15 of each cycle (± 2days) | After 3rd and 6th Cycles |
| **Assessment** | X | | | |
| **Informed Consent** | X | | | |
| **H&P, vitals, PS** | X | X | X*** | |
| **Toxicity Evaluations** | | X | X | |
| **CA125** | X | X# | | |
| **CBCD** | X | X# | X | |
| **CMP** | X | X# | X | |
| **Magnesium** | X | X# | | |
| **Urinalysis** | X | X# | | |
| **PT/INR, PTT** | X | | | |
| **Lipid panel** | X | | | |
| **TTE or MUGA** | X | | | |
| **Triplicate ECGs** | X | X# | X*** | |
| **bHCG** ** | X | | | |
| **Concomitant Medication Review** | X | X | X | |
| **Previous tissue sample acquisition** | X | | | |
| **CT Scan*** | X | | | X |
| **Platinum + Taxane §** | | X | X | |
| **ribociclib (LEE-011) §§** | | X | X | |

*7 days (±2 days) after completing cycles 3 and cycles 6; ** only applicable to women of childbearing potential (see section 3.1.6 for definition); § given on a weekly (days 1, 8, 15 of a 28 day cycle) (±2 days) dosing schedule for 6 cycles; §§ given days 1-4, 8-11 and 15- 18 with weekly platinum + taxane and on days 1-21 of a 28 day cycle as single agent maintenance; *** day 8 and 15 H&P, vitals, PS and ECG only for the first 2 cycles
++must be completed within 28 days for imaging and 14 days for other studies of enrollment #screening labs and ECG performed within 14 days of starting treatment do NOT need to be repeated on Day 1, Cycle 1 unless clinically indicated

**Table 11. Study calendar during maintenance ribociclib (LEE-011)**

| | | Table 11 Study calendar during maintenance ribociclib (LEE- 011) | | | | | |
|---|---|---|---|---|---|---|---|
| | Day 1 of each cycle (± 2days) * | Weekly for 1st 2 cycles of Mainten a nce | Every 2 weeks (± 2days) ** | Biweekly for cycles 3 and 4 | Every 3rd cycle | Off treatment | Every 3 months |
| H&P, vitals, PS | X | | | | | X | |
| Toxicity Evaluations | X | | | | | X | |
| Telephone AE Assessment | | X | X*** | X | | | |
| CA125 | X | | | | | X | |
| CBCD | X | | X | | | X | |
| CMP | X | | | | | X | |
| BMP | | | X | | | | |
| ECG | X | | | | | | |
| urinalysis | X | | | | | | |
| Concomitant Medication Review | X | | | | | X | |
| CT Scan | | | | | X | | |
| ribociclib (LEE-011) | X | | | | | | |
| Survival Follow-up | | | | | | | X |

*cycle = 4 weeks (3 weeks daily ribociclib (LEE-011), 1 week off (±2 days)); ** during first 4 cycles of maintenance; *** Weekly AE assessment, Drug compliance, Protocol changes (can be done via phone, video, or office visit

## 7.0 MEASUREMENT OF EFFECT

Although the clinical benefit of ribociclib (LEE-011) drug(s) has not yet been established in ovarian cancer, the intent of offering this treatment is to provide a possible therapeutic benefit, and thus the patient will be carefully monitored for tumor response and symptom relief in addition to safety and tolerability. Patients with measurable disease will be assessed by standard criteria. For the purposes of this study, patients should be re- evaluated every *12* weeks. In addition to a baseline scan, confirmatory scans will also be obtained *12* weeks following initial documentation of an objective response.

## 7.1 Antitumor Effect- Solid Tumors

Response and progression will be evaluated in this study using the criteria proposed by the gynecologic cancer intergroup incorporating the Response Evaluation Criteria in Solid Tumors (RECIST) Committee version 1.1 recommendations in combination with CA-125, termed the GCIG-RECIST criteria.

### 7.1.1 Definitions

Evaluable for toxicity. All patients will be evaluable for toxicity from the time of their first treatment with study drug.

Evaluable for objective response. Only those patients who have received at least 1 cycle of therapy, and have had their disease re-evaluated will be considered

evaluable for response. These patients will have their response classified according to the definitions stated below. (Note: Patients who exhibit objective disease progression prior to the end of cycle 1 will also be considered evaluable.)

### 7.1.2 Disease Parameters

Measurable disease. Measurable lesions are defined as those that can be accurately measured in at least one dimension (longest diameter in the plane of measurement is to be recorded) with a minimum size of:

- 10mm by CT scan (irrespective of scanner type) for studies with aslice thickness of ≤5mm or twice the slice thickness orMRI
- 10mm caliper measurement by clinical exam (lesions which cannot be accurately measured with calipers should be recordedas non-measurable)
- 20mm by chest X-ray (if clearly defined and surrounded by aeratedlung)

All tumor measurements must be recorded in millimeters (or decimal fractions of centimeters).

Malignant lymph nodes: To be considered pathologically enlarged and measurable, a lymph node must be ≥15mm in short axis when assessed by CT scan (CT scan slice thickness recommended to be no greater than 5 mm). At baseline and in follow-up, only the short axis will be measured and followed.

Non-measurable disease: All other lesions (or sites of disease), including small lesions (longest diameter <20 mm with conventional techniques or <10 mm using CT scan), are considered non-measurable disease. Bone lesions, leptomeningeal disease, ascites, pleural/pericardial effusions, lymphangitis cutis/pulmonitis, inflammatory breast disease, abdominal masses (not followed by CT or MRI), and cystic lesions are all non-measurable.

Target lesions: All measurable lesions up to a maximum of 2 lesions per organ and 5 lesions in total should be identified as target lesions and recorded and measured at baseline. Target lesions should be selected on the basis of their size

(lesions with the longest diameter), be representative of all involved organ, but in addition should be those that lend themselves to reproducible repeated measurements.

Lymph nodes merit special mention since they are normal anatomical structures which may be visible by imaging even if not involved by tumor. Pathological nodes which are defined as measurable and may be identified as target lesions must meet the criterion of a short axis of ≥15mm by CT scan. Only the short axis of these nodes will contribute to the baseline sum. The short axis of the node is the diameter normally used by radiologists to judge if a node is involved by solid tumor. Nodal size is normally reported as two dimensions in the plane in which the image is obtained (for CT scan this is almost always the axial plane; for MRI the plane of acquisition may be axial, sagittal or coronal). The smaller of these measures is the short axis. For example, an abdominal node which is reported as being 20mm x 30mm has a short axis of 20mm and qualifies as a malignant,

measurable node. In this example, 20mm should be recorded as the node measurement. All other pathological nodes (those with short axis ≥10mm but <15 mm) should be considered non-target lesions. Nodes that have a short axis <10mm are considered nonpathological and should not be recorded or followed. A sum of the diameters (longest for non-nodal lesions, short axis for nodal lesions) for all target lesions will be calculated and reported as the baseline sum diameters. If lymph nodes are to be included in the sum, then as noted above, only the short axis is added into the sum. The baseline sum diameters will be used as reference to further characterize any objective tumor regression in the measurable dimension of the disease.

Non-target lesions: All other lesions (or sites of disease) including pathological lymph nodes should be identified as non-target lesions and should also be recorded at baseline. Measurements are not required and these lesions should be followed as 'present', 'absent', or in rare cases 'unequivocal progression' (more details to follow). In addition, it is possible to record multiple non-target lesions involving the same organ as a single item on the case record form (e.g. 'multiple enlarged pelvic lymph nodes' or 'multiple liver metastases').

Tumor marker: CA125 may be used to determine disease recurrence, progression and assess response per GCIG-RECIST criteria. See appendix B for GCIG-RECIST specific criteria.

**7.1.3 Guidelines for Evaluation of Measurable Disease**

All measurements should be recorded in metric notation, using calipers if clinically assessed. All baseline evaluations should be performed as close as possible to the treatment start and never more than 4 weeks before the beginning of the treatment.

The same method of assessment and the same technique should be used to characterize each identified and reported lesion at baseline and during follow-up. Imaging based evaluation should always be done rather than clinical examination unless the lesion(s) being followed cannot be imaged but are assessable by clinical exam.

Clinical lesions: Clinical lesions will only be considered measurable when they are superficial and >10mm diameter as assessed using calipers (e.g. skin nodules). For the case of skin lesions, documentation by color photography including a ruler to estimate the size of the lesion is suggested. As noted above, when lesions can be evaluated by both clinical exam and imaging, imaging evaluation should be undertaken since it is more objective and may also be reviewed at the end of the study.

Chest X-ray: Chest CT is preferred over chest X-ray, particularly when progression is an important endpoint, since CT is more sensitive than X-ray, particularly in identifying new lesions. However, lesions on chest X-ray may be considered measurable if they are clearly defined and surrounded by aerated lung.

CT, MRI: CT is the best currently available and reproducible method to measure lesions selected for response assessment. This guideline has defined measurability of lesions on CT scan based on the assumption that CT slice thickness is 5mm or less. When CT scans have slice thickness greater than 5 mm, the minimum size for a measurable lesion should be twice the slice thickness. MRI is also acceptable in certain situations (e.g. for body scans).

Ultrasound: Ultrasound is not useful in assessment of lesion size and should not be used as a method of measurement. Ultrasound examinations cannot be reproduced in their entirety for independent review at a later date and, because they are operator dependent, it cannot be guaranteed that the same technique and measurements will be taken from one assessment to the next. If new lesions are identified by ultrasound in the course of the study, confirmation by CT or MRI is advised. If there is concern about radiation exposure at CT, MRI may be used instead of CT in selected instances.

Endoscopy, laparoscopy: The utilization of these techniques for objective tumor evaluation is not advised. However, they can be useful to confirm complete pathological response when biopsies are obtained or to determine relapse in trials where recurrence following complete response or surgical resection is an endpoint.

Tumor markers: CA125 can be used to measure response in patients without measurable disease or in combination with the above measurable disease criteria based on the GCIG-RECIST. Patients can be evaluated according to CA 125 only if they have a pretreatment sample that is at least twice the upper limit of the reference range and within 2 weeks before starting the treatment

### 7.1.4 Response Criteria

#### 7.1.4.1 Evaluation of Target Lesions

Complete Response (CR): Disappearance of all target lesions, determined by two separate observations conducted not less than 4 weeks apart. There can be no appearance of new lesions.

Partial Response (PR): At least a 30% decrease in the sum of the longest diameter (LD) of target lesions, taking as reference the baseline sum LD. There can be no appearance of new lesions.

Progressive Disease (PD): At least a 20% increase in the sum of the LD of target lesions, taking as reference the smallest sum LD recorded since the treatment started, or the appearance of one or more new lesions.

Stable Disease (SD): Neither sufficient shrinkage to qualify for PR nor sufficient increase to qualify for PD, taking as reference the smallest sum LD since the treatment started.

#### 7.1.4.2 Evaluation of Non-Target Lesions

Complete Response (CR): Disappearance of all non-target lesions and normalization of tumor marker level.

Incomplete Response/Stable Disease (SD): Persistence of one or more non-target lesion(s) and/or maintenance of tumor marker level above the normal limits.

Progressive Disease (PD): Appearance of one or more new lesions and/or unequivocal progression of existing non-target lesions.

**7.1.4.3 Evaluation of CA125**

Complete Response (CR): Normalization of CA125 levels. The response must be confirmed and maintained for at least 28 days. No new or progressive disease on imaging.

Partial Response (PR): At least a 50% reduction in CA 125 levels from a pretreatment sample but not reaching normalization. The response must be confirmed and maintained for at least 28 days. No new or progressive disease on imaging.

Progressive Disease (PD):

- Patients with elevated CA-125 pretreatment and normalization of CA-125 must show evidence of CA-125 greater than, or equal to, 2 times the upper limit of the reference range on 2 occasions at least 1 week apart or
- Patients with elevated CA-125 before treatment, which never normalizes, must show evidence of CA-125 greater than, or equal to, 2 times the nadir value on 2 occasions at least 1week apart or
- Patients with CA-125 in the reference range before treatment must show evidence of CA-125 greater than, or equal to, 2times the upper limit of the reference range on 2 occasions at least 1 week apart
- Response determined via measurable disease (measurement of target and non-target lesions) takes precedence over CA125 criteria

Stable Disease (SD): CA125 fluctuations not fitting above criteria

**7.1.4.3 Evaluation of Best Overall Response**

The best overall response is the best response recorded from the start of the treatment until disease progression/recurrence (taking as reference for progressive disease the smallest measurements recorded since the treatment started). The patient's best response assignment will depend on the achievement of both measurement and confirmation criteria. See appendix B for tabular GCIG-RECIST criteria for best overall response.

**7.1.5 Duration of Response**

Duration of overall response: The duration of overall response is measured from the time measurement criteria are met for CR or PR (whichever is first recorded) until the first date that recurrent or progressive disease is objectively documented

(taking as reference for progressive disease the smallest measurements recorded since the treatment started).

The duration of overall CR is measured from the time measurement criteria are first met for CR until the first date that recurrent disease is objectively documented.

Duration of stable disease: Stable disease is measured from the start of the treatment until the criteria for progression are met, taking as reference the smallest measurements recorded since the treatment started.

#### 7.1.6 Progression-Free Survival

Progression-free survival (PFS) is defined as the duration of time from start of treatment to time of progression.

### 7.2 Safety/Tolerability

Analyses will be performed for all patients having received at least one dose of study drug. The study will use the CTCAE version 5 for reporting of non-hematologic adverse events (http://ctep.cancer.gov/reporting/ctc.html) and modified criteria for hematologic adverse events.

## 8.0 ADVERSE EVENTS

### 8.1 Experimental Therapy (ribociclib (LEE-011))

**8.1.1** Contraindications

- Patients who do not fit inclusion/exclusion criteria (section 3)

**8.1.2** Drug Interactions

- Strong CYP3A inhibitors/inducers and CYP3A substrates with narrow therapeutic index

**8.1.3** Adverse Reactions (see section 8.3)

### 8.2 Adverse Event Reporting Requirements

Adverse event (AE) monitoring and reporting is a routine part of every clinical trial and is done to ensure the safety of subjects enrolled in the studies as well as those who will enroll in future studies using similar agents. Data on adverse events will be collected from the time of the initial study through 30 days after the last dose of study treatment or study intervention. Any serious adverse event that occurs more than 30 days after the last study treatment or intervention and is considered related to the study treatment or intervention must also be reported. Serious Adverse Events (SAEs) will continue to be followed until:

- Resolution or the symptoms or signs that constitute the serious adverse event return to baseline;
- There is satisfactory explanation other than the study treatment or intervention for the changes observed; or
- Death.

The investigator is responsible for the detection, documentation, grading and assignment of attribution of events meeting the criteria and definition of an AE or SAE. The definitions of AEs and SAEs are given below. It is the responsibility of the principal investigator to ensure that all staff involved in the trial is familiar with the content of this section.

Any medical condition or laboratory abnormality with an onset date before initial study treatment administration or intervention is considered to be pre-existing in nature. Any known pre-existing conditions that are ongoing at time of study entry should be considered medical history.

All events meeting the criteria and definition of an AE or SAE, as defined in Section 8.3, occurring from the initial study treatment administration or intervention through 30 days following the last dose of the study treatment or study intervention must be recorded as an adverse event in the patient's source documents and on the CRF regardless of frequency, severity (grade) or assessed relationship to the study treatment or intervention.

In addition to new events, any increase in the frequency or severity (i.e., toxicity grade) of a pre-existing condition that occurs after the patient begins study treatment or intervention is also considered an adverse event.

## 8.3 Definitions (adapted from NCI Guidelines: Adverse Event ReportingRequirements)

### 8.3.1 Adverse Event

An adverse event (AE) is any untoward medical occurrence in a patient receiving study treatment and which does not necessarily have a causal relationship with this treatment. An AE can be any unfavorable and unintended sign (including an abnormal laboratory finding), symptom, or disease temporally associated with the use of an experimental intervention, whether or not related to the intervention.

### 8.3.2 Serious Adverse Event

An adverse event is considered "serious" if, in the view of either the investigator, it results in any of the following outcomes:

- Death:
  If death results from (progression of) the disease, the disease should be reported as event (SAE) itself.

- A life-threatening adverseevent:
  An adverse even is considered 'life-threatening' if, in the view of either the investigator, its occurrence places the patient or subject at immediate risk of death. It does not include an adverse event that, had it occurred in a more severe form, might have caused death.

- Inpatient hospitalization or prolongation of existing hospitalization for ≥ 24 hours.

- A persistent or significant incapacity or substantial disruption of the ability to conduct normal life functions
- A congenital anomaly/birth defect
- Important medical event:
  Any event that may not result in death, be life-threatening, or require hospitalization may be considered serious when, based upon appropriate medical judgment, they may jeopardize the patient and may require medical or surgical intervention to prevent one of the outcomes listed in this definition of "Serious Adverse Event". Examples of such medical events include allergic bronchospasm requiring intensive treatment in an emergency room or at home; convulsions that do not result in inpatient hospitalization or the development of drug dependency or drug abuse.

The following hospitalizations are not considered to be SAEs:

- Visit to the emergency room or other hospital department < 24 hours, that does not result in admission (unless considered an important medical or life-threatening event)
- Elective surgery, planned prior to signing consent
- Admissions as per protocol for a planned medical/surgical procedure
- Routine health assessment requiring admission for baseline/trending of health status (eg, routine colonoscopy)
- Medical/surgical admission other than to remedy ill health and planned prior to entry into the study. Appropriate documentation is required in these cases.
- Admission encountered for another life circumstance that carries no bearing on health status and requires no medical/surgical intervention (eg, lack of housing, economic inadequacy, caregiver respite, family circumstances, administrative reason).
- Admission for administration of anticancer therapy in the absence of any other SAEs (applies to oncology protocols)

**8.3.3 Expected Adverse Events**

An adverse event (AE) is considered "expected" if:

- For approved and marketed drugs or devices, those adverse events are described in the approved Package Insert (Label).
- For investigational new drugs or devices, those adverse events are described in the FDA Investigator's Brochure.
- In clinical research studies, information on expected adverse events is also summarized in the protocol and in the consent document. See section 9.1 for the list of expected adverse events related to the drug under study.

**8.3.4 Unexpected Adverse Event**

An adverse event (AE) is considered "unexpected" if it is not described in the Package Insert, Investigator's Brochure, in published medical literature, in the protocol, or in the informed consent document.

## 8.4 Adverse Event Characteristics

### 8.4.1 CTCAEv5 Term

(AE description) and grade: The descriptions and grading scales found in the NCI Common Terminology Criteria for Adverse Events (CTCAE) version 5.0 will be utilized for AE reporting. All appropriate treatment areas should have access to a copy of the CTCAE version 5.0. A copy of the CTCAE version 5.0 can be down loaded from the CTEP web site. (http://ctep.cancer.gov).

### 8.4.2 Attribution of the AE

The investigator or co-investigator is responsible for assignment of attribution.

Definite – The AE *is clearly related* to the study treatment
Probable – The AE *is likely related* to the study treatment
Possible – The AE *may be related* to the study treatment
Unlikely – The AE *is doubtfully related* to the study treatment
Unrelated – The AE *is clearly NOT related* to the study treatment

## 8.5 Serious Adverse Event Reporting Guidelines

All events meeting the definition of serious, as defined in section 8.3.2., should be reported according to the CRS departmental SAE checklist and CRS SAE form. Serious adverse events are collected from the date of the subject's first dose of treatment until 30 days after the last administration of study-related treatment. The initial SAE should be sent to the following within 24 hours of the Principal Investigator becoming aware:

1. Dr. Lan Coffman and Dr. Ronald Buckanovich
2. crssafetysubmissions@upmc.edu
3. Local Institutional Review Board and FDA, when reporting requirements are met
4. Novartis Pharmaceuticals Drug Safety and Epidemiology
   Department Fax Number: 877-778-9739

Note: Participating Sites must also submit events meeting reporting requirements to the contacts listed above, as well as following their internal processes and reporting to their IRB of record according to that IRB's reporting guidelines.

In addition to completing appropriate patient demographic and suspect medication information, the report should include as applicable the following information that is available at the time of the report within Sections B and C of the CRS SAE form:

- CTCAE term(s) and grade(s)
- Current status of study treatment(s)
- All interventions to address the event (testing and result, treatment and response)

- Hospitalization and/or discharge dates
- Event relationship to studytreatment

Follow-up reports:

All SAEs should be followed to resolution or stabilization. Additional information may be added to a previously submitted SAE report by adding to the CRS SAE form and submitting it as follow-up or creating a supplemental summary information and submitting is as follow-up with the original CRS SAE form. All follow-up forms must include the date the form is revised and should be submitted to the contacts listed above within 24 hours of the Principal Investigator becoming aware of the new information.

To ensure patient safety, every SAE, regardless of suspected causality, occurring after the subject has initiated treatment and until at least 30 days after the patient has stopped study treatment must be reported to Novartis within 24 hours of learning of its occurrence.

An SAE occurring at a different time interval or otherwise considered completely unrelated to a previously reported one should be reported separately as a new event. Any SAEs experienced after the 30 day safety evaluation follow-up period *(or 5 half-lives, if half-life is established,* whichever is longer) should only be reported to Novartis if the investigator suspects a causal relationship to the study treatment.

Follow-up information is submitted in the same way as the original SAE Report. Each re-occurrence, complication, or progression of the original event should be reported as a follow-up to that event regardless of when it occurs. The follow-up information should describe whether the event has resolved or continues, if and how it was treated, whether the blind was broken or not, and whether the patient continued or withdrew from study participation.

If the SAE is not previously documented in the Investigator's Brochure or Package Insert (new occurrence) and is thought to be related to the Novartis study treatment, an oncology Novartis Drug Safety and Epidemiology (DS&E) department associate may urgently require further information from the investigator for Health Authority reporting. Novartis may need to issue an Investigator Notification (IN), to inform all investigators involved in any study with the same drug that this SAE has been reported. Suspected Unexpected Serious Adverse Reactions (SUSARs) will be collected and reported to the competent authorities and relevant ethics committees in accordance with Directive 2001/20/EC or as per national regulatory requirements in participating countries.

### *Pregnancies*

To ensure patient safety, each pregnancy occurring while the patient is on study treatment must be reported to Novartis within 24 hours of learning of its occurrence. The pregnancy should be followed up to determine outcome, including spontaneous or voluntary termination, details of the birth, and the presence or absence of any birth defects, congenital abnormalities, or maternal and/or newborn complications.

Pregnancy should be reported by the investigator to the Novartis Oncology Drug Safety and Epidemiology Department (DS&E) by fax **(fax: 877-778-9739)**. Pregnancy follow-up should include an assessment of the possible relationship to the study treatmentany pregnancy outcome. Any SAE experienced during pregnancy must be reported on the SAE Report Form.

**8.6 Routine Reporting**

All other adverse events- such as those that are expected, or are unlikely or definitely not related to the study participation- are to be reported annually as part of regular data submission.

**8.7 Reporting of Unanticipated Problems**

There are types of incidents, experiences and outcomes that occur during the conduct of human subject research that represent unanticipated problems but are not considered adverse events. For example, some unanticipated problems involve social or economic harm instead of the physical or psychological harm associated with adverse events. In other cases, unanticipated problems place subjects or others at increased risk of harm, but no harm occurs.

Upon becoming aware of any incident, experience, or outcome (not related to an adverse event) that may represent an unanticipated problem, the investigator should assess whether the incident, experience, or outcome represents an unanticipated problem. The incident, experience or outcomes is considered unanticipated if it meets all of the following criteria:

1. Unexpected (in terms of nature, severity, orfrequency);
2. Related or possibly related to participation in the research;and
3. Suggests that the research places subjects or others at a greater risk of harm than was previously known or recognized.

If the investigator determines that the incident, experience, or outcome represents an unanticipated problem, the investigator must report it to the IRB according to IRB reporting guidelines.

**8.8 Stopping Rules**

- If DLT rate is greater than the defined MTD (<25%) in the lowest dose level (dose level 1), the study will be stopped (for all individuals and cohorts).
  - This stop will be a final end of dosing

**9.0 DRUG INFORMATION**

## 9.1 Novartis investigational drug, LEE011

- Description: Oral LEE011 is available in the form of a hard gelatin capsule or film coated tablet at two dosage strengths: 50 mg and 200 mg. Storage conditions are described in the medication label. All dosages prescribed and dispensed to the patient and all dose changes during the study must be recorded.
- Classification - type of agent: orally bioavailable small molecule kinase inhibitor
- Mode of Action: specific inhibitory activity against CDK4/cyclinD1 and CDK6/cylinD3 complexes, with concentration resulting in 50% inhibition (IC50) values of 10 nM and 39 nM, respectively, in isolated enzyme assays. It is inactive

against the majority of other kinases.

- Pharmacokinetics: ribociclib (LEE-011) is mainly metabolized in the plasma with excretion largely in bile and minimally in urine. Following oral dosing, LEE011 plasma exposure (maximum plasma concentration [Cmax] and AUC) exhibited slightly over-proportional increases in exposure across the dose range tested (50 to 1200 mg). Steady-state was generally reached by Day 8 and the mean effective T1/2 based on accumulation ratio (T1/2, acc) was 32.6 hours at the 600 mg dose. The accumulation ratio based on AUC obtained in a dosing interval (Racc) across the studied doses ranged from 1.55- to 3.13-fold.

- Side Effects: In single agent phase I trials, a total of 308 patients have been treated. Based on study CLEE011X2101, at the recommended dose for expansion (RDE) he most common (in at least 20% of patients) adverse events (AEs) related to study treatment were (all grades; grades 3/4): nausea (48.6%; 1.4%), neutropenia (33.8%; 21.6%), fatigue (31.1%; 0%), white blood cell count decrease (31.1%; 13.5%), diarrhea (31.1%; 2.7%), vomiting (27%;0%), anemia (21.6%; 2.7%).

- Drug Interactions: drugs which are strong CYP3A inhibitors/inducers and CYP3A substrates with narrow therapeutic window (see appendix A). Drugs which prolong QTc must be used with caution as ribociclib (LEE-011) may also prolong theQTc.

- Storage and Stability: ribociclib (LEE-011) should be stored according to the storage conditions provided on the medication label. Detailed instructions regarding

preparation and administration of ribociclib (LEE-011) capsules will be provided to all Investigators separate from this protocol. Ribociclib (LEE-011) must be stored in a secure, limited access area.

- Preparation and Dispensing: The study drug (ribociclib (LEE-011)) must be received at the study site by a designated person, handled and stored safely and properly, and kept in a secured location to which only the pharmacist and designated assistants have access. Upon receipt, ribociclib (LEE-011) is to be stored according to the instructions specified on the drug labels. Clinical supplies are only to be dispensedin accordance with the protocol. The investigator, or designee, must maintain an accurate record of the shipment and dispensing of study drug. All drug supplies are to be used only for this protocol, and not for any other purpose. Unless specifically instructed by Novartis, the investigator must not destroy any drug labels or any partly used or unused drug supply unless required to destroy on-site per local regulations.

  Patients must be instructed to return unused study drugs to the site at discontinuation or completion of treatment. The site personnel must ensure that the appropriate dose of each study drug is administered and that the drug accountability is performed.

- Ribociclib (LEE-011) will be given AFTER completion of platinum + taxane infusion on days 1, 8 and 15 of each cycle

- Administration: ribociclib (LEE-011) must be taken as follows:
  - Patients should be instructed to take the ribociclib (LEE-011) capsules with a large glass of water (~250 ml) at the same time each day.
  - Ribociclib (LEE-011) can be taken without regard to meals; however dietary habits around the time of dosing should be as consistent as possible throughout the study.
  - Patients must avoid consumption of grapefruit, grapefruit hybrids, pummelos, star-fruit, Seville oranges or products containing the juice of each during the entire study and preferably 7 days before the first dose of study medication, due to potential CYP3A4 interaction with the study medications. Orange juice is allowed.
  - No herbal or dietary supplements are permitted, due to potential interactions with ribociclib; multivitamins are allowed.
  - Patients should be instructed to swallow the ribociclib (LEE-011) capsules whole and not to chew, crush or open them.
  - If vomiting occurs during the course of treatment, no re-dosing of the patient is allowed before the next scheduled dose
  - Any doses that are missed (not taken within 6 hours of the intended time) should be skipped and should not be replaced or made up on a subsequent day.
  - Swallow whole capsule. Missed doses should not be made up.

- Availability**:** ribociclib (LEE-011) will be provided by Novartis
  Under no circumstance will the study medication (ribociclib (LEE-011)) be used other than as directed by the protocol.

- Return and Retention of Study Drug: At the conclusion of the study, and, as appropriate during the course of the study, the investigator will return all used and

unused study drug unless required to destroy on-site per local regulations, packaging, drug labels, and a copy of the completed drug accountability to the Novartis.

- Drug Accountability: The investigator, or a responsible party designated by the investigator, must maintain a careful record of the inventory and disposition of the investigational drug ribociclib (LEE-011). The drug accountability records will capture drug receipt, drug dispensing, drug return and final disposition.

## 9.2 Chemotherapeutics

- Commercial supply of carboplatin, cisplatin, paclitaxel, and docetaxel will be used and prepared/administered per institutional standards and the package insert

# 10.0 CORRELATIVES/SPECIAL STUDIES

The goal of the planned laboratory correlative studies is to explore the correlation between Rb mutational status, NFAT nuclear localization and response to ribociclib (LEE-011) therapy. Submission of archived samples for correlative studies is mandatory.

## 10.1 Sample Collection Guidelines

Frozen or paraffin imbedded tissue from the patient's initial tumor biopsy, debulking surgery or subsequent biopsies will be obtained. Samples will be labeled with the subject's coded study number and collection date and delivered for analysis is to:

Magee Women's Research Institute
401b, 204 Craft Ave
Pittsburgh, PA

Preparation and shipment will be as per standard operating procedures for frozen and paraffin embedded tissue.

Samples will be collected at the time of patient enrollment.

Assay Methodology:

- RB mutational assessment: phenol-based DNA extraction will be performed on either frozen and/or paraffin embedded tissue. PCR utilizing standard primer pairs will be performed to capture the most frequent Rb mutations (as perTCGAdata)[12]
- NFAT IHC: IHC will be performed on paraffin embedded tissue with a commercially available and internally validated antibody (abcam). Nuclear staining score (0-3+) will be assessed by a blinded independent pathologist (scores 2-3+ considered positive).

## 10.2 Specimen Banking

Patient samples collected for this study will be retained at Magee-Womens Research Institute. Specimens will be stored indefinitely or until they are used up. If future use is denied or withdrawn by the patient, best efforts will be made to stop any additional studies and to destroy the specimens.

Specimens being stored long-term for potential use not outlined in the protocol are subject to University Policy Governing Tissue Sample Collection, Ownership, Usage, and Disposition within all UPMC Research Repositories.

# 11.0 STATISTICAL CONSIDERATION

## 11.1 Study Design/ Endpoints

### 11.1.1 Endpoint(s)

- Primary Endpoint: Dose-limiting toxicity
- Secondary Endpoints: Clinical response (CR), progression-free survival (PFS) and overall survival (OS)
- Correlative/Exploratory Endpoints: Determine level of association between baseline Rb mutational status as measured via PCR and NFAT nuclear expression via IHC from frozen or paraffin embedded tissue from the patient's initial tissue diagnosis, debulking surgery or biopsy tumor samples with RR and PFS clinical endpoints.

### 11.1.2 Study Design

- The trial will be monitored using a modification of the Continual Reassessment Method, called Time-to-Event CRM or TITE-CRM. The TITE-CRM method assumes a model for the time to occurrence of toxic response as a function of dose, and allows information from all patients enrolled in the trial to be employed when allocating a new patient to a dose level. Because this method is very flexible in terms of the number of patients treated at each dose, subjects may be continuously recruited throughout the trial, without recruitment pauses, as long as patients are treated at a dose consistent with the current safety profile.

- The definition of dose-limiting toxicity (DLT) for this trial is defined in section 5.0.2 above.

- The acute observation period for toxicity is defined as the first two (28-day) treatment cycles, a 56-day time period.

- The target rate for dose-limiting toxicity is 25%. This level was selected based the definition of DLT events and consideration of the available literature on platinum + taxane regimens and single agent ribociclib (LEE-011) in this patient population (point to references). The target rate will define the 'maximum tolerated dose' (MTD) dose of ribociclib (LEE-011) given on days 1-4, 8-11, and 15-18 with weekly carboplatin+ Taxol (given days 1, 8, and 15 of a 28-day cycle), as specified inSection5.

- Thus, expected rates of acute toxicity have been estimated based upon previous treatment experience with platinum + taxane for these indications, and clinically acceptable maximal levels of toxicity. These rates will be re-estimated throughout the conduct of this trial as treatment experience is accrued.

### 11.1.3 Dose allocation

Phase I dose levels corresponding to expected toxicity rates are presented in the following table. Ribociclib (LEE-011) will be escalated in this protocol as shown below. Intrasubject escalation of the ribociclib (LEE-011) dose is NOT allowed.

| **Regimen level** | **Ribociclib (LEE-011)** QD on D1-4, D8-11, & D15-18 | **Carboplatin Weekly** | **Taxol** QD on D1, D8, D15, & D28 | **Expected probability of DLT** |
|---|---|---|---|---|
| 1** | 200 mg | AUC 2 | 60 mg/m$^2$ | 10% |
| 2 | 400 mg | AUC 2 | 60 mg/m$^2$ | 20% |
| 3 | 600 mg | AUC 2 | 60 mg/m$^2$ | 30% |

**The initial trial regimen dose is Level 1.

Doses will be allocated to patients according to the TITE-CRM criterion, as summarized here.

- When a patient presents for enrollment on this trial, the probability of toxicity will be estimated for each dose level, based upon the initial expectations of toxicity and the incidence of toxicity in patients already treated, weighted by the amount of time those patients have been followed during the acute observation period. The level that has estimated toxicity closest to the target rate subject to conditions below, will be selected. The estimate will take into account the patients estimated treatment starting date.
- In the extremely rare event a patient is removed from study prior to completing the acute observation period, for reason/s unrelated to toxicity, the patient will be considered not tohave experienced a DLT event. The patient will continue to contribute his or her weighted follow-up experience to the estimate of the probability of toxicity for each dose level for the duration of the study.
- The dose level cannot be escalated until at least two patients have been observed for the entire acute toxicity period (56 days).
- Dose escalation is restricted to one level between adjacent patients.
- There is no restriction on the number of levels that the dose may be reduced betweenpatients.
- A maximum of 4 patients per calendar month can be enrolled until 6 patients have been treated on this trial.
- Patients 1 and 2 will be assigned to dose level 1.
- For patients 3 to 15, the dose level estimated to have probability of DLT closest to but not exceeding the target rate, 25%, by more than 5% (i.e. 30% predicted probability) will be assigned to the newly enrolled patients.
- For patients 16 to 40 patients, the dose level with probability of DLT closest to, but not exceeding the target rate, 25%, will be assigned the newly enrolled patient.
  - Starting with the dose assignment for the 7$^{th}$ patient to be treated on this trial, if all dose levels are estimated to be higher than our target rate (25%) then the trial will be closed to new accrual. Under this allocation schema, the dose level of ribociclib (LEE-011) will increase until toxicity is observed, and then will tend to vary around the level producing the target rate of toxicity. In order to allocate treatments, the protocol statistician must be notified promptly of all dose-limiting toxicities.

### 11.1.4 Cohort size

Subjects will be recruited as available and allocated to treatment according to the estimated toxicity rates. No formal cohort size is required; however, at least two patients must be observed for their entire acute toxicity period (first two cycles, 56 days) before dose escalation isallowed.

### 11.1.5 Evaluability

Any subject receiving at least one dose of ribociclib (LEE-011) will be considered evaluable for toxicity. Subjects not meeting this criterion will be replaced and will not influence the TITE-CRM dose assignment algorithm. Any subject who discontinues trial therapy after receiving at least one dose of ribociclib (LEE-011), but prior to completing the entire first two cycles of study treatment for reasons not due to toxicity, will also be replaced. These subjects will not count toward the sample size goal of 40 subjects; however, partial information from discontinuing subjects will be used by the TITE-CRM algorithm when making dose level assignments. At the trial's conclusion, posterior estimates for the probability of toxicity at each dose level will be made using only subjects with complete data (i.e. subjects that complete the acute observation period without a dose-limiting event, or those that experience a DLT).

### 11.1.6 Operating Characteristics

The operating characteristics of this trial design were evaluated using repeated simulations of possible trial conditions, both near to and far from our expectations for toxicity. Four trial scenarios are presented in the table below. First, the true probability of toxicity at each dose level is as expected. Second, the true probability of toxicity at each dose level is moderately lower than expected. Third, the true probability of toxicity is mildly lower than expected. Fourth, the true probability of toxicity is moderately higher than expected. The dose level at our target for toxicity is in gray.

| Regimen level | ribociclib (LEE-011) dose | Prior expectation for toxicity | Scenario 1 | Scenario 2 | Scenario 3 | Scenario 4 |
|---|---|---|---|---|---|---|
| 1 | 200 mg | 10% | 10% | 10% | 10% | 15% |
| 2 | 400 mg | 20% | 20% | 15% | 18% | 30% |
| 3 | 600 mg | 30% | 30% | 20% | 25% | 45% |

The following figure displays the operating characteristics for this trial design under scenarios 1 to 4. Scenario 1 is the most challenging, as our target is at the midpoint between two dose levels (2 and 3), our design identifies dose level 2 and 3 in 34.7% and 54.3% of simulations, respectively. The correct dose level is identified in 77.9%, 66.0%, and 55.7% of the simulated trial in scenarios 2, 3, and 4, respectively. On average 16, 17, 13, and 16 patients are treated at the MTD dose for simulation scenarios 1 to 4,respectively.

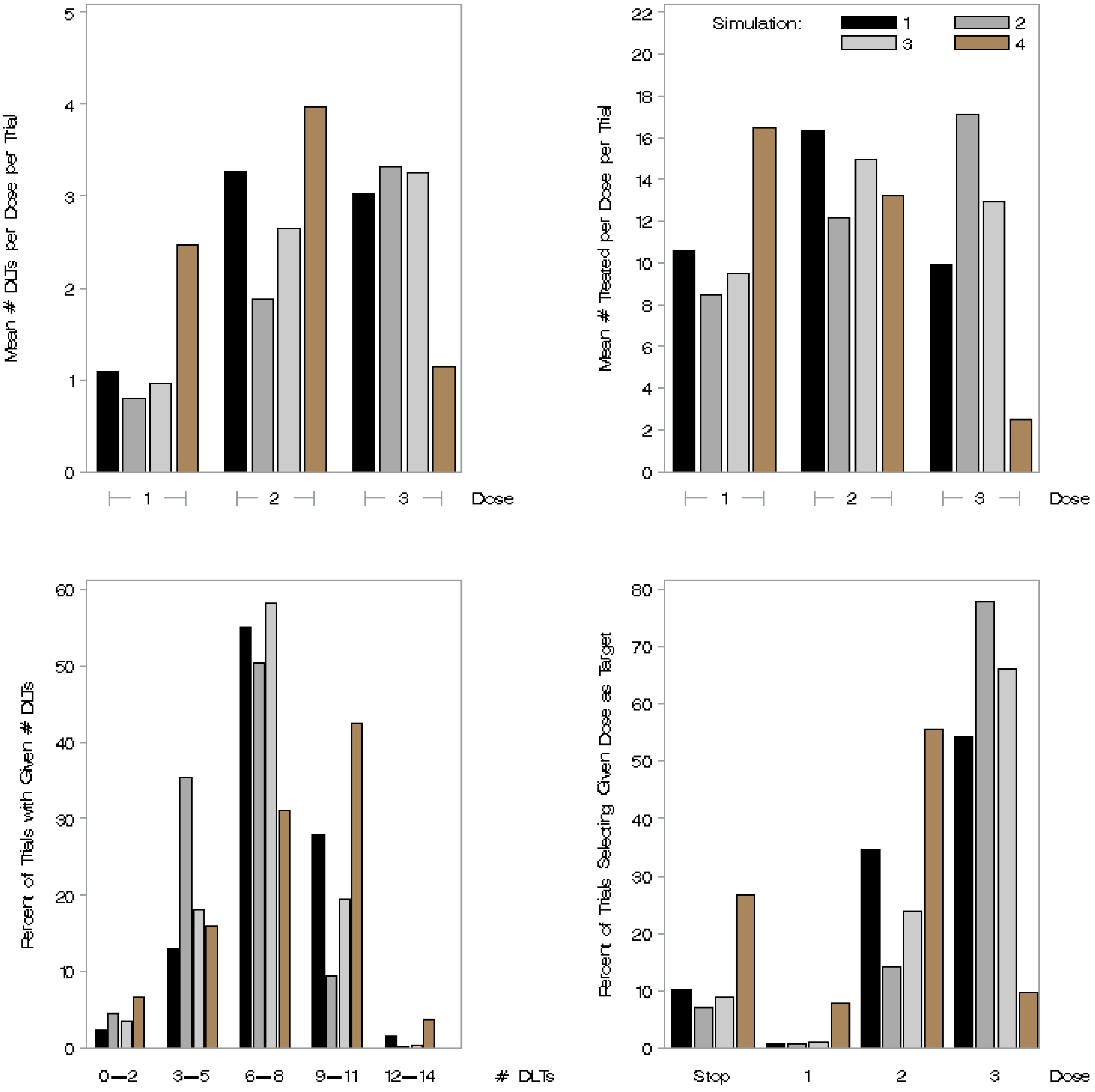


### 11.1.7 Early Stopping Guidelines

Safety will be monitored throughout the trial. Dose escalation will occur according to the dose escalation scheme described above. If any significant safety issues arise, a decision to modify or terminate the trial will be made.

The Intent-to-Treat (ITT) population of patients will be defined as all patients who receive at least one dose of ribociclib (LEE-011). The analysis will proceed as described above to establish the maximum tolerated dose.

Under the CRM paradigm, the relationship between dose and toxicity is summarized by a single-parameter (α) logistic model that represents the assumed relationship prior to the collection of any patient data. Information about the relationship between dose and toxicity can be summarized using

the distribution of the parameter, updated according to the current data. For instance, we can estimate the probability of toxicity at assigned doses and the subsequent target dose as a function of α based on the current data. The toxicity results will be summarized as follows.

The distribution of α updated according to the current data and the corresponding dose-toxicity model will be calculated. Predictive intervals concerning the toxicity parameter will be provided using this updated distribution of α.

The posterior distribution of toxicity, which displays the probability a future patient will experience toxicity at a given dose based on the current data, will be calculated for each tested level. The dose level of ribociclib (LEE-011) that produces the target rate of toxicity will be determined from this.

The proportion of subjects encountering toxicity at each dose level will be reported.

### 11.2 Sample Size/ Accrual Rate

Sample Size. This study will enroll 40 patients for the estimation of the dose-toxicity function. We expect to accrue approximately 2 patients per month hoping to complete accrual in about 20 months.

### 11.3 Stratification Factors

None.

### 11.4 Analysis of Secondary Endpoints

Secondary endpoints are clinical response (CR), progression-free survival (PFS), and overall survival (OS). The proportion of responding patients will be reported with exact 95% binomial confidence intervals. PFS and OS will be characterized by the product-limit method of Kaplan and Meier. Calculation of follow-up time will begin on the date of first study treatment.

Correlative studies: Initial tumor Rb mutational status will be correlated with response to ribociclib (LEE-011) (LEE-011) (TCGA estimate prevalence of RB mutations in ovarian cancer ~18% including deep deletions, missense mutations and truncation mutations [9]). PCR (from frozen or paraffin imbedded tissue) capturing the most common mutations found in the Rb gene will be used. We will also correlate nuclear NFAT expression levels in initial tumor sample via IHCwith response to ribociclib (LEE-011). IHC will be performed on paraffin imbedded tissue with an internally validated antibody and nuclear staining score (0-3+) will be assessed by a blinded independent pathologist (scores 2-3+ considered positive). Descriptive statistics will be generated, cross-tabulating Rb mutation status and NFAT expression level with best overallresponse.

## 12.0 DATA AND SAFETY MONITORING

Investigator/Sub-investigators, regulatory, CRS management, clinical research coordinators, clinical research associates, data managers, and clinic staff meet monthly in the UPMC Hillman Cancer Center Data Safety Monitoring Board (DSMB) to review and discuss study data to include, but not limited to, the following:

- • serious adverse events
- • subject safety issues
- • recruitment issues
- • accrual
- • protocol deviations

- • breaches of confidentiality

Minutes from the disease center DSMB meetings are available to those who are unable to attend in person.

All toxicities encountered during the study will be evaluated on an ongoing basis according to the NCI Common Toxicity Criteria version 4.0. All study treatment associated adverse events that are both serious and unexpected will be reported to the IRB. Any modifications necessary to ensure subject safety and decisions to continue, or close, the trial to accrual are also discussed during these meetings. If any literature becomes available which changes the risk/benefit ratio or suggests that conducting the trial is no longer ethical, the IRB will be notified in the form of an Unanticipated Problem submission and the study may be terminated.

All study data reviewed and discussed during these meetings will be kept confidential. Any breach in subject confidentiality will be reported to the IRB in the form of an Unanticipated Problem submission. The summaries of these meetings are forwarded to the UPMC Hillman Cancer Center DSMC which also meets monthly following a designated format.

For all research protocols, there will be a commitment to comply with the IRB's policies for reporting unanticipated problems involving risk to subjects or others (including adverse events). DSMC progress reports, to include a summary of all serious adverse events and modifications, and approval will be submitted to the IRB at the time of renewal.

Protocols with subjects in long term (survival) follow-up or protocols in data analysis only, will be reviewed bi-annually.

Both the UPMC Hillman Cancer Center DSMC as well as the individual disease center DSMB have the authority to suspend accrual or further investigate treatment on any trial based on information discussed at these meetings.

All records related to this research study will be stored in a locked environment. Only the researchers affiliated with the research study and their staff will have access to the research records.

## 13.0 REFERENCES

1. DeSantis, C.E., et al., *Cancer treatment and survivorship statistics, 2014.* CACancerJ Clin, 2014. **64**(4): p. 252-71.
2. Arai, F., et al., *Tie2/angiopoietin-1 signaling regulates hematopoietic stemcell quiescence in the bone marrow niche.* Cell, 2004. **118**(2): p.149-61.
3. Li, L. and R. Bhatia, *Stem cell quiescence.* Clin Cancer Res, 2011. **17**(15): p. 4936-41.
4. DeMichele, A., et al., *CDK 4/6 Inhibitor Palbociclib (PD0332991) in Rb+ Advanced Breast Cancer: Phase II Activity, Safety, and Predictive Biomarker Assessment.* Clin Cancer Res, 2015. **21**(5): p. 995-1001.
5. Infante, J., Shapiro, G, Witteveen, P, Gerecitano,JF, Ribrag, V, Chugh, R, Issa, I, Chakraborty, I, Matano, A, Zhao, X, Parasuraman, S, Cassier, P, *A phase I study of the single-agent CDK4/6 inhibitor LEE011 in pts with advanced solid tumors and lymphomas.* J Clin Oncol 32:5s, 2014 (suppl; abstr 2528^) 2014.
6. Taylor, M., Sosman, JA, Gonzalez, R, Carlino, MS, Kittaneh, M. Lolkema, MP, Miller, W, Marino, A, Zhang, V, Bhansali, SG, Parasuraman, S, Postow, M, *Phase Ib/II studyof LEE011 (CDK4/6 inhibitor) and LGX818 (BRAF inhibitor) in BRAF-mutant melanoma*,

in *Annals of Oncology*. 2014.
7. Bardia, A., Modi, S, Chavez-Mac Gregor, M, Kittaneh, M, Marino, AJ, Matano, A, Bhansali, S, Hewes, B, Cortes, J, *Phase Ib/II study of LEE011, everolimus, and exemestane in postmenopausal women with ER+/HER2-metastatic breast cancer.* J Clin Oncol 32:5s, 2014 (suppl; abstr 535) 2014.
8. Infante, J.R., et al., *A Phase I Study of the Cyclin-Dependent Kinase 4/6 Inhibitor Ribociclib (LEE011) in Patients with Advanced Solid Tumors and Lymphomas.* Clin Cancer Res, 2016. **22**(23): p. 5696-5705.
9. Tripathy, D., A. Bardia, and W.R. Sellers, *Ribociclib (LEE011): Mechanism of Action and Clinical Impact of This Selective Cyclin-Dependent Kinase 4/6 Inhibitor in Various Solid Tumors.* Clin Cancer Res, 2017. **23**(13): p. 3251-3262.
10. Hortobagyi, G.N., et al., *Ribociclib as First-Line Therapy forHR-Positive,Advanced Breast Cancer.* N Engl J Med, 2016. **375**(18): p. 1738-1748.
11. Parmar, M.K., et al., *Paclitaxel plus platinum-based chemotherapy versus conventional platinum-based chemotherapy in women with relapsed ovarian cancer: the ICON4/AGO-OVAR-2.2 trial.* Lancet, 2003. **361**(9375): p. 2099-106.
*12.* Cancer Genome Atlas Research, N., *Integrated genomic analyses of ovarian carcinoma.* Nature, 2011. **474**(7353): p. 609-15.

## 14.0 APPENDICES

### 14.1 Appendix A: GCIG-RECIST response criteria (Rustin et al.IntJ GynecolCancer 2011)

Evaluation of best overall response in patients *without* initial measurable disease and who are evaluable by CA 125

| CA 125 | Nontarget Lesions* | New Lesions | Overall Serological Response | Best Response for This Category Also Requires |
|---|---|---|---|---|
| Response and Normalized | CR | No | CR | Confirmed and maintained for at least 28 days |
| Response | Non-PD | No | PR | |
| Normalized but no response | Non-CR/Non-PD | No | SD | |
| Non-PR/non-PD | Non-PD | No | SD | |
| PD | Any | Yes or No | PD | |
| Any | PD† | Yes or No | PD | |
| Any | Any | Yes | PD | |

*Nontarget lesions include ascites and peritoneal thickening, which are not measurable according to RECIST.
†Unequivocal progression in nontarget lesions may be accepted as disease progression.
CR, Complete response; PD, progressive disease; PR, partial response; SD, stable disease.

**TABLE 3.** Best overall response in patients with measurable disease and who are also evaluable by CA 125

| Target Lesion* | Nontarget† | New Lesion | CA 125 | Overall Best Response | |
|---|---|---|---|---|---|
| CR | CR | No | Normal | CR | Best RECIST 1.1 response for CR and PR also requires it to be confirmed and maintained for at least 28 days if response is primary end point |
| CR | Non-CR Non-PD | No | Not PD | PR | |
| CR | CR | No | PR but not normal | PR | |
| CR | NE | No | PR | PR | |
| PR | Non-PD or NAE | No | Not PD | PR | |
| NAE | Non-PD | No | PR | PR | |
| PD or New >28 days from CA 125 PR‡ | | | PR | PR | |
| SD§ | Non-PD | No | PR | PR | |
| SD§ | Non-PD or NAE | No | Not PR and not PD | SD | |
| PD or New ≤28 days From CA 125 PR‡ | | | PR | PD | |
| PD | Any | Yes or No | Any | PD | |
| Any | PD | Yes or No | Any | PD | |
| Any | Any | Yes | Any | PD | |
| Any | Any | Yes or No | PD | PD | |

*Target lesions include up to 5 measurable lesions (2 per organ) as defined by RECIST 1.1.
†Nontarget lesions include ascites and peritoneal thickening which are not measurable according to RECIST 1.1.
‡Patients who have a CA 125 response that occurs more than 28 days from PD according to RECIST 1.1 are considered a PR, according to best response, but PD if the RECIST 1.1 PD is within 28 days of CA 125 response.
§The protocol should specify the minimum time interval between 2 measurements for classification as stable disease.
NE, Not evaluated; NAE, not all evaluated.

## 14.2 Appendix B: GCIG-RECIST definition of progressive disease (Rustin et al.IntJ Gynecol Cancer 2011)

Definition of progression after first-line therapy in ovarian cancer as proposed by the GCIG

| GCIG Subcategorized Group | RECIST Measurable/Nonmeasurable Disease | | CA 125 |
|---|---|---|---|
| A | Compared to baseline (or lowest sum while on study if less than baseline), a 20% increase in sum of diameters (RECIST 1.1 definition)<br>or<br>Any new lesions (measurable or nonmeasurable)<br>or<br>Unequivocal increase in nontarget disease<br>Date of PD: date of documentation of increase or new lesions | AND/ | CA 125 ≥ 2 × ULRR documented on 2 occasions*<br>Date of PD: first date of the CA 125 elevation to ≥ 2 × ULRR |
| B | As for A | OR | CA 125 ≥ 2 × nadir value on 2 occasions*<br>Date of PD: first date of the CA 125 elevation to ≥ 2 × nadir value |
| C | As for A | | As for A |

GCIG groups A, B, and C defined above.
CA 125 levels sampled after patients received mouse antibodies (unless the assay used has been shown not to be influenced by human antimouse antibody[4,5]) or if there has been medical and/or surgical interference with their peritoneum or pleura during the previous 28 days should not be taken into account.
*Repeat CA 125 any time but normally not less than 1 week after the first elevated CA 125 level.
ULRR, upper limit of response range.
